# Supplementary material for: Multifaceted intervention for the prevention and management of musculoskeletal pain in nursing staff: Results of a cluster randomized controlled trial
Source: PLoS One. 2019 Nov 18;14(11):e0225198. doi: 10.1371/journal.pone.0225198 (PMC6860418; doi:10.1371/journal.pone.0225198)
Supplement: S2 File — Evaluación de una intervención multifacética para reducir y gestionar el dolor musculoesquelético en personal de enfermería (INTEVAL_Spain). (PDF) [file pone.0225198.s002.pdf]

Evaluación de una intervención multifacética para reducir y gestionar el dolor musculoesquelético en personal de enfermería (INTEVAL_Spain)

Proyecto FIS PI14/01959

PROTOCOLO DE ESTUDIO (documento interno)

Barcelona, Mayo 2016

ÍNDICE

Equipo del proyecto INTEVAL_Spain 3
Justificación 4
Marco conceptual 4
Hipótesis y objetivos 10
Diseño del estudio 11
Intervención INTEVAL 11

- Ergonomía participativa 16
- Gestión de casos 20
- Promoción de la salud 23
- Integración de los componentes de la intervención INTEVAL 24
- Atención habitual de salud laboral 24

Recogida de datos 25
Evaluación de la intervención 26

- Evaluación de efectividad 27
- Evaluación de proceso 29
- Evaluación económica 31

Análisis estadístico 32
Requisitos éticos 32

- Confidencialidad de datos 32
- Voluntariedad 33
- Aprobación del comité ético de investigación clínica 33

Fortalezas y limitaciones 33
Bibliografía 33
Anexos 40

Anexo 1: Ficha informativa del proyecto inteval_spain y consentimiento informado 41
Anexo 2: Cuestionario basal 44
Anexo 3: Procedimiento para la aplicación del método ergopar 62

- Model d'acord del comitè de seguretat i salut per a l'aplicació del mètode ERGOPAR. 66
- Curs "ergonomia en el ámbito sanitario y método ergopar". 69
- Presentación "ergonomia laboral" 72
- Hoja de registro de problemas 85
- Rol y participación de los/las referentes de las unidades/ clústers 86
- Horas formación acreditada curso "Ergonomía en el ámbito sanitario y método ERGOPAR" 87
- Consentimiento informado 90
- Full de registre possibles solucions 91
- Informe de propuestas de medidas de mejora 92
- Taula de planificació (formato excel) 93

Anexo 4: Cuestionario gestión de casos 94
Anexo 5: Pauta para el cierre de casos y periodicidad del seguimiento motivacional 103
Anexo 6: Informe del comité ético de investigación clínica 104

EQUIPO DEL PROYECTO INTEVAL_Spain

Equipo de Investigación:

- Consol Serra (IP, coordinación), CiSAL-Universidad Pompeu Fabra/IMIM-Instituto Hospital del Mar de Investigaciones Médicas; Servicio de Salud Laboral, Parc de Salut Mar; CIBER de Epidemiología y Salud Pública. Barcelona.
- Pilar Peña, Servicio de Salud Laboral, Corporació Sanitaria Parc Taulí. Sabadell (Barcelona)
- José M Ramada. CiSAL-Universidad Pompeu Fabra/IMIM-Instituto Hospital del Mar de Investigaciones Médicas; Servicio de Salud Laboral, Parc de Salut Mar; CIBER de Epidemiología y Salud Pública. Barcelona.
- Antoni Merelles, Departamento de Enfermería y Podología, Universidad de Valencia. Valencia.
- Ana María Garcia, Departamento de Salud Pública, Universidad de Valencia. Valencia.
- Sergio Vargas-Prada, CiSAL-Universidad Pompeu Fabra/IMIM-Instituto Hospital del Mar de Investigaciones Médicas. Barcelona.
- Mercè Soler. CiSAL-Universidad Pompeu Fabra/IMIM-Instituto Hospital del Mar de Investigaciones Médicas; CIBER de Epidemiología y Salud Pública. Barcelona.

Colaboradores:

- Chelo Sancho, Servicio de Salud Laboral, Parc de Salut Mar MAR; IMIM-Instituto Hospital del Mar de Investigaciones Médicas. Barcelona.
- Anna Amat. CiSAL-Universidad Pompeu Fabra/IMIM-Instituto Hospital del Mar de Investigaciones Médicas. Barcelona.
- Olga Martinez. CiSAL-Universidad Pompeu Fabra/IMIM-Instituto Hospital del Mar de Investigaciones Médicas. Barcelona.
- Gemma Salvador. Agència de Salut Pública de Catalunya. Generalitat de Catalunya. Barcelona.
- Antonio Brieba. Walkim Barcelona.
- Georgina Badosa y Mònica Astals, instructoras de mindfulness. Parc de Salut Mar. Barcelona.

Asesores:

- Ana M García, Pere Boix, David Coggon, Ewan MacDonald, Sergio Vargas-Prada, Rosana Cortés, Fernando G. Benavides.

JUSTIFICACIÓN

Las afecciones musculoesqueléticas comprenden más de 150 diagnósticos que afectan el sistema locomotor, y van desde afecciones agudas y de corto plazo, como fracturas, esguinces y esguinces, hasta afecciones crónicas asociadas al dolor y la discapacidad. La característica más común de las afecciones musculoesqueléticas es el dolor, que puede reducir la capacidad de las personas para trabajar y participar en roles sociales, y puede tener un impacto en la salud mental y el bienestar, y más ampliamente en la sociedad.

La prevalencia del dolor musculoesquelético (DME) es muy alta y es la principal causa de incapacidad temporal en trabajadores sanitarios, especialmente en enfermeras y auxiliares de enfermería que trabajan en los hospitales [1], debido a la elevada exposición a factores de riesgo ergonómicos en sus tareas, como por ejemplo en la movilización de pacientes [2]. En España, como en otros países europeos, todos los empresarios tienen que organizar algún tipo de servicio de prevención según la exposición a los riesgos laborales y el tamaño de la empresa. Las tareas de estos servicios de prevención incluyen la evaluación de riesgos, la investigación de accidentes laborales, la vigilancia de la salud a través de exámenes de salud, y la prevención, formación e información que cubren los riesgos laborales y no laborales. Los Servicios de Prevención Externos suelen ser el principal proveedor de salud laboral para pequeñas y medianas empresas, mientras que en las grandes empresas, incluidos los hospitales, suelen tener un Servicio de Prevención Propio o Servicio de Salud Laboral (SSL), el cual ofrece mejores oportunidades para investigar y probar nuevos enfoques para mejorar la salud de los trabajadores.

MARCO CONCEPTUAL

Las interacciones complejas y dinámicas entre factores biológicos, psicológicos y sociales (incluidos los ambientales y culturales) tienen una fuerte influencia en la aparición y recurrencia de los trastornos musculoesqueléticos (TME) y la discapacidad resultante [3]. Aunque el modelo biopsicosocial [4] se ha convertido en el marco dominante a través del cual se conceptualiza la etiología y el pronóstico de los TME, su traducción a la práctica ha sido subóptima [5]. Tradicionalmente, se han investigado las causas de los TME a través de las disciplinas de biomecánica, fisiología, genética, epidemiología y rehabilitación. En su mayoría, cada una de estas disciplinas ha estudiado el desarrollo de TME de forma aislada de las otras disciplinas [6]. Sin embargo, éste enfoque fragmentado no ofrece la prevención

y el manejo óptimos de los TME. Con respecto a los TME que afectan a los trabajadores en relación con su capacidad para trabajar y la ocurrencia incapacidades temporales, los determinantes e interacciones involucrados en las vías causales son aún más complejos, como se muestra en la Figura 1.

Figura 1. Modelo conceptual para la causa de TME en trabajadores que afectan su capacidad para trabajar y/o para determinar la ausencia de enfermedad (modificado de National Research Council, 1999).

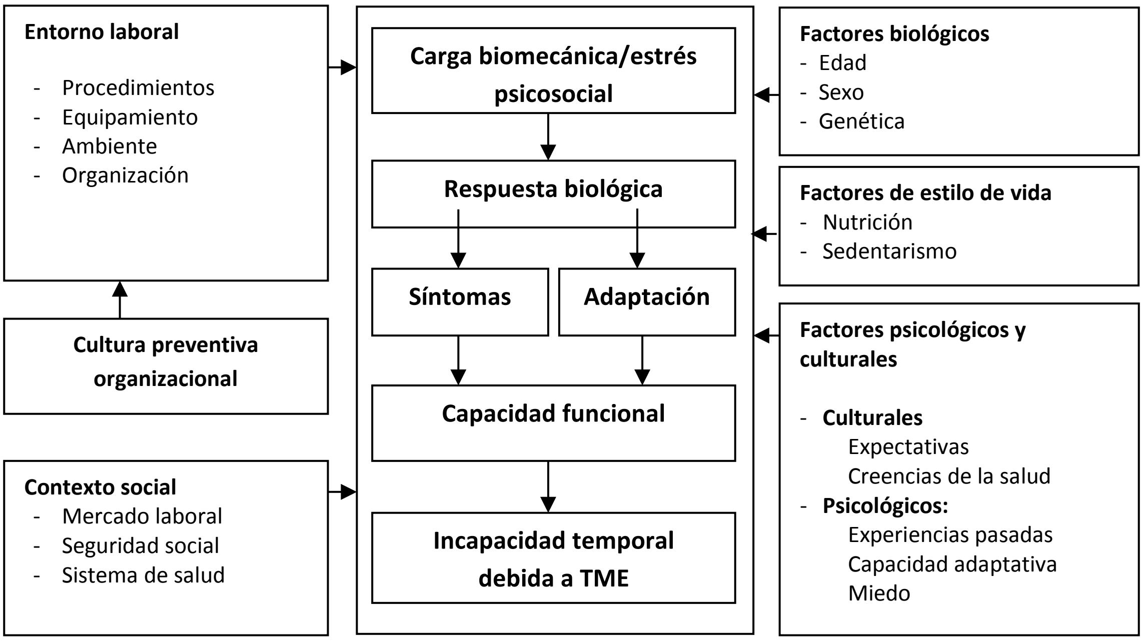

Como muestra la Figura 1, hay una serie de condiciones relacionadas con el entorno de trabajo, las tareas y la organización que se han vinculado con la aparición de TME que afectan a diferentes partes del cuerpo. Manipular objetos, aplicar fuerzas de tracción o empuje a herramientas o máquinas, posturas incómodas o exigencias posturales, como retorcer o doblar el tronco, trabajar en espacios confinados, la repetitividad o el uso de herramientas de vibración son ejemplos de condiciones de trabajo que se han asociado de manera constante con TME en la región cuello-hombro y en la región mano-muñeca [7,8]. Pero también se ha acumulado evidencia que relaciona la aparición de TME con factores estresantes psicosociales relacionados con el trabajo [9]. Una revisión reciente y un metanálisis respaldaron la conclusión de que los factores psicosociales relacionados con el

trabajo deben considerarse factores predictivos independientes del inicio y la persistencia de los TME, y son relevantes para los programas de prevención e intervención en seguridad y salud laboral [10]. Durante años, los mecanismos causales de esta relación fueron poco conocidos y algunos especularon que los entornos psicosociales pobres crearían una situación en la que los trabajadores tendrían más probabilidades de reportar lesiones y enfermedades. Sin embargo, la investigación experimental ha podido demostrar una vía biomecánica compleja a través de la cual los factores de riesgo psicosocial tienen un papel directo en las vías etiológicas que causan los TME [6].

El término de cultura de preventiva en organizaciones industriales fue introducido por primera vez por Zohar en los años 80, y desde entonces se ha desarrollado fructíferamente en relación con las prácticas de salud y seguridad laboral y los resultados a nivel de empresa [11-13]. Una revisión sistemática sobre la relación entre los indicadores de la cultura organizacional preventiva (definido como "percepciones colectivas de los empleados con respecto al compromiso de la organización con la seguridad") y los resultados de salud en los trabajadores de salud, encontró una mayor frecuencia de síntomas y TME en las enfermeras que trabajan en unidades y servicios de salud con peores indicadores de cultura organizacional preventive [14]. Además, se ha descrito una relación entre la incidencia y la duración de la incapacidad temporal y la situación laboral, las relaciones laborales y los sistemas de protección social prevalecientes en los países y períodos [15, 16], con marcadas variaciones, como durante los periodos de crisis económica [17].

Además, existen diferencias bien establecidas en la incidencia y la duración de los TME según la edad y el sexo, asociados con las diferencias físicas, antropométricas y hormonales, pero también probablemente debido a los diferentes perfiles de exposición por sexo y edad a los otros determinantes.

Por último, mayor en relación con la prevención secundaria y terciaria, existe un creciente interés en los factores culturales y psicológicos descritos en la Figura 1, que se reconocen cada vez más como los principales determinantes de la aparición y las consecuencias de los TME. Si bien los estudios observacionales han vinculado sistemáticamente los TME con ciertas actividades laborales, las intervenciones ergonómicas estándar en el lugar de trabajo a menudo no han logrado reducir la incapacidad temporal asociada a éstos [18]. Posiblemente los beneficios de una carga mecánica reducida estén contrarrestados por los impactos adversos en las creencias y expectativas de salud. Entre los pacientes que sufren

dolor musculoesquelético, aquellos que esperan que el síntoma persista y/o que no se mantengan activos, tienen peores resultados, incluso cuando se tienen en cuenta otras variables de pronóstico [19]. Otras creencias de salud, que en parte están determinadas por la cultura, también pueden predisponer a la aparición o persistencia de los síntomas y la incapacidad temporal asociada, y pueden contribuir a las grandes diferencias observadas en la prevalencia de dolor musculoesquelético incapacitante entre países y dentro de los países a lo largo del tiempo [20]. También hay evidencia que muestra que la tendencia a somatizar (es decir, experimentar angustia por los síntomas somáticos comunes) y el estado de ánimo bajo se asocian y predicen al dolor musculoesquelético [21].

Basado en el modelo conceptual descrito anteriormente y resumido en la Figura 1, INTEVAL_Spain ha sido diseñado para evaluar una intervención multicomponente en el lugar de trabajo que comprende tres componentes principales: un proceso participativo para cambiar y mejorar las condiciones ergonómicas en el lugar de trabajo, la gestión dirigida de los trabajadores con personal con TME, y un programa de promoción de la salud. La investigación sobre intervenciones en el lugar de trabajo que integra el modelo bio-psicosocial y el marco conceptual de las vías y los factores que contribuyen a los TME es casi inexistente, except por un ensayo controlado danés aleatorizado, multifacético y multifacético [22] que combina ergonomía participativa, entrenamiento conductual cognitivo y entrenamiento físico con el objetivo de prevenir el dolor lumbar y sus consecuencias entre los auxiliares de enfermería.

La Figura 2 muestra nuestro modelo conceptual en relación con las oportunidades de INTEVAL para evaluar el impacto de diferentes factores en la ocurrencia y las consecuencias de los TME.

Figura 2. Enfoque de la intervención multicomponente INTEVAL_Spain para prevenir y gestionar los TME en trabajadores.

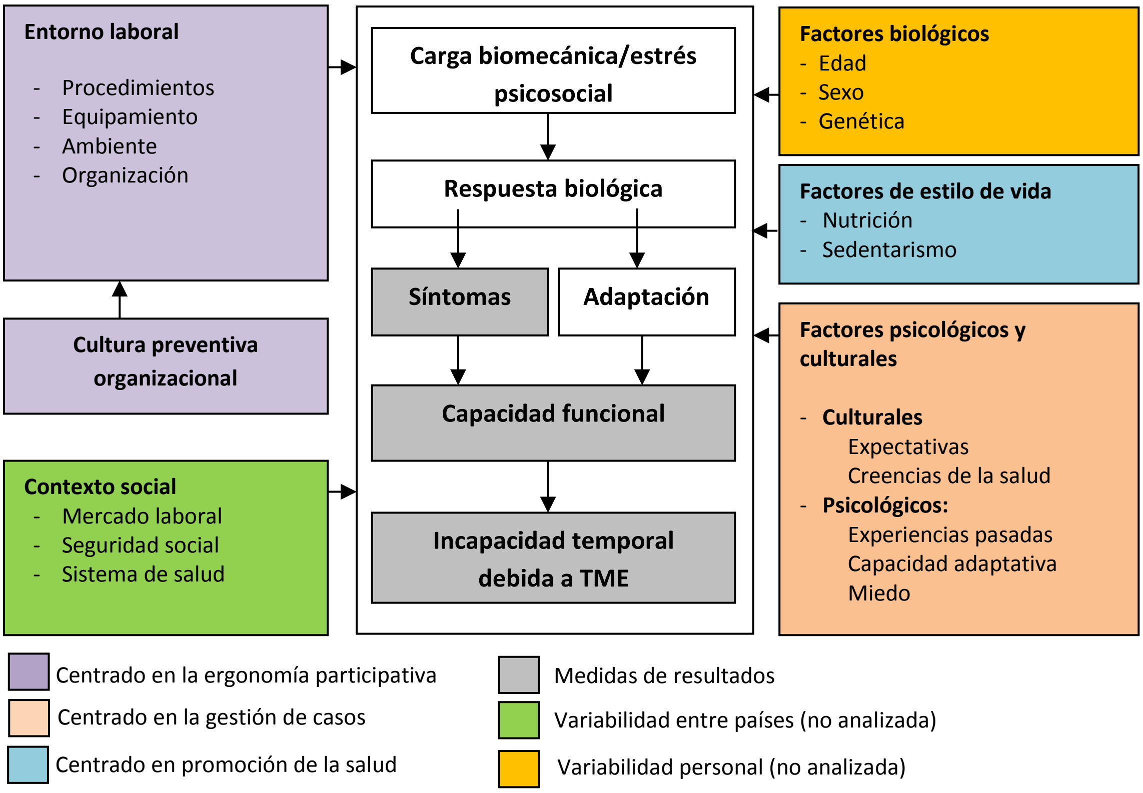

El proyecto INTEVAL se deriva de las siguientes hipótesis: (a) el modelo bio-psicosocial es el marco adecuado a través del cual se debe conceptualizar la etiología y el pronóstico de lso TME; (b) un "proceso de resolución de problemas" participativo basado tanto en el apoyo de la gerencia como en la participación de los trabajadores es un factor determinante para las prácticas exitosas de salud y seguridad destinadas a prevenir los TME relacionados con el trabajo; (c) la gestión específica de casos de trabajadores con TME funcionalmente limitantes favorecerá permanecer en el trabajo o regresar antes al mismo; (d) es necesaria una combinación de prevención primaria, secundaria y terciaria (incluido el control de peligros, cambios de comportamiento y culturales, diagnóstico temprano y seguimiento adecuado de los casos) para un control efectivo de la incapacidad laboral relacionada con los TME; (e) los recursos disponibles para la salud y la seguridad en las empresas europeas, independientemente de su actividad, tamaño o ubicación, se pueden utilizar con mayor eficiencia para prevenir los TME relacionados con el trabajo y para reducir las consecuencias de los TME en los empleados.

El modelo biopsicosocial considera simultáneamente los factores biológicos, psicológicos y sociales que pueden impactar en la salud y el bienestar general, vinculando los tres factores, y ha sido reconocido y aceptado por la OMS como se establece en la clasificación internacional de funcionamiento, discapacidad y salud [23]. Las intervenciones basadas en el enfoque biopsicosocial se han utilizado en el manejo de las incapacidades temporales [24] y en iniciativas de rehabilitación vocacional [25]. De los resultados de las intervenciones en su mayoría aisladas, la evidencia sugiere que una combinación de varios enfoques específicos podría dar una perspectiva de los mejores resultados [26].

Se ha observado que las intervenciones ergonómicas con mayor probabilidad de éxito son aquellas que involucran la cultura organizacional preventiva de la empresa y con un alto compromiso de los interesados [27]. Las mejoras ergonómicas en las condiciones de trabajo pueden producir mayores beneficios si son de naturaleza participativa [28], es decir, los propios empleados participan en la identificación de las modificaciones al trabajo que reducen la carga física y psicosocial y pueden hacer su trabajo más fácil y cómodo. Una revisión sistemática respalda la efectividad de las intervenciones ergonómicas participativas para reducir el dolor, los episodios de incapacidad temporal y los días de trabajo perdidos debido a los TME [29]. Además, hay indicios de que este tipo de intervención puede ser rentable [30]. Además, existe evidencia de que existen beneficios adicionales de las intervenciones participativas en el lugar de trabajo, incluidas las mejoras en la cultura organizacional preventive y las actitudes y comportamientos asociados, siempre que el tipo de participación haya sido previamente bien definido y organizado de acuerdo con el contexto específico de cada uno [31].

Además, la literatura científica proporciona varios ejemplos de programas exitosos de manejo de casos para trabajadores y personas en edad laboral con TME [25, 32]. La gestión de casos es el proceso colaborativo basado en la evidencia que evalúa, planifica, implementa, coordina, monitorea y evalúa las opciones y servicios requeridos para satisfacer las necesidades de salud, asistencia social, educación y empleo de una persona, utilizando la comunicación y los recursos disponibles para promover la calidad y resultados coste-efectivos [33]. Este proceso implica una serie de pasos dirigidos e individualizados que ayudan y apoyan la recuperación de los trabajadores con TME (o cualquier otra condición) hacia un retorno precoz y seguro a sus mejores capacidades funcionales y de trabajo posibles. La evidencia disponible sugiere que las intervenciones de manejo de casos reducen la duración de la ausencia por enfermedad, los síntomas de los TME y la incapacidad

temporal y mejoran la retención laboral [25, 32, 34]. También se ha sugerido que este tipo de programas pueden ser rentables [34].

La integración de actividades sobre estilos de vida saludables en el trabajo también es un componente importante de los programas de salud laboral para reducir los TME [35], por lo que también se incluirán diferentes estrategias que promueven la actividad física de los trabajadores, el bienestar emocional y la dieta saludable [36-39]. En cuanto a los trabajadores de la salud, la promoción de estilos de vida saludables debe ser una prioridad debido a su doble impacto en su propia salud y en la de los pacientes, fomentando estos estilos de vida en la población general.

El proyecto INTEVAL_Spain se encuentra en el espectro de "teoría a práctica" (theory to application). En este sentido, la participación de las partes interesadas clave en el lugar de trabajo en el desarrollo y la implementación de la intervención podría considerarse como una primera actividad de transferencia de "know-how", de la que se beneficiarán las empresas participantes. Somos conscientes de las enormes oportunidades para la transferencia de conocimiento dentro del Proyecto INTEVAL, respaldando lo que se ha denominado "salud y seguridad laboral basada en evidencia" [40].

HIPÓTESIS Y OBJETIVOS

Hipótesis

Una intervención en el lugar de trabajo que combine: la prevención primaria mediante a) ergonomía participativa para identificar y controlar las condiciones y situaciones de trabajo asociadas con la aparición de trastornos musculoesqueléticos en los trabajadores (prevención primaria), y b) un programa de promoción de la salud basado en el fomento de hábitos alimentarios saludables a través de la Dieta Mediterránea; y la prevención secundaria y terciaria mediante c) programa personalizado de gestión de casos para el diagnóstico precoz y el manejo personalizado de los trabajadores afectados por TME funcionalmente limitantes (prevención secundaria) y por la adecuación y facilitación del retorno al trabajo de los trabajadores en incapacidad temporal debido a TME (prevención terciaria); tendrá un impacto positivo en términos de reducción de al menos un 20% de la prevalencia del DME, así como incidencia y duración de la incapacidad temporal asociada y en términos de coste-efectividad y coste-beneficio.

Objetivos

El objetivo principal del Proyecto INTEVAL_Spain es evaluar una intervención multifacética formada por tres componentes diferentes y englobando los tres niveles de prevención para prevenir y gestionar el DME en el personal de enfermería.

Los objetivos específicos son:

1. Analizar la efectividad de la intervención en términos de salud medidos con la reducción del DME, reducción de la incidencia y duración de la incapacidad temporal, y mejora de la capacidad funcional para trabajar.
2. Analizar el proceso de la intervención a partir de los indicadores de contexto, reclutamiento, alcance, dosis oferta, dosis recibida, fidelidad, adherencia y satisfacción; y mediante información cualitativa, recogida en entrevistas semi-estructuradas.
3. Analizar el coste-efectividad y coste-utilidad de la intervención desde la perspectiva de sociedad y de sistema de salud, midiendo la efectividad en términos de DME y de calidad de vida.
4. Analizar el coste-beneficio de la intervención desde la perspectiva del hospital, midiendo los beneficios en términos de DME.

DISEÑO DEL ESTUDIO

Ensayo aleatorizado para clústeres (ECAC) de dos brazos: un grupo intervención y un grupo control que recibirá la intervención de forma tardía *(late intervention)*, donde los clústeres son unidades hospitalarias independientes y los participantes son personal de enfermería altamente expuesto a factores de riesgo ergonómicos en el trabajo.

Ámbito, selección de las empresas y cálculo de la muestra

Las empresas participantes en el ensayo deben cumplir cuatro condiciones que se consideran relevantes para los objetivos y viabilidad del estudio:

a) Tamaño (al menos 500 trabajadores).
b) Compromiso e interés explícito por parte de los responsables de la empresa para llevar a cabo la intervención y su evaluación.

c) Existencia a la empresa de un Servicio de Prevención Propio que mantenga registros rutinarios de calidad en salud laboral, e igualmente interesado en desarrollar la intervención.
d) Existencia de unidades de trabajo con exposición a riesgos musculoesqueléticos significativos, a partir de la información disponible del Servicio de Prevención.

Basándose en estos criterios, se han seleccionado dos hospitales terciarios de Barcelona ciudad y Sabadell (provincia de Barcelona) con un nivel similar de complejidad asistencial, con una población laboral de unos 3.500 trabajadores cada uno, de los cuales alrededor del 60% son personal de enfermería, y con un Servicio de Prevención Propio cada uno.

La tabla 1 muestra las empresas participantes en el proyecto.

Tabla 1. Descripción de las empresas participantes en el proyecto INTEVAL_Spain

Empresa	Sector	Descripción de la actividad de la empresa	Número de trabajadores (noviembre 2015)
Parc de Salut MAR (PSMAR)	Hospital tercer nivel	Centros de atención especializada de agudos, psiquiatría y sociosanitaria; atención primaria.	3.730
Corporació Sanitària Parc Taulí (CSPT)	Hospital tercer nivel	Centros de atención especializada de agudos, psiquiatría y sociosanitaria; atención primaria.	3.817
**Total**			**7.547**

Las estimaciones del tamaño de la muestra para el estudio se llevaron a cabo de acuerdo con los siguientes criterios:

a) Prevalencia de dolor lumbar de origen laboral (principal TME relacionado con el trabajo), según datos disponibles en la VII encuesta Nacional de Condiciones de Trabajo [1], estimada en 80% para los trabajadores sanitarios.

b) Impacto esperado de la intervención, estimado en términos de prevalencia de DME y basado en resultados observados en estudios previos [40, 41], habiéndose establecido en la hipótesis en una reducción del 20%.

c) Valores de alfa (error tipo I)=12:05, potencia estadística=0.80, coeficiente de correlación intraclase ICC=0,05.

Según estos criterios, y aplicando las órdenes de Stata Sampsi y sampclus, se requiere la participación de 294 sujetos. Se acordó el tamaño muestral mínimo de 300 sujetos distribuidos de la siguiente manera: 150 en el grupo de intervención y 150 en el grupo control.

Las unidades de las empresas participantes en el estudio suelen estar formadas por un número variable de entre 20 y 60 trabajadores. Por tanto, según la información disponible el número total de trabajadores incluidos en el estudio variaría entre 160 y 480 sujetos.

Selección de los clústeres y aleatorización

Se seleccionará al personal de enfermería (enfermero/as y auxiliares) de las unidades hospitalarias con riesgos musculoesqueléticos elevados debido a la exposición a factores ergonómicos en el lugar de trabajo y el tipo de pacientes (media y alta dependencia), de acuerdo a la información disponible y conocimiento de los técnicos de prevención del servicio de prevención de las empresas participantes.

Se acordaron 8 clústeres distribuidos de la siguiente manera (tabla 2):

- Parc de Salut Mar: 3 intervención + 3 control
- Corporació Sanitaria Parc Taulí: 1 intervención + 1 control

La distribución de las unidades a los grupos intervención y control se realiza mediante aleatorización simple estratificada por centro.

Tabla 2. Unidades/clústeres y número de trabajadores.

**Empresa**	**Unidades/clústeres**	**Número trabajadores**
Parc de Salut MAR (PSMAR)	Bloque quirúrgico	106
	UCI: unidad de curas intensivas	87
	UH04: cardiología y neumología	43
	UH30: traumatología y nefrología	44
	Llevants 3- 4: psicogeriatría	48
	UGA: unidad geriátrica de agudos	36
Corporació Sanitària Parc Taulí (CSPT)	UH06: traumatología y nefrología	57
	UH08: neurología	52
**Total**		**473**

Criterios de elegibilidad y reclutamiento de los participantes

Los sujetos de estudio son los profesionales de enfermería (enfermero/as y auxiliares), incluidos los empleados con una incapacidad temporal, de las unidades/clústeres incluidos y que acepten voluntariamente participar.

Son criterios de exclusión:

a) Tener un contrato temporal inferior a 3 meses no renovable en el tiempo.
b) Trabajar en varias unidades distintas.
c) Estar en situación de excedencia (período sabático).

Para reclutar a los participantes, se organizarán sesiones informativas del proyecto in situ en las unidades y todos los turnos de trabajo (mañana, tarde y noche), la entrega y recogida de los consentimientos informadosy de los cuestionarios basales:

a) Sesiones informativas iniciales: antes de comunicar las unidades que serán grupo intervención o control, se realizarán sesiones informativas en cada unidad y abarcando los diferentes turnos. Estas sesiones duraran entre 30 minutos y una hora, y en ellas el/la Champion juntamente con la persona del equipo investigador responsable de cada centro hospitalario explicaran que la unidad ha sido seleccionada para participar en un estudio de investigación, en el cual pueden ser grupo intervención o control y que los datos principales se recogerán principalmente a partir de cuestionarios.

b) Cumplimiento y entrega de los consentimientos informados (Anexo 1) y cuestionarios basales (Anexo 2): en la misma reunión se hará entrega de los consentimientos informados y de los cuestionarios basales. Éstos, podrán cumplimentarse en el momento o en un periodo máximo de dos semanas, para facilitar una mayor participación. Se habilitará un espacio en el control de cada unidad donde se colocarán consentimientos y cuestionarios para cumplimentar, así como para que los trabajadores entreguen el suyo ya rellenado. El/la Champion del proyecto pasará de forma regular a comprobar que quedan cuestionarios vacíos y a recoger los complimentados.

c) Después de llenar y devolver estos documentos, las unidades serán aleatorizadas y se informará sobre su condición de ser grupos de intervención o control.

d) En las unidades intervención se realizará otra sesión informativa, de entre 30 minutos y una hora, y en ésta el/la Champion conjuntamente con la persona del equipo

investigador responsable de cada centro hospitalario presentarán la intervención y las diferentes actividades a desarrollar.

Enmascaramiento

Este proyecto no es ciego:

a) A nivel de paciente: la condición de inclusión en la intervención o el grupo de control no se puede cegar, aunque los clústeres se aleatorizarán después de firmar el consentimiento informado y de cumplimentar el cuestionario basal.

b) A nivel de profesionales: los servicios prestados y los profesionales del SSL participantes no se pueden cegar ya que están implicados en la implementación de la intervención.

c) A nivel de análisis: el investigador qe realizará los análisis también estará a cargo de crear y limpiar la base de datos, así pues será conocedor de las unidades intervención y control para poder categorizarlas.

INTERVENCIÓN INTEVAL

La intervención INTEVAL cubre los tres niveles de prevención (prevención primaria, secundaria y terciaria), dura un año y posteriormente se implementa al grupo control.

Consta de 3 componentes: ergonomía participativa, gestión de casos y un programa de promoción de la salud (Figura 3).

Figura 3. Algoritmo del Proyecto INTEVAL_Spain.

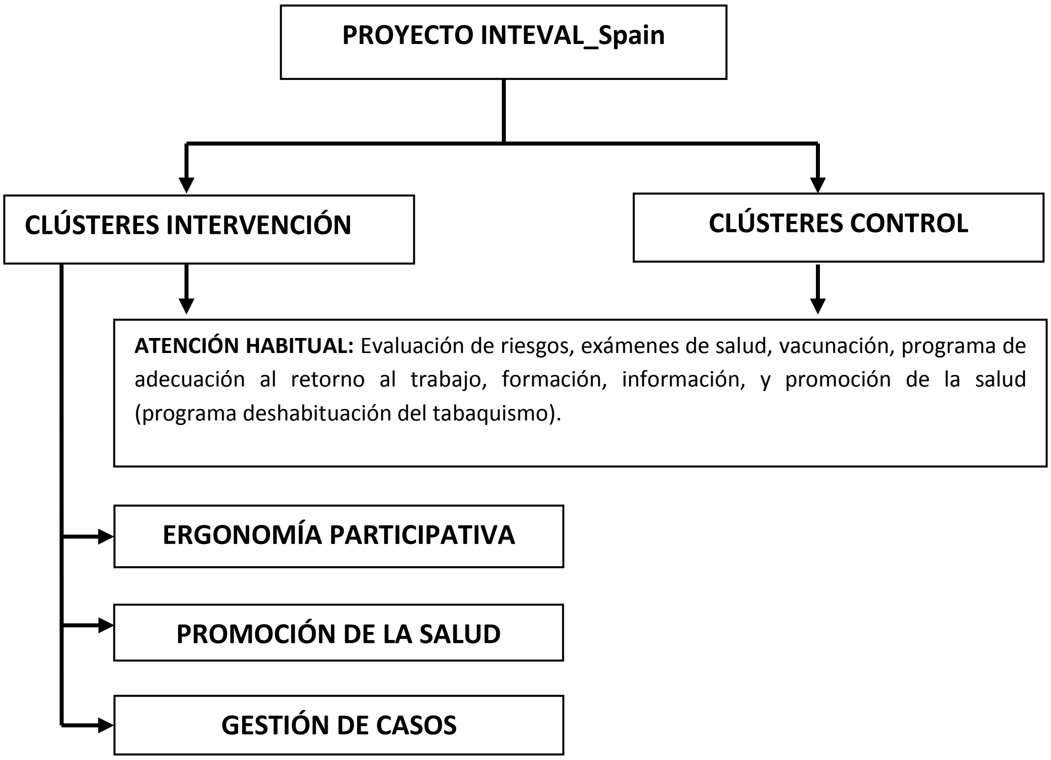

- **Ergonomía participativa**

Se seguirá la metodología ERGOPAR que consiste en un procedimiento estandarizado de ergonomía participativa en el trabajo que ha sido desarrollado y previamente pilotado [42, 43]. ERGOPAR implica la participación directa de los trabajadores en la identificación y solución de los problemas ergonómicos relacionados con el trabajo, dando un paso más allá de las prácticas ergonómicas más tradicionales basadas en gran parte las medidas técnicas, los cambios del entorno, y la provisión de información y formación a los trabajadores.

El equipo de la ergonomía participativa (EP) planificará el proceso bajo la supervisión del coordinador (*Champion*), que actuará como líder y facilitador de la intervención. El/la *Champion* organizará y dirigirá el trabajo a ser desarrollado. Este, puede ser un ergonomista, pero también podría ser otro profesional con experiencia en dirigir grupos de trabajo y/o la realización de intervenciones participativas en el lugar de trabajo.

<u>Metodología</u> (Figura 4 y Anexo 3):

1) Fase de preparación y constitución del **grupo de trabajo ERGO** para cada unidad/grupo de intervención, y que incluirá:

   a) Coordinador (*Champion*).
   b) Técnicos de prevención.
   c) Supervisores de la unidad/grupo de intervención.
   d) Referentes: trabajadores de la unidad de trabajo de intervención, en general 1 para cada turno de traajo (mañana, tarde, noche A y noche B), voluntarios y con funciones de dinamización de la intervención en cada unidad. Se les entregará un documento con sus funciones, firmarán un consentimiento informado y como compensación su participación será reconocida como 20 horas de formación acreditada por Formación Continuada del hospital, y contarán para la carrera profesional.
   e) Un Delegado de Prevención.

   Funciones: El grupo ERGO es el responsable del desarrollo e implementación de la intervención y sus miembros deberán recibir formación básica sobre ergonomía y métodos participativos. La intervención de EP implica la participación directa de los trabajadores en la identificación y solución de los problemas ergonómicos relacionados con el trabajo, dando un paso más allá de las prácticas ergonómicas más tradicionales basadas en gran parte en las mediciones técnicas, los cambios ambientales, y la provisión de información y formación a los trabajadores [44]. Como parte del Proyecto, el equipo Ergo se constituirá en cada empresa donde se desarrolle la intervención.

   El grupo ERGO mantendrá 3 reuniones de 1 hora cada una:

   - Reunión 1: formación del grupo ERGO por parte del técnico de prevención.
   - Reunión 2: identificación de los problemas ergonómicos en la unidad y su priorización
   - Reunión 3: identificación de las medidas de mejora/preventivas.

   Los participantes a cada reunión firmarán una hoja de asistencia que les contará a su vez como formación continuada.

En este punto, será necesario un acuerdo firmado con las empresas participantes (Dirección y Comité de Seguridad y Salud) para garantizar la correcta ejecución de la intervención.

2) Fase de diagnóstico:

Consiste en la distribución a los trabajadores de la unidad de trabajo de intervención de un cuestionario autocumplimentado previamente validado [42], en el que se recogen datos sobre daños musculoesqueléticos y exposición a factores de riesgo para los TME en el trabajo (ver más adelante en "Recogida de datos").

Esta información es posteriormente analizada y discutida por los componentes del grupo ERGO con la finalidad identificar factores de riesgo y puntos clave de mejora de las condiciones de trabajo, y su priorización.

3) Fase de tratamiento y círculos de prevención:

La fase de tratamiento consiste en que la información recogida en los cuestionarios es compartida y discutida con los trabajadores de la unidad de intervención en círculos de prevención, formados por todos los trabajadores de cada unidad.

Los círculos de prevención acuerdan y proponen, en reuniones informales dinamizadas por los referentes, un listado priorizado de medidas de mejora de las condiciones de trabajo para evitar o reducir los problemas ergonómicos identificados. El listado final es acordado en la reunión del grup ERGO. Estas medidas pueden incluir mejoras estructurales, técnicas, organizativas, formación/información y en el lugar de trabajo.

Figura 4. Intervención INTEVAL, componente ergonomía participativa, método ERGOPAR. Proyecto INTEVAL_Spain.

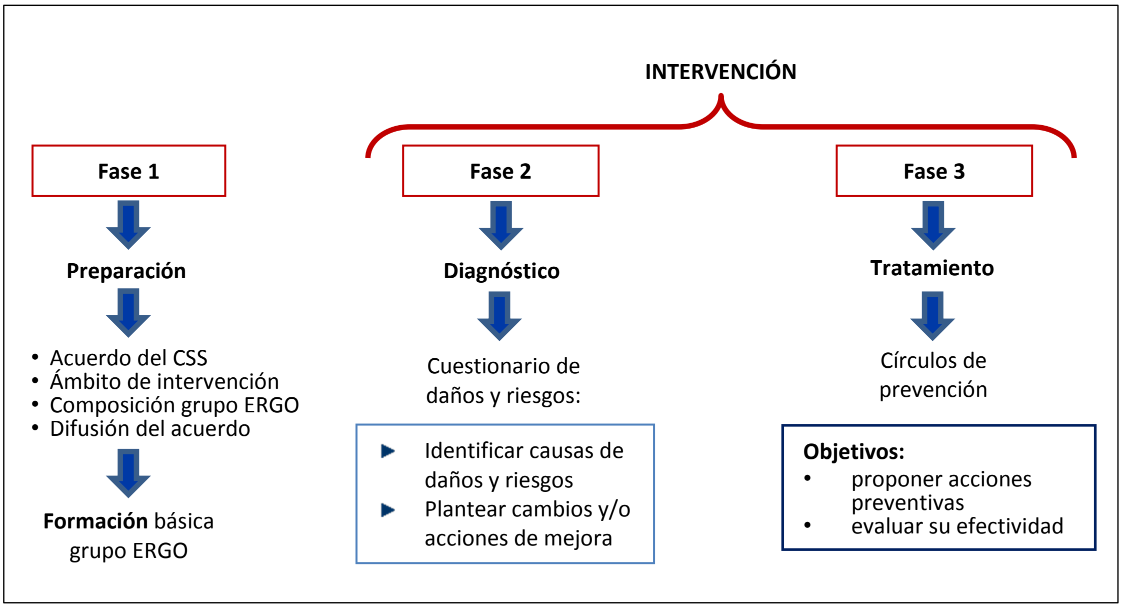

Implementación de las medidas de mejora propuestas, grupo operativo:

La propuesta de mejoras de la fase 3 se traslada al SSL, al Comité de Seguridad y Salud, a la Dirección y a los responsables de su implementación en la empresa. Se ha estimado entre seis y doce meses el tiempo requerido para completar todas las fases de la intervención en cada unidad de trabajo [43], aunque depende de otras variables (cultura organizacional preventiva en la empresa, complejidad de las medidas y de la propia empresa, etc.).

El grupo Operativo es dinámico y está formado por los responsables de la implementación de las medidas propuestas, y es coordinado y dinamizado por el Servicio de Prevención.

Como herramienta para el seguimiento de la implementación de las propuestas de mejora, el grupo operativo utilizará la tabla de planificación, de acuerdo al modelo en Excel diseñado para el estudio.

En las reuniones de coordinación entre el Comité de Seguridad y Salud, la Dirección y el SSL, se incorporará el seguimiento del ergopar en la agenda permanente de las reuniones.

- **Gestión de casos**

El componente de gestión de casos consiste en el abordaje individual de los trabajadores con un trastorno osteomuscular limitante con el objetivo de contribuir a la recuperación de los trabajadores con TME y, en aquellos en incapacidad temporal, a su retorno al trabajo en unas condiciones de salud adecuadas. Para ello, se construirá sobre los recursos y sistemas de gestión ya disponibles en el contexto de cada empresa.

La gestión de casos es definida por la Case Management Society UK como un proceso colaborativo que valora, planifica, implementa, coordina, monitoriza y evalúa las opciones y servicios requeridos para cubrir las necesidades individuales de salud, asistencia, educativas y de empleo, utilizando la comunicación y los recursos disponibles para promover resultados de calidad y costo-efectivos [32].

En el contexto del proyecto INTEVAL_Spain, la gestión de casos se entiende además como un servicio multidisciplinar y aplicado al marco conceptual del modelo biopsicosocial de la salud para gestionar en todas las fases de la enfermedad, en nuestro caso los TME, asi como medidas basadas en la evidencia para lograr una rehabilitación profesional (*vocational rehabilitation*). Se centrará en el trabajo y en el mantenimiento de los trabajadores en sus puestos de trabajo y la mejora de la capacidad funcional para trabajar. Implicará el contacto principalmente telefónico con los trabajadores afectados en combinación con entrevistas personales.

En esta intervención se contará con un **Gestor de Casos,** a quien se formará en base a los objetivos y componentes de esta intervención. No es necesario que tenga un perfil sanitario, sin embargo, sí que es recomendable que tenga una personalidad empática pero robusta para coordinar y gestionar el proceso de intervención. Brindará orientación y apoyo en la derivación, evaluación y gestión de los casos.

<u>Metodología </u>(Figura 5)

Los trabajadores con alguna condición musculoesquelética pueden optar voluntariamente a la gestión de casos:

1) Por sí mismos.
2) A propuesta de su supervisor.
3) A propuesta del médico del trabajo del Servicio de Prevención.

Los trabajadores con patología orgánica subyacente (*red flag*) quedan excluidos de este componente de intervención y se gestionan según la práctica médica estándar.

Un gestor de casos calificado (curso de Vocational Rehabilitation Case Management, Healthy Working Lives, University of Glasgow) asigna a los participantes a tres estratos de gestión y tratamiento, según su nivel de riesgo de síntomas musculoesqueléticos persistentes: bajo, medio o alto. Este perfil se obtiene en una entrevista telefónica mediante un cuestionario que incluye herramientas validadas para generar un perfil de riesgo que evalúa la presencia de dolor, comorbilidad, limitaciones para llevar a cabo actividades diarias, miedo, creencias y expectativas negativas en cuanto al pronóstico del dolor, a la presencia de ansiedad y otros trastornos del estado de ánimo [45-51] (Anexo 4).

- Los trabajadores asignados al grupo de bajo riesgo asisten a una sesión educativa sobre creencias relacionadas con la salud y el dolor.
- Los trabajadores asignados a los grupos de riesgo medio y alto reciben un tratamiento específico y personalizado que incluye la rehabilitación, fisioterapia y terapia cognitiva-conductual.

Se estudiará cada caso por separado y se adaptará el tratamiento específico a cada trabajador. Sin embargo, también como orientación se establece una pauta para determinar el cierre de los casos y la periodicidad de los seguimientos motivacionales (Anexo 5).

Figura 5. Algoritmo de la gestión de casos.

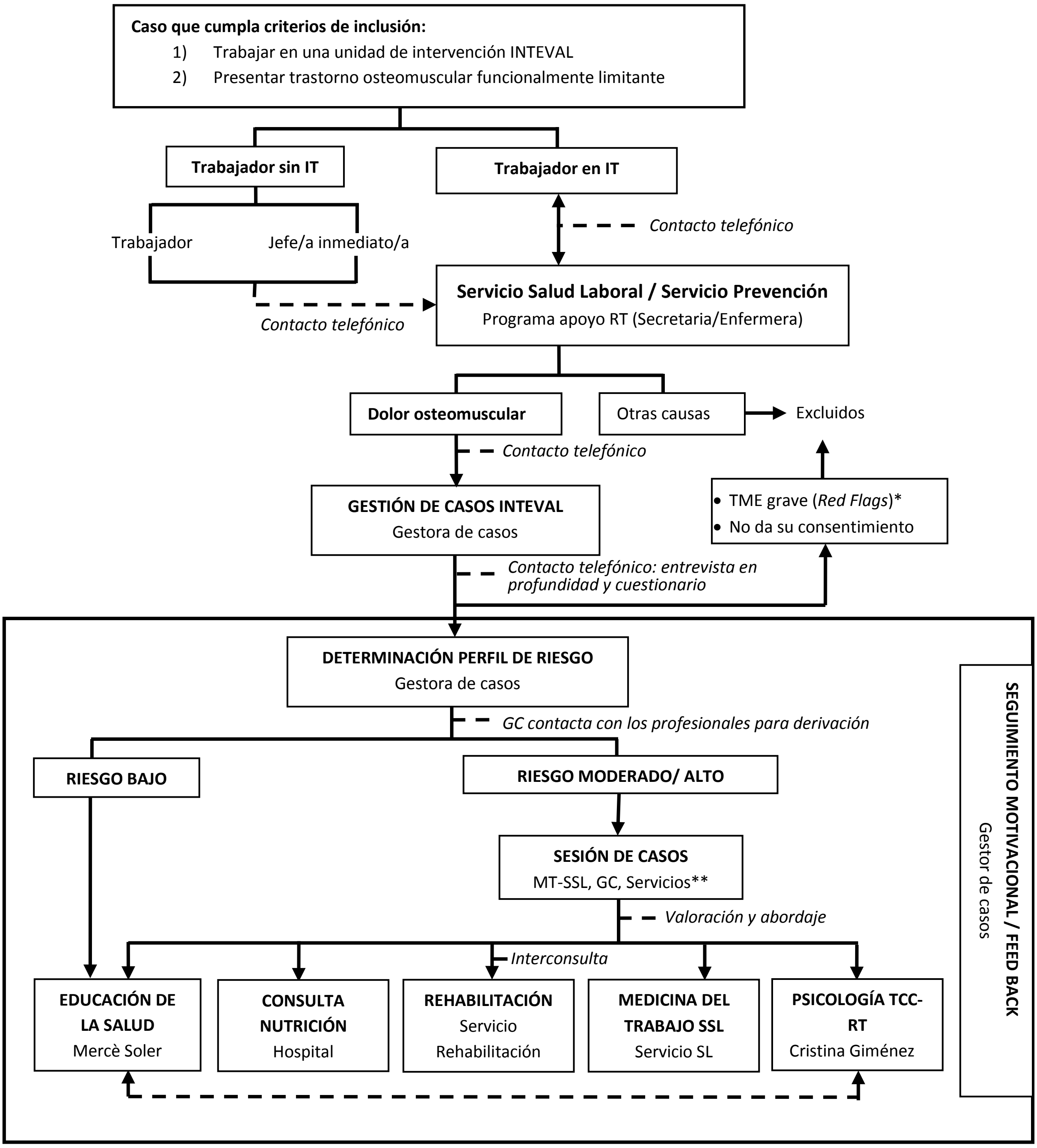

**Red Flags*: Presencia de señales de alerta (traumatismos severos o traumatismos leves en pacientes con osteoporosis, síntomas constitucionales: fiebre, pérdida de peso, malestar general), embarazo o puerperio, obesidad mórbida (IMC ≥ 40), intervención quirúrgica reciente (<3 meses), enfermedad neurodegenerativa, patología psiquiátrica descompensada, patología cardíaca o respiratoria descompensada, osteoporosis con fracturas vertebrales sintomáticas.

**Se derivará a los diferentes servicios en función de necesidades identificadas.

Paralelamente, se podrán presentar casos a la sesión clínica semanal con miembros del Servicio de Prevención para evaluar posibles necesidades específicas en el trabajo, como ajustes o mejoras en el trabajo para ayudar a los trabajadores a permanecer y/o volver pronto al trabajo.

El gestor de casos contactará telefónicamente con el trabajador de manera periódica para llevar a cabo un seguimiento motivacional, además de coordinar los servicios y planificar las sesiones.

- **Promoción de la salud**

Se creará un programa de promoción de la salud ofrecido a todas las enfermeras y auxiliares de enfermería de las unidades de intervención participantes y la aplicación será voluntaria y gratuita. Incluirá:

a) Formación de Mindfulness: definida como un enfoque de autorregulación para la reducción del estrés y la gestión emocional [29], y consistirá en un curso adaptado de 4 sesiones de 2 horas cada una en la formación de reducción del estrés (MBRS), el cual ha demostrado ser efectivo en trabajadores sanitarios [52].

b) Formación Nordic Walking: definida como una técnica de caminata que utiliza palos especialmente diseñados para implicar activamente la parte superior del cuerpo y los brazos con una amplia evidencia científica de sus beneficios sobre varios resultados de salud, incluidos el DME [36], que consistirá un programa de formación de 12 sesiones de 1,5 horas/sesión durante 12 semanas.

c) Alimentación saludable basada en la dieta mediterránea: uno de los modelos dietéticos más saludables que existen actualmente [37, 38] dividido en dos actividades principales:

    a. Sesión presencial de alimentación saludable: sesión realizada por un nutricionista/chef para fomentar la adherencia a la dieta mediterránea y hábitos alimentarios saludables. Tiempo estimado: 2-3h.

    b. Plataforma web: se compartirán recetas saludables de manera periódica (2 recetas por mes) supervisadas por un nutricionista y coordinado por el/la Champion. Además, se completará con consejos para un estilo de vida saludable y un foro de discusión.

Se organizarán charlas informativas in situ en las unidades para presentar cada una de estas actividades a los participantes, por unidades y turnos de trabajo. La participación en las actividades de promoción de la salud será considerada como formación y reconocidas como horas de formación acreditada por Formación Continuada del hospital, y contarán para la carrera profesional.

- **Integración de los componentes de la intervención inteval**

Todos los componentes de la intervención están integrados y requieren la coordinación de un/una *Champion* que actúa como líder y facilitador de la intervención, organizando y dirigiendo el trabajo que debe desarrollar el equipo del estudio.

Sus tareas incluyen:

- Dinamización de grupo.
- Comunicación entre el equipo de investigación y los participantes.
- Planificación de reuniones (sesiones informativas y reuniones de equipos de investigación).
- Organización de las actividades de promoción de la salud (la planificación del calendario).
- Recopilación y el procesamiento de datos.
- Redacción de informes.

- **Atención habitual de salud laboral**

Durante el período de intervención, el SSL de cada hospital participante continuará proporcionando las prácticas estándar de salud laboral tanto para las unidades de intervención como para las unidades de control. Estas prácticas incluyen:

a) Las evaluaciones de riesgos laborales.
b) La investigación de accidentes laborales.
c) La vigilancia de la salud.
d) La cesación del tabaquismo.
e) La formación e la información.
f) La evaluación de expertos en salud laboral en todos los niveles del hospital (directivos, supervisores, trabajadores).

g) El programa de apoyo habitual para el retorno al trabajo, principalmente a las intervenciones relacionadas con las adaptaciones del puesto de trabajo, el apoyo clínico y la gestión de la incapacidad permanente.

RECOGIDA DE DATOS

Los instrumentos para la recogida de datos se recogerán mediante tres fuentes:

a) Cuestionarios administrados a los articipantes.

b) Registros existentes en las empresas participantes.

c) Entrevistas cualtitativas.

Cuestionarios

Una parte de los datos se recogeran mediante cuestionarios estandarizados y validados.

Para la evaluación de la efectividad de la intervención, estos cuestionarios se administrarán en el momento inicial, seguimiento de seis y doce meses (ver apartado "Evaluación de la intervención", en formato papel, y el/la Champion del proyecto los procesará en el registro on-line.

Para asegurar un correcto registro se pautan unas indicaciones generales:

- Si alguna de las preguntas de una sola respuesta tiene más de una casilla marcada, se anula y se deben dejar las dos en blanco *(missing).*
- Utilizar siempre una coma (,) en lugar de un punto (.) Cuando se pongan decimales.
- Llenar las preguntas abiertas siempre con minúscula, incluyendo la primera letra.

Se seleccionará una muestra del 1% de cuestionarios y se comprobará su procesamiento para detectar posibles inconsistencias y/o identificar errores. Se revisarán todos los cuestionarios cuando se detecte un 10% o más de errores.

Los cuestionarios utilizados en la evaluación de proceso con relacióna la gestión de casos, serán administrados por vía telefónica.

Registros

Se dispondrá de los datos de los siguientes registros:

- Registro interno del/la Champion: almacenará los datos referentes a los indicadores de proceso, y los costes de la intervención.
- Los datos se extraerán principalmente de dos fuentes: el registro interno del/la Champion, y los registros del hospital (Servicio de Prevención, RRHH y departamento de contabilidad analítica).
- Registros del hospital: datos de la incapacidad temporal (SSL y RRHH), datos del coste de la enfermedad (Servicio de Prevención y Departamento de Contabilidad Analítica).

Entrevistas

Se desarrollarán dos grupos de entrevistas semiestructuradas: uno del equipo de investigación y el otro con un grupo de participantes del estudio de cada hospital, con la finalidad de aportar datos para la evaluación de proceso (ver evaluación de proceso).

EVALUACIÓN DE LA INTERVENCIÓN

La evaluación de la intervención se dividirá en:

- Evaluación de los resultados en salud
- Evaluación del proceso de implmentación de la intervención
- Evaluación económica

La Tabla 3 muestra los períodos de recogida de datos según los indicadores y tipo de evaluación; y la Tabla 4 presenta las herramientas que componen los cuestionarios INTEVAL.

Tabla 3. Evaluación, indicadores y períodos de recogida de datos.

Evaluación	Indicadores	T0	T1 (6 meses)	T2 (12 meses)	Período de intervención
Resultados en salud	Prevalencia de DME	X	X	X	
	Incidencia y duración IT por TME				X
	Capacidad funcional	X	X	X	
	Cultura preventiva	X	X	X	
Co-variables	Edad, sexo, país de nacimiento, puesto de trabajo, tipo de contrato, antigüedad en la empresa y el puesto	X	X	X	
Proceso	Reclutamiento				X
	Contexto			X	
	Alcance				X
	Dosis suministrada				X
	Dosis recibida				X
	Fidelidad				X
	Satisfacción			X	
	Rol personas clave (estrategia de implementación				X
Económica	Costes de la intervención				X
	Costes de la enfermedad				X
	Efectividad (prevalencia DME)	X	X	X	
	Calidad de vida (EQ-5D)	X	X	X	

- **Evaluación de la efectividad**

Las variables principales de resultado serán la prevalencia del dolor musculoesquelético y la incapacidad temporal asociada.

Como variables de resultado secundarias se tendrá en cuenta la capacidad funcional para trabajar i la cultura organizacional preventiva organizacional.

a) **Prevalencia del dolor musculoesquelético:** se medirá el DME mediante la adaptación española del cuestionario nórdico incluida en el método ERGOPAR [42]. Esta herramienta mide el dolor en cuello, hombros y espalda dorsal, espalda lumbar, codos, manos, piernas, rodillas y pies mediante la pregunta "¿Tiene molestias o

dolor en esta zona". Los datos se recogen en el momento basal, a los seis y los 12 meses.

b) **Incapacidad temporal:** los datos de los episodios y la duración de las incapacidades temporales a causa de una condición musculoesquelética se recogen los registros de RRHH y del SSL durante el período de estudio y hasta un año antes de la intervención.

c) **Variables de resultado secundarias**

   a. **Capacidad funcional para trabajar:** se recoge en el cuestionario basal, seis y doce meses, mediante la versión española del Work Role Functioning Questionnaire (WRFQ-SPV)) [53-55]. Esta herramienta es un cuestionario autoadministrado que mide dificultades percibidas a la hora de llevar a cabo su trabajo debido a problemas de salud [56] y consta de 27 ítems divididos en cinco sub-dominios: demandas de programación de trabajos, demandas de producción, demandas físicas, demandas mentales y demandas sociales. La puntuación de este cuestionario oscila entre 0 y 100, siendo la puntuación máxima 100 (teniendo el 100% de su capacidad funcional).

   b. **Cultura organizacional preventiva:** se mide a través del IWH Organizational Performance Metric (IWH-OPM) [57], cuestionario basado en la evidencia científica de ocho elementos que sirve para ayudar a las organizaciones a evaluar y mejorar su rendimiento de salud y seguridad y se mide en el cuestionario basal, a los seis y doce meses.

d) **Covariables**: En los cuestionarios utilizados y a partir de los registros rutinarios de las empresas se recogerá también, información sobre edad, sexo, país de nacimiento, puesto de trabajo, tipo de contrato, distribución del tiempo de trabajo y antigüedad en la empresa y en el puesto de los trabajadores en las unidades participantes.

Tabla 4. Cuestionarios INTEVAL: objetivos y períodos de recogida de datos

CUESTIONARIOS INTEVAL	OBJETIVO	Q basal	Q1 (6 meses)		Q2 (12 meses)	
			I	C	I	C
Nordic Questionnaire (método ERGOPAR), adaptado al ámbito sanitario	Recoger las variables sociodemográficas, evaluar los trastornos musculoesqueléticos mediante el dolor autopercibido, y conocer las posturas propias del trabajo, así como la carga del mismos	X				
Participación en las actividades	Conocer la participación en las actividades desarrolladas en el proyecto INTEVAL		X		X	
Pregunta mejora	Evaluar la mejora en movilizaciones, transferencias, y manipulación de cargas				X	X
Pregunta Servicio de Prevención	Evaluar el conocimiento de los participantes respecto al personal de prevención	X	X	X	X	X
IWH Organizational Performance Metric Questionnaire	Evaluar la cultura preventiva de los participantes	X	X	X	X	X
Satisfacción laboral	Conocer la satisfacción	X	X	X	X	X
Preguntas CoPsoQ-istas21 (versión corta)	Conocer el entorno psicosocial y productividad	X			X	X
Work Role Functioning Questionnaire (WRFQ-Sv)	Evaluar la capacidad funcional para trabajar	X	X	X	X	X
European Questionnaire five dimensions (EQ-5D-3L)	Evaluar la calidad de vida	X	X	X	X	X
Nordic Questionnaire (método ERGOPAR), adaptado al ámbito sanitario – pregunta abierta	Conocer la opinión y comentarios de los trabajadores	X	X	X	X	X
Cuestionario evaluación método ERGOPAR	Evaluar el método ERGOPAR				X	
Participación en la Gestión de casos	Conocer la participación en la gestión de casos				X	

- **Evaluación de proceso**

El proceso de intervención se evaluará mediante datos cuantitativos y cualitativos.

Evaluación cuantitativa

Se basará en indicadores para la evaluación de proceso [58, 59]: reclutamiento, contexto, alcance, dosis oferta, dosis recibida, fidelidad y satisfacción. También, añadiremos el rol de las personas clave (estrategia de implementación). Los datos cuantitativos de la evaluación de proceso se recogerán a través de cuestionarios y registros del/la Champion.

a) **Contexto**: se recopilará con los cuestionarios de los seis y doce meses de seguimiento con tres preguntas relacionadas con los aspectos que afectan a su carga de trabajo habitual (mejora de la movilización manual de los pacientes, ayudas técnicas y manejo de la carga).

b) **Reclutamiento**: se refiere a los procedimientos utilizados para abordar y atraer futuros participantes del programa, y se define como la proporción de posibles trabajadores que firmen el consentimiento informado para participar en el estudio, sobre el total de posibles participantes.
c) **Alcance**: se calculará como la proporción de personas que respondieron los cuestionarios (basales) de los que firmarán el consentimiento informado. Para cada componente de la intervención, el alcance se calculará como la proporción de personas que habrán participado en cada una de ellas.
d) **Dosis oferta**: se calculará como el número de horas de servicios ofrecidos.
e) **Dosis recibida**: según el grado en que los participantes han participado activamente en cada componente de la intervención.
f) **Fidelidad:** incluirá la fidelidad de la intervención y la adhesión de los participantes.
   a. La fidelidad de la intervención se calculará como la proporción entre las sesiones y/o actividades desarrolladas sobre el total de las planeadas.
   b. La adhesión de los participantes se calculará como la proporción entre su asistencia y la dosis oferta.
g) **Satisfacción**: se recogerá al final de cada componente de la intervención y en el cuestionario de seguimiento de 12 meses, a través de las preguntas "¿Has cumplido tus expectativas?", Y "en general, cuál es tu satisfacción? " una escala de 1 a 10, siendo la 10 la máxima satisfacción.

Evaluación cualitativa

Los datos cualitativos se utilizarán para identificar los puntos clave y las posibles mejoras, como indicador de estrategia de implementación y se recogerán mediante entrevistas semi-estructuradas.

Se desarrollarán dos grupos de entrevistas semiestructuradas: uno del equipo de investigación y el otro con un grupo de participantes del estudio de cada hospital. El objetivo de estos grupos será llevar a cabo la evaluación del proceso interno para detectar las limitaciones y barreras, a fin de realizar mejoras a nivel organizativo, así como ayudar a comprender algunos resultados.

Los puntos discutidos en las entrevistas serán:

a) Comunicación
b) Cuestionarios (formato, distribución, etc.),

c) Componentes de la intervención:
    a. Ergonomía participativa (reuniones, organización, medidas implementadas y difusión)
    b. Gestión de casos (difusión y formato y calidad del curso)
    c. Marcha nórdica (difusión y formato y calidad del curso)
    d. Mindfulness (difusión y formato y calidad del curso)
    e. Dieta saludable (página web en línea, actividad de la sesión del chef y lugar y horario de la sesión del chef).

- **Evaluación económica**

Se llevará a cabo un análisis de coste-utilidad y coste-efectividad desde las perspectivas del sistema de nacional de salud y de la sociedad; así como un análisis de coste-beneficio desde la perspectiva del hospital.

- **Los análisis** de coste-utilidad se llevarán a cabo para analizar los cambios en los años de vida ajustados por la calidad (AVAC), medidos por EQ-5D-3L [49], y los costes correspondientes para cada perspectiva. Además, el análisis coste-efectividad medirá la efectividad en términos de reducción del DME.
- **La perspectiva** del sistema sanitario nacional incluirá los costes directos de los servicios de salud pública españoles (costes directos de la enfermedad: visitas al médico de cabecera, especialistas, pruebas diagnósticas y medicamentos) y los costes de la intervención; la perspectiva social, incluirá todos estos costes y también la pérdida de producción (costes indirectos).
- **Los costes directos** se calcularán a partir de datos administrativos de los registros clínicos. Los costes indirectos se estimarán utilizando la aproximación del capital humano por incapacidad (registros de empresas).
- **Los costes de la intervención** (es decir, el tiempo de los expertos) se obtendrán los registros del estudio y se convertirán en costes unitarios según los convenios colectivos correspondientes.

ANÁLISIS ESTADÍSTICO

Al tratarse de un ensayo controlado aleatorizado por clústeres (ECAC), en el análisis se deben tener consideraciones especiales para el diseño de clúster. La mayoría de las inferencias se referirán a nivel de grupo, pero tal y como se recomienda [60], se obtendrán estimaciones ajustadas y no ajustadas por el agrupamiento.

Se realizarán análisis estadísticos adaptados para ensayos controlados aleatorizados con clústeres. Se realizarán análisis descriptivos de las características de los participantes y de las características referidas a los grupos de intervención y control a través de pruebas tradicionales, incluyendo prueba de t para variables continuas y normales; prueba de Wilconxon para variables no normales; prueba de McNemar o ji cuadrado para variables categóricas. Para las mediciones repetidas se aplicarán, según la tipología de las variables, las pruebas de ANOVA, Friedman o Q de Cochran. El procedimiento de ecuaciones de estimación generalizada (GEE) se utilizará para el análisis del DME, la capacidad funcional para trabajar y la cultura organizacional preventiva organizativa; comparando la diferencia del momento basal con el seguimiento a los 12 meses del grupo de intervención, respecto la diferencia del momento basal el seguimiento a los 12 meses del grupo de control. Los modelos serán ajustados por el diseño del clúster y por las posibles variables de confusión.

Se analizará la incidencia de incapacidad temporal mediante un modelo de regresión logística. Un modelo de riesgo proporcional de Cox estimará la relación de riesgo de volver a trabajar más bien después de la intervención en el grupo de intervención en comparación con el grupo control.

Los análisis estadísticos se llevarán a cabo con STATA 13 (StataCorp, 2013). Stata Statistical Software: versión 13. College Station, TX: StataCorp LP).

REQUISITOS ÉTICOS

- **Confidencialidad de datos**

  Todos los datos recopilados durante este estudio serán analizados, exclusivamente por el equipo investigador del Proyecto INTEVAL_Spain. La información será utilizada de forma anónima y será identificada siempre de forma numérica, evitando revelar cualquier tipo de información privada. El tratamiento de los datos recopilados se llevará a cabo de

manera agregada, nunca de manera individual. La empresa no tendrá acceso a la información recopilada, y esta nunca será utilizada para otro fin que no sea el Proyecto INTEVAL_Spain. Tanto los miembros de la Comisión como los profesionales que participan dando apoyo administrativo a la gestión de casos deben garantizar la confidencialidad de los datos de los profesionales incluidos en el proyecto.

El Centro de Investigación en Salud Laboral (CISAL), de la Universidad Pompeu Fabra (UPF), de Barcelona, se compromete a tratar toda la información con estricta confidencialidad, cumpliendo lo previsto en la Ley Orgánica 15/1999, de protección de datos, y demás legislación vigente en materia de protección de datos.

- **Voluntariedad**

La colaboración en el Proyecto INTEVAL_Spain es totalmente voluntaria. En caso de no querer colaborar en el proyecto, esto no perjudicará al trabajador en ningún aspecto laboral. Además, siguiendo la normativa sobre protección de datos (Ley Orgánica 15/1999 de Protección de Datos de Carácter Personal), en caso de querer anular sus datos en relación a este programa podrá dirigirse al equipo médico del servicio de Prevención.

- **Aprobación del comité ético de investigación clínica**

Previo a la implementación del programa, se ha obtenido el informe del Comité de Ética de Investigación Clínica del Par de Salud Mar, evaluando este proyecto (nº2014/5714/I), obteniendo su aprobación (Anexo 6).

FORTALEZAS Y LIMITACIONES

El ensayo controlado aleatorio (ECA) es el paradigma metodológico básico para la evaluación de intervenciones en salud. La aleatorización garantiza que la asignación de una unidad de trabajo en el grupo de intervención o de control sea exclusivamente debida al azar, evitando así efectos de confusión y sesgos de selección. La disponibilidad de un grupo de control permite distinguir entre asociaciones epidemiológicas y/o estadísticas y relaciones causa-efecto, algo fundamental en los estudios etiológicos. Asimismo, el diseño

prospectivo con al menos una medida antes de la intervención y varias medidas después permite estudiar los cambios en el tiempo.

Sin embargo, la implementación de ensayos controlados en los lugares de trabajo es mucho más complicada y difícil que en el ámbito clínico. Este es uno de los grandes retos en este tipo de estudios de intervención en salud laboral, para los que se ha requerido mayor adecuación de los siguientes aspectos [61]: (1) calidad de la intervención; (2) calidad del proceso de la intervención; (3) selección y tamaño de la muestra; (4) base teórica; (5) asignación aleatoria; (6) tiempo de seguimiento; (7) análisis estadísticos.

Todos estos elementos han sido cuidadosamente considerados en el diseño del presente estudio. Sin embargo, alteraciones en el proceso de implementación, problemas de participación y la contaminación de los grupos de control son potenciales amenazas.

BIBLIOGRAFÍA

1. Almodóvar A, Galiana ML, Hervás P, et al. VII Encuesta Nacional de Condiciones de Trabajo. Madrid: Instituto Nacional de Seguridad e Higiene en el Trabajo; 2011.
2. Holtermann A, Clausen T, Aust B, Mortensen OS, Andersen LL. Risk for low back pain from diferent frequencies, load mass and trunk postures of lifting and carrying among female healthcare workers. Int Arch Occup Environ Health. 2013;86–4:463–470.
3. National Research Council. Steering Committee for the Workshop on Work-Related Musculoskeletal Injuries. Work-Related Musculoskeletal Disorders: Report, Workshop Summary, and Workshop Papers. Washington: National Academy Press; 1999.
4. Engel G. The need for a new medical model: A challenge for biomedicine. Science 1977;196:129-36.
5. Pincus T, Kent P, Bronfort G, Loisel P, Pransky G, Hartvigsen J. Twenty-five years with the biopsychosocial model of low back pain - is it time to celebrate? A report from the twelfth international forum for primary care research on low back pain. Spine. 2013;38: 2118–23.
6. Marras WS. State-of-the-art research perspectives on musculoskeletal disorder causation and control: the need for an intergraded understanding of risk. 2004. J Elect Kines. 2004;14:1–5.
7. Malchaire J, Cock N, Vergrachat S. Review of the factors associated with musculoskeletal problems in epidemiological studies. Int Arch Occup Environ Health. 2001; 74: 79-90.
8. Luttman A, Jager M, Griefahn B, Caffier G, Liebers F, Steinberg U. Preventing musculoskeletal disorders in the workplace. Geneve: World Health Organization; 2003.
9. Devereux J, Rydstedt L, Nelly V, Weston P, Buckle P. The role of work stress and psychological factors in the development of musculoskeletal disorders. Norwich: Health and Safety Executive Books; 2004.
10. Hauke A, Flintrop J, Brun E, Rugulies R. The impact of work-related psychosocial stressors on the onset of musculoskeletal disorders in specific body regions: a review and meta-analysis of 54 longitudinal studies. Work & Stress. 2011; 25(3): 243-256
11. Zohar D. Safety climate in industrial organizations: theoretical and applied implications. J Appl Psicol. 1980;65(1);96–102.
12. Glendon AI, Stanton NA. Perspectives on safety culture. Safety Sci. 2000; 34:193–214.

13. Garcia AM, Boix P, Canosa C. Why do workers behave unsafely at work? Determinants of safe work practices in industrial workers. Occup Environ Med. 2004; 61:239–46.
14. Gershon RR, Stone PW, Zeltser M, Faucett J, MacDavitt K, Chou SS. Organizational climate and nurse health outcomes in the United States: a systematic review. Ind Health. 2007;45(5):622-36.
15. Gimeno D, Benavides FG, Benach J, Amick BC 3rd. Distribution of sickness absence in the European Union countries. Occup Environ Med. 2004;61(10):867-9.
16. Benavides FG. Ill health, social protection, labour relations, and sickness absence. Occup Environ Med. 2006; 63(4): 228–229.
17. Aaviksoo E, Kiivet RA. Sickness benefit cuts mainly affect blue-collar workers. Scand J Public Health. 2014;42(6):497-503.Driessen et al, 2010)
18. Chou R, Shekelle P. Will this patient develop persistent disabling low back pain? JAMA. 2010;303(13):1295-302.
19. Coggon D, Ntani G, Palmer KT, Felli VE, Harari R, Barrero LH, et al. Disabling musculoskeletal pain in working populations: is it the job, the person, or the culture?. Pain. 2013;154(6):856-63.
20. Vargas-Prada S, Martínez JM, Coggon D, Delclos G, Benavides FG, Serra C. Health beliefs, low mood, and somatizing tendency: contribution to incidence and persistence of musculoskeletal pain with and without reported disability. Scand J Work Environ Health. 2013;39:589–598.
21. Rasmussen CDN, Holtermann A, Mortensen OS, Søgaard K, Jørgensen MB. Prevention of low back pain and its consequences among nurses' aides in elderly care: a stepped-wedge multi-faceted cluster-randomized controlled trial. BMC Public Health. 2013;13:1088.
22. World Health Organization. Towards a Common Language for Functioning, Disability and Health - ICF. Geneva: WHO; 2002.
23. Smedley J, Harris EC, Cox V, Ntani G, Coggon D. Evaluation of a case management service to reduce sickness absence. Occup Med. 2013;63(2):89-95.
24. Demou E, Gibson I, Macdonald EB. Identification of the factors associated with outcomes in a Condition Management Programme. BMC Public Health. 2012;12:927.
25. Hoefsmit N, Houkes I, Nijhuis FJ. Intervention characteristics that facilitate return to work after sickness absence: a systematic literature review. J Occup Rehabil. 2012;22(4):462-77.

26. Westgaard RH, Winkel J. Ergonomic intervention research for improved musculoskeletal health: a critical review. Int J Ind Ergon. 1997; 20: 463–500.
27. Silverstein B, Clark R. Interventions to reduce work-related musculoskeletal disorders. J Elect Kines. 2004;14: 135–152.
28. Cole D, Rivilis I, Van Eerd D, Cullen K, Irvin E, Kramer D. Effectiveness of Participatory Ergonomic Interventions: A Systematic Review. Toronto, Ontario: Institute for Work & Health; 2005.
29. Tompa E, Dolinschi R, Natale J. Economic evaluation of a participatory ergonomics intervention in a textile plant. Appl Ergonomics. 2013; 44: 480-7.
30. Lallemand C. Contributions of participatory ergonomics to the improvement of safety culture in an industrial context. Work. 2012;41 Suppl 1:3284-90.
31. Brown JD, Mackay E, Demou J, Craig J, Frank J, Macdonald E. The EASY (Early Access to Support for You) sickness absence service: A four year evaluation of the impact on absenteeism. Scand J Work Environ Health. 2015; 41(2):204-215.
32. Case Management Society UK (CMSUK) [Internet]. Case management [accessed 23rd March 2015]. Available from: http://www.cmsuk.org/content.aspx?content=4.
33. Kamper SJ, Apeldoorn AT, Chiarotto A, Smeets RJ, Ostelo RW, Guzman J, et al. Multidisciplinary biopsychosocial rehabilitation for chronic low back pain. Cochrane Database Syst Rev. 2014;9:CD000963.
34. The National Institute of Occupational Safety and Health (NIOSH). NIOSH Total Worker Health. Webinar Series. https://www.cdc.gov/niosh/twh/. Accessed 21 Mar 2014.
35. 29. Zeller JM, Levin PF. Mindfulness interventions to reduce stress among nursing personnel: An occupational health perspective. Workplace Health Saf. 2013;61–2:85–89.
36. 30. Tschentscher M, Niederseer D, Niebauer J. Health benefits of Nordic Walking: a systematic review. Am J Prev Med. 2013;44–1:76–84.
37. 31. Martínez-González MA, Corella D, Salas-Salvadó J, Ros E, Covas MI, Fiol M et al. Cohort Profile: design and methods of the PREDIMED study. Int J Epidemiol. 2012;41–2:377–85.
38. 32. Saulle R, Semyonov L, La Torre G. Cost and cost-effectiveness of the Mediterranean diet: results of a systematic review. Nutrients. 2013;5–11:4566–4586.
39. Kramer DM, Wells RP, Carlan N, Aversa T, Bigelow PP, Dixon SM, et al. Did you have an impact? A theory-based method for planning and evaluating knowledge-transfer and

exchange activities in occupational health and safety. Int J Occup Saf Ergon. 2013;19(1):41-62

40. Best ML. An ecology of text: using text retrieval to study a life on the net. Artif Life. 1997;3–4:261–287.
41. Evanoff BA, Bohr PC, Wolf LD. Effects of a participatory ergonomics team among hospital orderlies. Am J Ind Med. 1999;35–4:358–365.
42. Gadea R, Sevilla M, García A. ERGOPAR 2.0. Un procedimiento de ergonomía participativa para la prevención de trastornos musculoesqueléticos de origen laboral. Madrid: Instituto Sindical de Trabajo, Ambiente y Salud (ISTAS); 2014.
43. García AM, Gadea R, Sevilla MJ, Ronda E. Validación de un cuestionario para identificar daños y exposición a riesgos ergonómicos en el trabajo. Rev Esp Salud Pública. 2011;85:331–340.
44. Haines H, Wilson JR. Development of a framework for participatory ergonomics. Norwich: Health and Safety Executive Books; 1998.
45. Hill JC, Dunn KM, Lewis M, Mullis R, Main CJ, Foster NE, Hay EM. A Primary Care Back Pain Screening Tool: Identifying Patient Subgroups for Initial Treatment. Arthritis Rheum. 2008;59–5:632–641.
46. Hill JC, Whitehurst DGT, Lewis M, Bryan S, Dunn KM, Foster NE, et al. Comparison of stratified primary care management for low Back pain with current best practice (STarT Back): a randomized controlled trial. Lancet. 2011;378:1560–1571.
47. Vargas-Prada S, Martínez JM, Coggon D, Delclos G, Benavides FG, Serra C. Health beliefs, low mood, and somatizing tendency: contribution to incidence and persistence of musculoskeletal pain with and without reported disability. Scand J Work Environ Health. 2013;39:589–598.
48. Sánchez-López P, Dresch V. The 12-Item General Health Questionnaire (GHQ-12): reliability, external validity and factor structure in the Spanish population. Psicothema. 2008;20–4:839–843.
49. Williams A. EQ-5D concepts and methods. Eds. Kind P, Brooks R, Rabin R. Dordrecht: Springer. 2005:1–17.
50. Schwarzer R, Jerusalem M. Generalized Self-Efficacy scale. In: Weinman J, Wright S, Johnston M, editors. Measures in health psychology: A user’s portfolio. Causal and control beliefs.Windsor: NFER-NELSON; 1995. pp. 35–37.

51. Bayliss EA, Ellis JL, Steiner JF. Seniors' self-reported multimorbidity captured biopsychosocial factors not incorporated into two other data-based morbidity measures. J Clin Epidemiol. 2009;62–5:550–557.
52. Bishop SR. What do we really know about mindfulness-based stress reduction? Psychosom Med. 2002;64:71–84.
53. Ramada JM, Serra C, Amick BC, III, Castano JR, Delclos GL. Cross-Cultural Adaptation of the Work Role Functioning Questionnaire to Spanish Spoken in Spain. J Occup Rehabil. 2013;23–4:566–575.
54. Ramada JM, Serra C, Amick BC, III, Abma FI, Castano JR, Pidemunt G, et al. Reliability and Validity of the Work Role Functioning Questionnaire (Spanish Version) J Occup Rehabil. 2014;24–4:640–649.
55. Ramada JM, Delclos GL, Amick BC, III, Abma FI, Pidemunt G, Castano JR, et al. Responsiveness of the Work Role Functioning Questionnaire (Spanish Version) in a General Working Population. J Occup Environ Med. 2014;56–2:189–194.
56. Amick BC, III, Lerner D, Rogers WH, Rooney T, Katz JN. A review of health-related work outcome measures and their uses, and recommended measures. Spine. 2000;25:3152–3160.
57. Organizational Indices Committee of the Occupational Health and Safety Council of Ontario . Benchmarking organizational leading indicators for the prevention and management of injuries and illnesses. Toronto: Institute for Work & Health; 2011.
58. Linnan L, Steckler A. Process evaluation for public health interventions and research. An overview. San Francisco: Jossey-bass; 2002.
59. Wierenga D, Engbers LH, van Empelen P, Hildebrandt VH. The design of a real-time formative evaluation of the implementation process of lifestyle interventions at two worksites using a 7-step strategy (BRAVO@work) BMC Public Health. 2012;12:619.
60. Campbell MK, Piaggio G, Elbourne DR, Altman DG. Consort 2010 statement: extension to cluster randomised trials. BMJ. 2012;345:e5661.
61. Kirstensen TS, Hannerz H, Hogh A, Borg V. The Copenhagen Psychosocial Questionnaire— a tool for the assessment and improvement of the psychosocial work environment. Scand J Work Environ Health 2005;31(6):438-449

ANEXOS

ANEXO 1: FICHA INFORMATIVA DEL PROYECTO INTEVAL_SPAIN Y CONSENTIMIENTO INFORMADO

FICHA INFORMATIVA DEL PROYECTO INTEVAL

El Centro de Investigación en Salud Laboral (CISAL), de la Universidad Pompeu Fabra (UPF), de Barcelona, está desarrollando el Proyecto INTEVAL en el Parc de Salut Mar (PSMAR), Barcelona.

OBJETIVO: El Proyecto INTEVAL consiste en una intervención multifactorial, compuesta de tres partes coordinadas entre sí:

1. Ergonomía Participativa, basada en el Método ERGOPAR, cuyo objetivo es identificar y mejorar las condiciones y situaciones de trabajo asociadas a la aparición de trastornos musculoesqueléticos (TME) en los trabajadores, y que incorpora la participación de los trabajadores a lo largo de dicho proceso.
2. Gestión de Casos, que consiste en el diagnóstico precoz y manejo personalizado de aquellos trabajadores afectados por TME discapacitantes, así como la posible adecuación y facilitación del retorno al trabajo, en condiciones seguras, de aquellos trabajadores en situación de Incapacidad Temporal (IT) a causa de un TME discapacitante.
3. Promoción de la salud que consiste en el ofrecimiento de varias actividades grupales para fomentar el ejercicio físico, la dieta mediterránea, el bienestar emocional

PARTICIPACIÓN:

Su participación en este proyecto es sumamente importante, ya que contribuirá a proporcionarnos datos muy valiosos, a partir de los cuales se podrán planificar y desarrollar cambios destinados a mejorar las condiciones actuales de trabajo de los trabajadores.

Durante el año de transcurso del estudio todos los trabajadores deberán cumplimentar varios cuestionarios sobre la percepción de su propia salud y sobre las exigencias y condiciones de su actual puesto de trabajo, capacidad funcional, calidad de vida y creencias de salud; respondiendo a preguntas sobre datos personales, datos sobre su trabajo actual, molestias físicas durante el tiempo de trabajo, si le impidieron o no realizar las acciones propias de su puesto laboral, su opinión sobre las causas y su opinión sobre su salud en general.

Durante la Ergonomía Participativa, todos los trabajadores tendrán oportunidad de participar en el proceso de identificación de problemas y soluciones liderados por los referentes de cada unidad. Éstos participarán a su vez en las reuniones de trabajo planificadas.

Durante la Gestión de Casos, podrá ser contactado telefónicamente para llevar un seguimiento de la evolución de su estado de salud, pudiendo ser derivado a alguno de los servicios previstos.

Durante las actividades de Promoción de la salud, los trabajadores que se inscriban adquirirán conocimientos sobre cómo tener cuidado de su propia salud (Marcha Nórdica, Mindfulness y alimentación saludable) y se comprometerán a asistir a todas las sesiones de las actividades a las que se haya inscrito.

CONFIDENCIALIDAD: Todos los datos recopilados durante este estudio serán analizados, exclusivamente por el equipo investigador del Proyecto INTEVAL. La información será utilizada de forma anónima y será identificada siempre de forma numérica, evitando revelar cualquier tipo de información privada. El tratamiento de los datos recopilados se llevará a cabo de manera agregada, nunca de manera individual. La empresa no tendrá acceso a la información recopilada, y ésta nunca será utilizada para otro fin que no sea el Proyecto INTEVAL.

El Centro de Investigación en Salud Laboral (CISAL), de la Universidad Pompeu Fabra (UPF), de Barcelona, se compromete a tratar toda la información con estricta confidencialidad, cumpliendo lo previsto en la Ley Orgánica 15/1999, de protección de datos, y demás legislación vigente en materia de protección de datos.

VOLUNTARIEDAD: Su colaboración en el Proyecto INTEVAL es totalmente voluntaria. En caso de no querer colaborar en el proyecto, esto no le perjudicará en ningún aspecto laboral. También puede, si cambia de opinión, rescindir su colaboración en el proyecto en cualquier momento, informando de su decisión al equipo de investigación a la mayor brevedad posible.

Si tiene alguna duda durante el desarrollo del proyecto, puede ponerse en contacto con Jose Mª Ramada, responsable del Proyecto INTEVAL a través del siguiente E-mail: proyectotinteval@gmail.com

Muchas gracias por su tiempo y su colaboración.

Firma responsable Proyecto INTEVAL:

CONSENTIMIENTO INFORMADO

TÍTULO DEL ESTUDIO:

Proyecto INTEVAL. Evaluación de una intervención multifactorial en el lugar de trabajo para la prevención y gestión de trastornos musculoesqueléticos en los trabajadores de empresas públicas.

DECLARACIÓN DE PARTICIPACIÓN VOLUNTARIA:

Declaro que he sido informado/a de la naturaleza y el propósito del Proyecto INTEVAL, de los datos que se me pide que proporcione y de las acciones que llevaré a cabo mediante mi participación. He recibido una explicación satisfactoria sobre los procedimientos del proyecto y su finalidad.

Comprendo que mi decisión de participar es voluntaria.

Presto mi consentimiento para la recopilación de datos y para la cumplimentación de diversos cuestionarios, así como para la participación en las acciones que se realizarán durante el desarrollo del proyecto.

Conozco mi derecho a retirar este consentimiento cuando lo desee, con la única obligación de informar mi decisión, a la mayor brevedad posible, al Equipo Investigador INTEVAL.

☐ Sí ACEPTO PARTICIPAR voluntariamente en este estudio, firme a continuación e indíquenos su nombre y apellidos, así como la fecha actual.

☐ NO ACEPTO PARTICIPAR voluntariamente en este estudio, firme a continuación e indíquenos su nombre y apellidos, así como la fecha actual.

Firma trabajador/a:	Firma de la persona que obtiene el consentimiento:
Nombre trabajador/a:	Dra.Consol Serra Pujadas
Fecha:	

ANEXO 2: CUESTIONARIO BASAL

CUESTIONARIO ERGOPAR

Apreciado/a compañero/a:

Tu participación completando este cuestionario es totalmente anónima y voluntaria, y nos será muy útil para identificar necesidades de mejora en este tema.

Es muy importante que respondas según tu propia opinión y con total sinceridad, pues sólo así podremos extraer conclusiones válidas. Por favor, completa toda la información que se solicita o señala con una X la casilla que mejor represente tu opinión al respecto.

Todo el tratamiento de los datos obtenidos lo realizará, de manera confidencial, el equipo investigador. De esta manera se garantiza por completo el anonimato de los trabajadores encuestados, y en ningún momento se relacionarán las respuestas con personas identificables.

Si tienes cualquier duda o comentario sobre este cuestionario, puedes contactar con Anna Amat.

Muchas gracias por tu participación,

Anna Amat

UPF – CiSAL

DATOS PERSONALES Y LABORALES

1. Eres: ☐ Hombre Mu[illegible] ☐

2. Edad: …… (años)

3. ¿Cuánto tiempo llevas trabajando en el PSMAR? ……… (años)

Si es menos de 1 año, marca esta casilla…… ☐

4. Antigüedad en el puesto de trabajo actual: ……… (años)

Si es menos de 1 año, marca esta casilla…… ☐

5. Tu horario es:

Turno de mañana………………………..	☐	Turno de tarde …………………………..	☐
Turno de noche…………………………..	☐	Otro (especificar)………………………..	☐

6. Habitualmente, ¿cuántas **horas al día** trabajas en este puesto?......... **horas/día**

7. Tu contrato es:

Indefinido…………………………………	☐	Eventual (temporal)…………………..	☐
D.I. Residente…………………………..	☐	Interino/a…………………………………	☐
Funcionario………………………………	☐	Otro (especificar)………………………..	☐

8. Indica la unidad en la que actualmente estás trabajando:

UGA (HOSPITAL DE LA ESPERANZA)………	☐	UH30 (HOSPITAL DEL MAR)………………	☐
LLEVANT 3 o 4 (CAEM)…………………..	☐	UCI (HOSPITAL DEL MAR)…………………	☐
UH04 (HOSPITAL DEL MAR)………………	☐	BLOQUE QUIRÚRGICO (HOSPITAL DEL MAR)	☐

9. Trabajas como:

Enfermero/a …………………………..	☐	Auxiliar de enfermería……………..	☐
Enfermero/a equipo complemento.............................	☐	Auxiliar de enfermería equipo complemento…………………………..	☐

10. Eres … (puedes señalar más de una opción, si es tu caso):

Trabajador/a …………………………..	☐	Responsable de Servicio/UH ……	☐
Delegado/a de prevención ………	☐	Miembro del Comité de Seguridad y Salud ……………………	☐

DAÑOS A LA SALUD DERIVADOS DEL TRABAJO

11. Para cada zona corporal indica si tienes MOLESTIA O DOLOR, su FRECUENCIA, si te ha IMPEDIDO REALIZAR TU TRABAJO ACTUAL y si esa molestia o dolor se han producido COMO CONSECUENCIA DE LAS TAREAS QUE REALIZAS EN TU ACTUAL PUESTO DE TRABAJO (AL QUE TE HAS REFERIDO EN LA PREGUNTA 9).

	**¿Tienes molestia o dolor en esta zona?**			**¿Con qué frecuencia?**			**¿Te ha impedido alguna vez realizar tu TRABAJO ACTUAL?**		**¿Se ha producido como consecuencia de tu actual PUESTO DE TRABAJO?**	
	No	Molestia	Dolor	No	A veces	Muchas veces	No	Sí	No	Sí
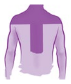 Cuello, hombro y/o espalda dorsal	☐	☐	☐	☐	☐	☐	☐	☐	☐	☐
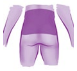 Espalda lumbar	☐	☐	☐	☐	☐	☐	☐	☐	☐	☐
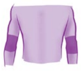 Codos	☐	☐	☐	☐	☐	☐	☐	☐	☐	☐
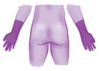 Manos y/o muñecas	☐	☐	☐	☐	☐	☐	☐	☐	☐	☐
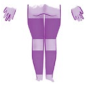 Piernas	☐	☐	☐	☐	☐	☐	☐	☐	☐	☐
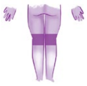 Rodillas	☐	☐	☐	☐	☐	☐	☐	☐	☐	☐
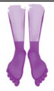 Pies	☐	☐	☐	☐	☐	☐	☐	☐	☐	☐

POSTURAS PROPIAS DEL TRABAJO

Contesta a cada pregunta SIEMPRE EN RELACIÓN CON UNA JORNADA HABITUAL EN TU ACTUAL PUESTO DE TRABAJO (AL QUE TE HAS REFERIDO EN LA PREGUNTA 9).

12. ¿Durante CUÁNTO TIEMPO tienes que trabajar adoptando o realizando estas posturas?

	**Nunca/Menos de 30 minutos**	**Entre 30 minutos y 2 horas**	**Entre 2 y 4 horas**	**Más de 4 horas**
Sentado (silla, taburete, apoyo lumbar, etc.)	☐	☐	☐	☐
De pie, sin andar apenas	☐	☐	☐	☐
Caminando	☐	☐	☐	☐
Caminando mientras subo o bajo niveles diferentes (peldaños, escalera, rampa, etc.)	☐	☐	☐	☐
De rodillas/en cuclillas	☐	☐	☐	☐

13. ¿Durante CUÁNTO TIEMPO tienes que trabajar adoptando o realizando estas posturas forzadas de CUELLO/CABEZA?

	**Nunca/ Menos de 30 minutos**	**Entre 30 minutos y 2 horas**	**Entre 2 y 4 horas**	**Más de 4 horas**	**Esta postura, ¿tienes que REPETIRLA cada pocos segundos, o MANTENERLA FIJA un tiempo?**	
					**La repito**	**La mantengo fija**
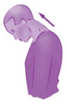 Inclinar el cuello / cabeza hacia delante	☐	☐	☐	☐	☐	☐
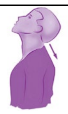 Inclinar el cuello /cabeza hacia atrás	☐	☐	☐	☐	☐	☐
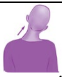 Inclinar el cuello /cabeza hacia un lado o ambos	☐	☐	☐	☐	☐	☐
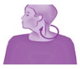 Girar el cuello / cabeza	☐	☐	☐	☐	☐	☐

14. ¿Durante CUÁNTO TIEMPO tienes que trabajar adoptando o realizando estas posturas forzadas de ESPALDA/TRONCO?

	**Nunca/ Menos de 30 minutos**	**Entre 30 minutos y 2 horas**	**Entre 2 y 4 horas**	**Más de 4 horas**	**Esta postura, ¿tienes que REPETIRLA cada pocos segundos, o MANTENERLA FIJA un tiempo?**	
					**La repito**	**La mantengo fija**
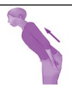 Inclinar la espalda/tronco hacia delante	☐	☐	☐	☐	☐	☐
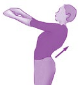 Inclinar la espalda/ tronco hacia atrás	☐	☐	☐	☐	☐	☐
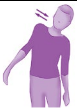 Inclinar la espalda/ tronco hacia un lado o ambos	☐	☐	☐	☐	☐	☐
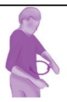 Girar la espalda/ tronco	☐	☐	☐	☐	☐	☐

15. ¿Durante CUÁNTO TIEMPO tienes que trabajar adoptando o realizando estas posturas de HOMBROS, MUÑECAS Y TOBILLOS/PIES?

	**Nunca/ Menos de 30 minutos**	**Entre 30 minutos y 2 horas**	**Entre 2 y 4 horas**	**Más de 4 horas**	**Esta postura, ¿tienes que REPETIRLA cada pocos segundos, o MANTENERLA FIJA un tiempo?**	
					**La repito**	**La mantengo fija**
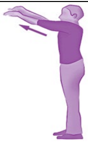 Las manos por encima de la cabeza o los codos por encima de los hombros	☐	☐	☐	☐	☐	☐
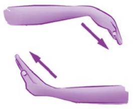 Una o ambas muñecas dobladas hacia arriba o hacia abajo, hacia los lados o giradas (giro de antebrazo)	☐	☐	☐	☐	☐	☐
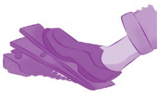 Ejerciendo presión con uno de los pies	☐	☐	☐	☐	☐	☐

16. ¿Durante CUÁNTO TIEMPO tienes que trabajar realizando estas acciones con las MANOS?

	**Nunca/Menos de 30 minutos**	**Entre 30 minutos y 2 horas**	**Entre 2 y 4 horas**	**Más de 4 horas**
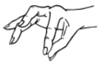 Sostener, presionar o levantar objetos o herramientas con los dedos en forma de pinza	☐ ☐	☐ ☐	☐ ☐	☐ ☐
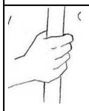 Empujar , agarrar o arrastrar con fuerza objetos o herramientas con las manos				
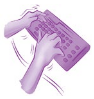 Utilizar de manera intensiva a los dedos (ordenador, botones, etc.)	☐	☐	☐	☐

17. MOVILIZACIÓN Y MANIPULACIÓN MANUAL DE PACIENTES. Responde en relación a cada una de las siguientes acciones.

**MOVILIZACIÓN MANUAL DE PACIENTES**			
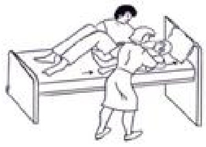	**¿Durante CUÁNTO TIEMPO tienes que trabajar realizando esta acción DURANTE UNA JORNADA HABITUAL en tu puesto de trabajo actual?**		
Nunca / Menos de 30 minutos……………………	☐	Entre 2 y 4 horas………………………………………	☐
Entre 30 minutos y 2 horas……………………….	☐	Más de 4 horas…………………………………………	☐
**La tipología de PACIENTES que manipulas DURANTE UNA JORNADA HABITUAL en tu puesto de trabajo actual es:**			
**Paciente colaborador** (puede valerse por sí solo):			
Nunca………………………………………………………	☐	Entre 6 y 10 pacientes………………………………	☐
Entre 1 y 5 pacientes…………………………………	☐	Más de 10 pacientes…………………………………	☐
**Paciente parcialmente colaborador** (debe ser parcialmente levantado):			
Nunca………………………………………………………	☐	Entre 6 y 10 pacientes………………………………	☐
Entre 1 y 5 pacientes…………………………………	☐	Más de 10 pacientes…………………………………	☐
**Paciente no colaborador** (debe ser completamente levantado, no se vale por sí solo):			
Nunca………………………………………………………	☐	Entre 6 y 10 pacientes………………………………	☐
Entre 1 y 5 pacientes…………………………………	☐	Más de 10 pacientes…………………………………	☐

Señala con qué FRECUENCIA movilizas pacientes de PESO ELEVADO:				
	Todos los días	Varias veces por semana	Ocasionalmente	Nunca
Pacientes entre 80-150 kg.	☐	☐	☐	☐
Pacientes de más de 150 kg.	☐	☐	☐	☐

Señala con qué FRECUENCIA movilizas PACIENTES:					
		Todos los días	Varias veces por semana	Ocasionalmente	Nunca
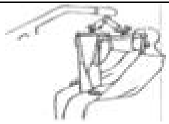	Con la ayuda de grúas	☐	☐	☐	☐
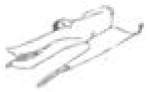	Con otros equipos de ayuda (transfers, discos, …)	☐	☐	☐	☐
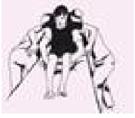	Con ayuda de otra persona	☐	☐	☐	☐

MOVILIZACIÓN MANUAL DE PACIENTES (EMPUJAR, ARRASTRAR, DESPLAZAR) SITUADOS EN ALGÚN EQUIPO DE DESPLAZAMIENTO (CAMA, CAMILLA, ETC…)			
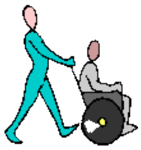 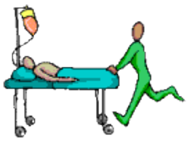		**¿Durante CUÁNTO TIEMPO tienes que trabajar realizando esta acción (empujar o arrastrar pacientes con ayuda de equipos de desplazamiento) DURANTE UNA JORNADA HABITUAL en tu puesto de trabajo actual?**	
Nunca / Menos de 30 minutos…………………………….	☐	Entre 2 y 4 horas………………………………………………..	☐
Entre 30 minutos y 2 horas………………………………	☐	Más de 4 horas…………………………………………………	☐

Señala con qué FRECUENCIA movilizas pacientes SITUADOS EN ALGÚN EQUIPO DE DESPLAZAMIENTO:				
	Todos los días	Varias veces por semana	Ocasionalmente	Nunca
Con ayuda de otra persona	☐	☐	☐	☐

18. MANIPULACIÓN MANUAL DE CARGAS DE MÁS DE 3 KG EN TOTAL (excluyendo manipulación de pacientes). Responde en relación a cada una de las siguientes tres acciones.

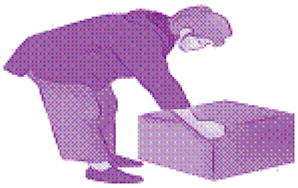		**COGER Y/O DEJAR MANUALMENTE objetos, herramientas o materiales de MÁS DE 3 KG.**	
**¿Durante CUÁNTO TIEMPO tienes que trabajar realizando esta acción?**			
Nunca / Menos de 30 minutos…………………………….	☐	Entre 2 y 4 horas………………………………………………..	☐
Entre 30 minutos y 2 horas………………………………	☐	Más de 4 horas…………………………………………………	☐
**RESPONDE SÓLO SI MANIPULAS CARGAS DE 3 O MÁS KILOS:**			
**Los PESOS que con mayor frecuencia coges y/o dejas son de:**			

Entre 3 y 5 kilos…………………………………………………	☐	Entre 15 y 25 kilos……………………………………………	☐
Entre 5 y 15 kilos……………………………………………	☐	Más de 25 kilos…………………………………………………	☐

Señala si habitualmente:

- ☐ Coges y/o dejas carga tú solo/a (sin ayuda de otra persona)
- ☐ Coges y/o dejas la carga por debajo de tus rodillas
- ☐ Coges y/o dejas la carga por encima de tus hombros
- ☐ Mantienes los brazos extendidos sin poder apoyar la carga en tu cuerpo
- ☐ Manipulas la carga con dificultad por no tener buen agarre (sin asa)
- ☐ Tienes que coger y/o dejar la carga cada pocos segundos

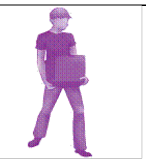

TRANSPORTAR MANUALMENTE objetos, herramientas o materiales de MÁS DE 3 KG.

¿Durante CUÁNTO TIEMPO tienes que trabajar realizando esta acción?

Nunca / Menos de 30 minutos………………………	☐	Entre 2 y 4 horas……………………………………………	☐
Entre 30 minutos y 2 horas……………………………	☐	Más de 4 horas…………………………………………………	☐

RESPONDE SÓLO SI MANIPULAS CARGAS DE 3 O MÁS KILOS:

Los PESOS que con mayor frecuencia coges y/o dejas son de:

Entre 3 y 5 kilos…………………………………………………	☐	Entre 15 y 25 kilos……………………………………………	☐
Entre 5 y 15 kilos……………………………………………	☐	Más de 25 kilos…………………………………………………	☐

Señala si habitualmente:

- ☐ Transportas la carga tú solo/a (sin la ayuda de otra persona)
- ☐ Transportas la carga con los brazos extendidos, sin apoyar la carga en tu cuerpo y sin doblar los codos
- ☐ Transportas la carga con dificultad, por no tener un buen agarre
- ☐ Caminas más de 10 metros transportando la carga
- ☐ Tienes que transportar la carga cada pocos segundos

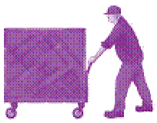

EMPUJAR Y/O ARRASTRAR MANUALMENTE o utilizando algún equipo (carro de enfermería, de ropa, de medicamentos…) de MÁS DE 3 KG.

¿Durante CUÁNTO TIEMPO tienes que trabajar realizando esta acción?

Nunca / Menos de 30 minutos………………………	☐	Entre 2 y 4 horas……………………………………………	☐
Entre 30 minutos y 2 horas……………………………	☐	Más de 4 horas…………………………………………………	☐

Señala si habitualmente:

- ☐ Tienes que hacer mucha fuerza para iniciar el empuje y/o arrastre

☐	Tienes que hacer mucha fuerza para desplazar la carga
☐	La zona donde tienes que poner las manos al empujar y/o arrastrar no es adecuada (muy alta, muy baja, difícil de agarrar, etc.)
☐	Tienes que caminar más de 10 metros empujando y/o arrastrando la carga
☐	Tienes que empujar y/o arrastrar la carga cada pocos segundos

19. En general, ¿cómo valorarías las **EXIGENCIAS FÍSICAS** EN TU ACTUAL PUESTO DE TRABAJO (AL QUE TE HAS REFERIDO EN LA PREGUNTA 9)?

Muy bajas………………………………………………………………………… ☐
Bajas………………………………………………………………………………… ☐
Moderadas……………………………………………………………………… ☐
Altas………………………………………………………………………………… ☐
Muy altas………………………………………………………………………… ☐

20. En relación a las POSTURAS Y ACCIONES PROPIAS EN TU ACTUAL PUESTO DE TRABAJO, **ordena del 1 al 5** las acciones que piensas que **afectan más a tu SALUD Y BIENESTAR** (siendo 1 la acción que afecte más, y 5 la que menos)

Movilizaciones y transferencias………………………………………….. ☐

Bipedestación prolongada…………………………………………………. ☐

Manipulación de cargas……………………………………………………… ☐

Factores psicosociales………………………………………………………… ☐

Espacio/instalaciones/factores ambientales………………………… ☐

Otros (especificar)……………………………………………………………….. ☐
…………………………………………………………………………………………
…………………………………………………………………………………………
…………………………………………………………………………………………
…………………………………………………………………………………………

¿Qué prácticas de seguridad y salud se realizan en el PSMAR?

Nos interesa conocer tu grado de acuerdo con cada una de las siguientes afirmaciones, señalando SÍ o NO dependiendo de si estás de acuerdo o no con la afirmación, o rodeando con un círculo la puntuación que mejor represente tu opinión al respecto. El valor "0" significa que estás totalmente en desacuerdo con la afirmación, y el valor "10" significa que estás totalmente de acuerdo. En caso de que haya alguna cuestión que desconozcas, señala la opción "No sé".

21. En mi empresa hay personas cuyo trabajo consiste en ocuparse de la prevención de riesgos laborales:

☐ SÍ ☐ NO ☐ No sé

22. Las personas que se encargan de la prevención de riesgos laborales en mi empresa tienen autoridad suficiente para llevar a cabo todas las mejoras y cambios necesarios para proteger la salud y seguridad de los trabajadores:

Totalmente en desacuerdo										Totalmente de acuerdo	
0	1	2	3	4	5	6	7	8	9	10	☐ No sé

23. En mi empresa se le da tanta importancia a la salud y seguridad de los trabajadores como al rendimiento y calidad en el trabajo:

Totalmente en desacuerdo										Totalmente de acuerdo	
0	1	2	3	4	5	6	7	8	9	10	☐ No sé

24. Valoro muy positivamente las actuaciones y mejoras en materia de seguridad y salud que puedan afectar a mi puesto de trabajo:

Totalmente en desacuerdo										Totalmente de acuerdo	
0	1	2	3	4	5	6	7	8	9	10	☐ No sé

25. En mi puesto de trabajo se realizan regularmente (al menos una vez cada año) evaluaciones y/o intervenciones para mejorar los aspectos de seguridad y salud en el trabajo:

☐ SÍ ☐ NO ☐ No sé

26. Dispongo de toda la información que necesito para trabajar de forma segura y sin perjudicar mi salud:

Totalmente en desacuerdo										Totalmente de acuerdo	
0	1	2	3	4	5	6	7	8	9	10	☐ No sé

27. En mi empresa se considera la opinión del trabajador respecto a las decisiones que afectan a su seguridad y salud:

Totalmente en desacuerdo										Totalmente de acuerdo	
0	1	2	3	4	5	6	7	8	9	10	☐ No sé

28. Obtengo un reconocimiento positivo por parte de mis encargados y superiores si realizo mis tareas evitando la exposición a riesgos laborales, protegiendo mi salud y/o la de mis compañeros:

Totalmente en desacuerdo										Totalmente de acuerdo	
0	1	2	3	4	5	6	7	8	9	10	☐ No sé

29. Dispongo de las herramientas y/o equipos necesarios para trabajar de manera segura y sin perjudicar mi salud:

Totalmente en desacuerdo										Totalmente de acuerdo	
0	1	2	3	4	5	6	7	8	9	10	☐ No sé

Desempeño y satisfacción laboral en el PSMAR

Las siguientes preguntas son acerca de la frecuencia con la que experimentaste determinadas situaciones en relación con la ejecución de tu trabajo en el PSMAR durante las últimas 4 semanas (28 días).

30. Rodea con un círculo la puntuación que mejor refleje tu experiencia en ese periodo.

	**Todo el tiempo**	**La mayor parte del tiempo**	**A veces**	**Casi nunca**	**Nunca**
**a.** ¿Con qué frecuencia tuviste **molestias o problemas de salud** que limitaron la cantidad o la calidad del trabajo que podías hacer?	1	2	3	4	5
**b.** ¿Con qué frecuencia tu rendimiento en el trabajo fue **más elevado** que el de la mayoría de los trabajadores en tu sección o puesto de trabajo?	1	2	3	4	5
**c.** ¿Con qué frecuencia tu rendimiento en el trabajo fue **más bajo** que el de la mayoría de los trabajadores en tu sección o puesto de trabajo?	1	2	3	4	5
**d.** ¿Con qué frecuencia **no trabajaste** en momentos en los que se suponía que debías estar trabajando?	1	2	3	4	5
**e.** ¿Con qué frecuencia te percataste de que no estabas trabajando tan **cuidadosamente** como deberías?	1	2	3	4	5
**f.** ¿Con qué frecuencia la **calidad** de tu trabajo fue inferior a lo que debería haber sido?	1	2	3	4	5

**g.** ¿Con qué frecuencia **no te concentraste** lo suficiente en tu trabajo?	1	2	3	4	5

Responde a las siguientes preguntas relacionadas con tu satisfacción en tu puesto de trabajo actual. Rodea con un círculo la puntuación que mejor represente tu opinión al respecto. El valor "0" significa que no estás nada satisfecho y el valor "6" que estás totalmente satisfecho.

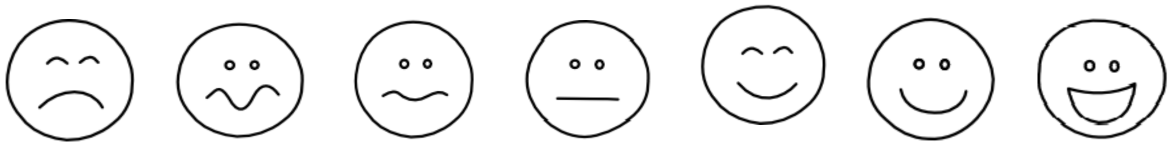

**Totalmente insatisfecho**	**Muy poco satisfecho**	**Poco satisfecho**	**Neutral**	**Satisfecho**	**Muy satisfecho**	**Totalmente satisfecho**
0	1	2	3	4	5	6

31. En relación con tu actual puesto de trabajo en el PSMAR, ¿cómo estás de satisfecha/o con

	**Totalmente insatisfecho**	**Muy poco satisfecho**	**Poco satisfecho**	**Neutral**	**Satisfecho**	**Muy satisfecho**	**Totalmente satisfecho**
**a.** … los compañeros en tu puesto y/o sección de trabajo?	0	1	2	3	4	5	6
**b.** … las tareas que debes realizar en tu puesto de trabajo?	0	1	2	3	4	5	6
**c.** … las condiciones de trabajo en tu puesto?	0	1	2	3	4	5	6
**d.** … tu superior inmediato?	0	1	2	3	4	5	6
**e.** …tus actuales condiciones de contratación/empleo?	0	1	2	3	4	5	6
**f.** … los materiales y equipos disponibles para realizar tu trabajo?	0	1	2	3	4	5	6

**g.** … tu trabajo en general?	0	1	2	3	4	5	6

32. Responde a las siguientes preguntas relacionadas con tu puesto de trabajo actual. Rodea con un círculo la puntuación que mejor represente tu opinión al respecto.

	**Todo el tiempo**	**La mayor parte del tiempo**	**A veces**	**Casi nunca**	**Nunca**
**a.** ¿Tienes que trabajar muy rápido?	1	2	3	4	5
**b.** ¿Tienes que trabajar intensamente? (mucha cantidad de trabajo en poco tiempo)	1	2	3	4	5
**c.** ¿Tu trabajo, en general, te exige demasiado?	1	2	3	4	5
**d.** ¿Tu trabajo permite que aprendas cosas nuevas?	1	2	3	4	5
**e.** ¿Tu trabajo exige que muestres iniciativa?	1	2	3	4	5
**f.** ¿Puedes elegir cómo hacer tu trabajo?	1	2	3	4	5
**g.** ¿Puedes elegir qué hacer en tu trabajo?	1	2	3	4	5
**h.** ¿Recibes ayuda y apoyo de tus compañeros de trabajo?	1	2	3	4	5
**i.** ¿Recibes ayuda y apoyo de tu superior inmediato/a?	1	2	3	4	5

TRABAJO Y SALUD

En las siguientes preguntas le pedimos que nos indique, para las **ÚLTIMAS CUATRO SEMANAS**, la cantidad de tiempo en que tuvo dificultad para realizar ciertos aspectos de su trabajo. Marque la casilla **"NO aplicable a mi trabajo"** sólo en caso de que la pregunta se refiera a algo que no es parte de su trabajo.

33. En las ÚLTIMAS CUATRO SEMANAS, ¿durante cuánto tiempo de su trabajo le fue difícil realizar las siguientes actividades por motivos de su salud física o problemas emocionales?

	**Fue difícil todo el tiempo (100%)**	**Fue difícil la mayor parte del tiempo**	**Fue difícil la mitad del tiempo (50%)**	**Fue difícil una parte del tiempo**	**Nunca fue difícil (0%)**	**NO aplicable a mi trabajo**
**a.** Trabajar el número de horas requeridas	☐	☐	☐	☐	☐	☐
**b.** Empezar la jornada de trabajo con facilidad	☐	☐	☐	☐	☐	☐
**c.** Ponerse a trabajar nada más llegar al trabajo	☐	☐	☐	☐	☐	☐
**d.** Hacer su trabajo sin parar a hacer descansos adicionales	☐	☐	☐	☐	☐	☐
**e.** Ajustarse a una rutina u horario	☐	☐	☐	☐	☐	☐
**f.** Manejar su carga de trabajo	☐	☐	☐	☐	☐	☐
**g.** Trabajar lo suficientemente rápido	☐	☐	☐	☐	☐	☐
**h.** Acabar el trabajo a tiempo	☐	☐	☐	☐	☐	☐

**i.** Hacer su trabajo sin cometer errores	▢	▢	▢	▢	▢	▢
**j.** Satisfacer a las personas que evalúan su trabajo	▢	▢	▢	▢	▢	▢
	**Fue difícil todo el tiempo (100%)**	**Fue difícil la mayor parte del tiempo**	**Fue difícil la mitad del tiempo (50%)**	**Fue difícil una parte del tiempo**	**Nunca fue difícil (0%)**	**NO aplicable a mi trabajo**
**k.** Tener sensación de trabajo bien hecho	▢	▢	▢	▢	▢	▢
**l.** Sentir que ha hecho lo que es capaz de hacer	▢	▢	▢	▢	▢	▢
**m.** Caminar o desplazarse a distintos lugares de trabajo	▢	▢	▢	▢	▢	▢
**n.** Levantar, cargar o mover objetos de más de 5 kg de peso, en el trabajo	▢	▢	▢	▢	▢	▢
**ñ.** Permanecer sentado, de pie o en una misma posición durante más de 15 minutos, mientras se trabaja	▢	▢	▢	▢	▢	▢
**o.** Repetir los mismos movimientos una y otra vez mientras trabaja	▢	▢	▢	▢	▢	▢
**p.** Doblarse, girarse o alcanzar un objeto mientras trabaja	▢	▢	▢	▢	▢	▢
**q.** Usar equipos o herramientas de mano	▢	▢	▢	▢	▢	▢
**r.** Mantener la mente en su trabajo	▢	▢	▢	▢	▢	▢

**s.** Pensar con claridad mientras trabaja	☐	☐	☐	☐	☐	☐
**t.** Hacer el trabajo con cuidado	☐	☐	☐	☐	☐	☐
**u.** Concentrarse en su trabajo	☐	☐	☐	☐	☐	☐
**v.** Trabajar sin perder el hilo (de las ideas)	☐	☐	☐	☐	☐	☐
	**Fue difícil todo el tiempo (100%)**	**Fue difícil la mayor parte del tiempo**	**Fue difícil la mitad del tiempo (50%)**	**Fue difícil una parte del tiempo**	**Nunca fue difícil (0%)**	**NO aplicable a mi trabajo**
**w.** Leer o usar los ojos con facilidad mientras trabaja	☐	☐	☐	☐	☐	☐
**x.** Hablar con la gente cara a cara, en reuniones o por teléfono	☐	☐	☐	☐	☐	☐
**y.** Controlar su genio delante de otras personas mientras trabaja	☐	☐	☐	☐	☐	☐
**z.** Ayudar a otras personas a acabar el trabajo	☐	☐	☐	☐	☐	☐

34. Seleccione la afirmación en cada sección que describa mejor su estado de salud en el día de hoy:

**a.**	**Movilidad**				
No tengo problemas para caminar	☐	Tengo algunos problemas para caminar	☐	Tengo que estar en la cama	☐

**b.**	**Cuidado personal**				
No tengo problemas con el cuidado personal	☐	Tengo algunos problemas para lavarme o vestirme solo	☐	Soy incapaz de lavarme o vestirme solo	☐

c.	**Actividades de <u>Todos los Días</u>** (ej, trabajar, estudiar, hacer tareas domésticas, actividades familiares o realizadas durante el tiempo libre)				
No tengo problemas para realizar mis actividades de todos los días	☐	Tengo algunos problemas para realizar mis actividades de todos los días	☐	Son incapaz de realizar mis actividades de todos los días	☐

d.	**Dolor/Malestar**				
No tengo dolor ni malestar	☐	Tengo moderado dolor o malestar	☐	Tengo mucho dolor o malestar	☐

e.	**Ansiedad/Depresión**				
No estoy ansioso/a ni deprimido/a	☐	Estoy moderadamente ansioso/a o deprimido/a	☐	Estoy muy ansioso/a o deprimido/a	☐

Para ayudar a la gente a describir lo bueno o lo malo que es su estado de salud, hemos dispuesto una escala parecida a un termómetro en el cual se marca con un 100 el mejor estado de salud que pueda imaginarse, y con un 0 el peor estado de salud que pueda imaginarse.

35. Por favor, de 0 a 100, ¿cómo puntuaría **su estado de salud hoy?**

Peor estado de salud imaginable 0 — 10 — 20 — 30 — 40 — 50 — 60 — 70 — 80 — 90 — 100 Mejor estado de salud

36. Aquí están algunas cosas que otros pacientes nos han dicho sobre su dolor. Por favor, para cada afirmación marque una casilla del 0 al 6 para indicar hasta qué punto las actividades físicas tales como inclinarse, levantar peso, caminar o conducir afectan o afectarían a su dolor de espalda

		En total desacuerdo		Ni en acuerdo ni desacuerdo			Totalmente de acuerdo	
		0	1	2	3	4	5	6
2	La actividad física hace que mi dolor empeore	☐	☐	☐	☐	☐	☐	☐
3	La actividad física podría dañar mi espalda	☐	☐	☐	☐	☐	☐	☐
4	No debería hacer las actividades físicas que empeoran mi dolor, ni las que podrían empeorarlo	☐	☐	☐	☐	☐	☐	☐
5	No puedo realizar las actividades físicas que empeoran mi dolor, ni las que podrían empeorarlo	☐	☐	☐	☐	☐	☐	☐

Las siguientes afirmaciones se refieren a cómo su trabajo normal afecta o afectaría a su dolor de espalda.

		En total desacuerdo		Ni en acuerdo ni desacuerdo			Totalmente de acuerdo	
		0	1	2	3	4	5	6
6	Mi dolor se debe a mi trabajo, o a un accidente en el trabajo	☐	☐	☐	☐	☐	☐	☐
7	Mi trabajo agravó mi dolor	☐	☐	☐	☐	☐	☐	☐
8	Mi trabajo es demasiado pesado para mí	☐	☐	☐	☐	☐	☐	☐
9	Mi trabajo empeora mi dolor, o podría empeorarlo	☐	☐	☐	☐	☐	☐	☐
10	Mi trabajo puede dañar mi espalda	☐	☐	☐	☐	☐	☐	☐
11	Con mi dolor actual no debería hacer mi trabajo normal	☐	☐	☐	☐	☐	☐	☐
12	No creo que pueda regresar a mi trabajo habitual en los próximos 3 meses	☐	☐	☐	☐	☐	☐	☐

37. Indica cualquier otra CUESTIÓN, COMENTARIO U OBSERVACIÓN que consideres de interés en relación con los temas tratados en este cuestionario:

MUCHAS GRACIAS POR TU COLABORACIÓN

ANEXO 3: PROCEDIMIENTO PARA LA APLICACIÓN DEL MÉTODO ERGOPAR

L'aplicació del mètode ERGOPAR té com a objectiu final la proposta i implementació de mesures de millora de les condicions de treball ergonòmiques de la/es unitat/s seleccionada/es a cada centre. Requereix d'una preparació, tant a nivell de grup com institucional, una infraestructura i condicions per a la seva implementació, unes accions i una visibilitat franca que és clau per a que els professionals de la/es unitat/es i la institució sigui conscient de les mesures implementades i que revertirà en una satisfacció a tots nivells.

En aquest document es detallen totes aquestes accions de preparació, implementació, seguiment i comunicació (taula 1), així com s'annexen els models de documents i documentació necessària en tot el procés (Documents).

Documents

3.1 Model d'Acord del Comitè de Seguretat i Salut del (nom institució) per a l'aplicació del mètode ERGOPAR.

3.2 Curs "Ergonomia en el ámbito sanitario y método ERGOPAR".

3.3 Document "Ergonomia laboral.doc"

3.4 Full de registre de problemes.

3.5 Rol i participació dels/de les referents de les unitats/clústers

3.6 Full de registre de solucions.

3.7 Model Informe propostes mesures de millora

3.8 Taula de planificació

Taula 1. Accions, responsables i contingut/guió de l'aplicació del mètode ERGOPAR

FASE	ACCIÓ	Responsable/s	Contingut/guió	Documentació/Observacions
**PREPARACIÓ**	1. Acord Comitè Seguretat i Salut (CSS)	Cap servei de salut laboral/servei de prevenció (SSL/SP), President CSS	Tot els membres del CSS (representants empresa i delegats de prevenció) han de signar el l'acord/compromís de suport a l'ERGOPAR de la/es unitat/s seleccionada/es (model annex 1).	Reunió més propera del Comitè de Seguretat i Salut. Idealment ha d'estar signat abans d'iniciar el procés ERGOPAR. Cal haver prèviament acordat prèviament amb la Direcció l'aplicació del mètode EROPAR fins al final.
	2. Constitució del grup ERGO	Cap SSL/SP	El grup està format per:<br>• Comandament/s de la unitat<br>• Referents: treballadors de l'equip de la unitat a nivell dels 4 torns (matí, tarda, nit A, nit B). Habitualment proposats pel cap de la unitat. Han de ser líders del seu equip unitat-torn<br>• Expert SSL: ergònom/a<br>• Champion	Crear grup WhatsApp: referents, comandaments, ergònom, champion, cap SSL/SP (administrador).
	3. Acreditació i reconeixement tasca referents	Formació continuada/Cap SSL/SP	Curs de formació, 20 hores de formació continuada acreditada per carrera professional. (annex 2)	Enviar proposta curs a Formació continuada.
	4. Avaluació de riscos	Tècnic de prevenció assignat a la unitat	Actualització de l'avaluació de riscos laborals de la unitat.	Es lliura presencialment al comandament de la unitat, junt amb una explicació sobre el contingut de l'avaluació.
**IMPLEMENTACIÓ** (cont.)	5. Reunió grup ERGO 1	Ergònom, Champion	• Formació en ergonomia laboral (conceptes d'ergonomia, manipulació manual de càrregues i postures forçades) i participativa (mètode ERGOPAR)<br>• Avaluació de riscos<br>• Resultats qüestionaris basals<br>• Explicació del full de registre de problemes per consensuar amb els cercles de prevenció de cadascun del torns i que caldrà fer entrega complimentat pels participants del grup en la següent reunió.	Full de signatures per formació continuada (tots signen). Cada institució disposa del seu model<br>Documentació que s'entrega **1 setmana ABANS** de la reunió:<br>• Document "Ergonomia laboral.doc" Annex 3<br>• Full de registre de problemes. Annex 4<br>• Consentiment informat sobre la tasca de referent als Referents. Annex 5

FASE	ACCIÓ	Responsable/s	Contingut/guió	Documentació/Observacions
IMPLEMENTACIÓ	6. Cercles de prevenció	Referents	Discussió de cada referent amb tot el seu equip de la unitat-torn sobre els problemes ergonòmics de la unitat, en reunions informals a la mateixa unitat.	Emplenar el full de registre de problemes
	7. Reunió grup ERGO 2	Ergònom, Champion	Posta en comú dels problemes identificats a cadascun dels torns i elaboració del llistat de problemes.	Full de signatures per formació continuada (tots signen). Cada institució disposa del seu model<br>S'entrega el full de registre de les possible solucions Annex 6
	8. Llistat de problemes	Champion, Ergònom	Elaboració del document de problemes	S'envia als referents la versió final
	9. Cercles de prevenció	Referents	Discussió en reunions informals amb equip unitat-torn les possibles solucions als problemes identificats	Emplenar el full de registre de solucions
	10. Reunió grup ERGO 3	Ergònom, Champion	Posta en comú de les solucions proposades a cadascun dels torns i elaboració del llistat de solucions, i priorització	Full de signatures per formació continuada (tots signen). Cada institució disposa del seu model
	11. Document final de problemes, prioritats i solucions	Champion, Ergònom	Elaboració del document final "INFORME PROPUESTAS MEDIDAS DE MEJORA" (model annex 7)	S'envia la versió final a tot el grup ERGO. Pot haver-hi algunes rondes de comentaris fins a la versió final.
	12. Presentació del document final de problemes, prioritats i solucions	Cap SSL/SP	Presentació del document final "INFORME PROPUESTAS MEDIDAS DE MEJORA" al Comitè de Seguretat i Salut. Afegir l'ERGOPAR a l'ordre del dia permanent del Comitè.	
**PLANIFICACIÓ, IMPLEMENTACIÓ I SEGUIMENT DE SOLUCIONS** (cont.)	13. Grup operatiu	Cap SSL/SP	• Funció: planificar i implementar les mesures proposades (solucions) pel grup ERGO.<br>• Constitució: Cap SSL/SP, Cap Unitat, Ergònom, Cap Infermeria (segons hospital), Cap de serveis generals/hoteleria i altres, segons necessitats.<br>• Reunions periòdiques: habitualment mensuals, taula de planificació	Documentació:<br>• Taula de planificació (model annex 8)<br>• Registre del cost de les mesures implementades (pot estar inclòs a la taula de planificació)

FASE	ACCIÓ	Responsable/s	Contingut/guió	Documentació/Observacions
**PLANIFICACIÓ, IMPLEMENTACIÓ I SEGUIMENT DE SOLUCIONS**	14. Informe final	Champion/Cap SSL/SP, grup ERGO	• Informe final de la implementació de mesures/solucions que s'elabora al final de la intervenció INTEVAL_Spain. Aquest informe ha de ser compartit i validat pel grup ERGO de la unitat.<br>• Presentació de l'informe final al CSS i a la Direcció, especialment la Direcció d'Infermeria.	Document: Informe **final** de la implementació de mesures/solucions (ERGOPAR INTEVAL_Spain).
	15. Seguiment de la implementació de mesures/solucions fins al final i informe definitiu	Cap SSL/SP	El compromís de l'ERGOPAR, va més enllà del projecte INTEVAL_Spain i per tant cal seguir la planificació i implementació de mesures/solucions fins a esgotar la llista. Al final de tot caldrà realitzar l'informe definitiu, que serà igualment presentat al CSS i la Direcció.	Document: Informe **definitiu** de la implementació de mesures/solucions (ERGOPAR).
**COMUNICACIÓ**	16. Comunicació la incorporació de mesures/solucions proposades pel grup ERGO	Champion	Informar periòdicament sobre l'estat actual de la implementació de les mesures de millora i cada vegada que s'implementi una solució, i feed-back dels referents i equip unitat:<br>• WhatsApp des de la coordinació (champion) als referents, i d'aquests a l'equip unitat-torn.<br>• Informació escrita (rètol) a l'espai reservat a INTEVAL_Spain del plafó/suro de la unitat.	

MODEL D'ACORD DEL COMITÈ DE SEGURETAT I SALUT DEL (NOM INSTITUCIÓ) PER A L'APLICACIÓ DEL MÈTODE ERGOPAR.

Data de l'acord:

Assistents

Membres del Comitè Seguretat i Salut:

**Representants empresa del *[nom institució]*:**	
**Delegats/des de Prevenció del *[nom institució]*:**	

Propòsit de l'acord

El propòsit de l'acord és aplicar el Mètode ERGOPAR al *[nom institució]*. El Mètode ERGOPAR permet, mitjançant un procediment participatiu, identificar problemes de naturalesa ergonòmica en l'empresa, prioritzar-los i proposar mesures preventives per a la seva correcció.

Agents implicats en l'acord

La direcció de l'empresa i els seus representants, i la representació legal dels treballadors, al Comitè de Seguretat i Salut.

Objectiu

Eliminar o reduir els trastorns musculoesquelètics d'origen laboral que poguessin afectar la salut dels treballadors de l'empresa mitjançant l'aplicació de mesures preventives específiques.

Àmbit d'intervenció

S'acorda la constitució de 3 unitats assistencials del *[nom institució]*, en les quals s'intervindrà de forma progressiva, amb un disseny abans - després.

Les unitats plantejades són: *[nom de la/es unitat/es intervenció]*. Cadascuna d'elles amb 36, 24 i 20. En total són 80 llocs de treball.

Les raons que justifiquen l'aplicabilitat del mètode en aquests llocs de treballs són diverses:

- [Exemple: UGA : detectat el risc de manipulació manual de càrregues (MMC), com a conseqüència de l'assistència a pacients complexes i amb alta dependència física i psíquica.]

Participació

A més de la implicació dels components del Grup Ergo, el Mètode ERGOPAR requereix participació a tots els nivells de l'empresa. L'aplicació del mètode requereix emplenar un qüestionari, entrevistes, observació de llocs de treball, reunions informatives i reunions de treball amb treballadors.

Cronograma

Acció	Unitat	Data prevista
1. Entrega i recollida de qüestionaris inicials a tots els treballadors/es de les respectives unitats		
2. Processament i anàlisi dels qüestionaris i elaboració de l'informe de resultats		
3. Randomització: selecció dels clústers d'intervenció i control		
4. Constitució del Grup Ergo l'encarregat de l'execució del Mètode ERGOPAR, garantint per part de la direcció de l'empresa que cadascun dels seus membres comptarà amb el suport necessaris per al seu avanç		
5. Sessió 1: Formació específica del Grup Ergo		
6. Sessió 2 i 3 reunió identificació problemes i solucions del Grup Ergo		
7. Constitució cercles de prevenció		
8. Presentació dels resultats del Grup Ergo i cercles de prevenció en el Comitè de Seguretat i Salut		
9. Constitució del Grup Operatiu. Planificació e implementació de les mesures proposades		
10. Entrega i recollida de qüestionaris post-intervenció grups d'intervenció i control		
11. Processament i anàlisi dels qüestionaris i elaboració de l'informe sobre l'avaluació de la intervenció		

Signen:

Membres del Comitè de Seguretat i Salut

CURS “ERGONOMIA EN EL ÁMBITO SANITARIO Y MÉTODO ERGOPAR”.

TÍTULO DEL CURSO: “ERGONOMÍA EN EL ÁMBITO SANITARIO Y MÉTODO ERGOPAR”

Servicio de Salud Laboral/Servicio de Prevención, *[nom institució]*

Barcelona, *[data]*

OBJETIVOS

1. Introducir a un grupo de trabajadores del Parc de Salut Mar (PSMAR) expuestos a riesgos ergonómicos en los conceptos básicos de la ergonomía en el ámbito sanitario.
2. Capacitarlos para la identificación de riesgos ergonómicos en sus propios puestos de trabajo y la gestión y propuesta de medidas preventivas, utilizando la metodología ERGOPAR.

METODOLOGÍA DOCENTE

La formación desarrollará los conceptos básicos de ergonomía y la implementación del Método ERGOPAR, adaptado específicamente a las características propias del sector público-sanitario. La formación combinará sesiones teóricas en el aula, con seminarios prácticos.

Los asistentes adquirirán conocimientos sobre Ergonomía Participativa y obtendrán las pautas para la detección de riesgos ergonómicos y la propuesta y aplicación de medidas preventivas, así como los roles y las acciones que deberán llevar a cabo cada uno de los participantes.

Simultáneamente, adquirirán competencias para el desarrollo de dinámicas grupales: coordinación de los participantes, gestión de la información recibida a través de los diversos documentos que se les facilitarán y recopilación de información en cuanto a riesgos ergonómicos existentes dentro de las unidades hospitalarias

Número total de participantes: 16-20 personas. Cada acción formativa contará con el siguiente número de participantes:

- Sesiones teóricas: 10-20 participantes/sesión
- Seminarios prácticos: 5-7 participantes/sesión
- Trabajo de preparación antes y después de cada sesión

DOCENTES

- *[nom ergònom]*, Técnic/a de Prevención de Riesgos Laborales, especialista en Ergonomía. Servicio de Salud Laboral/Servicio de Prevencióbn, *[nom institució]*.

- *[nom champion]*, Centro de Investigación en Salud Laboral (CISAL), Universidad Pompeu Fabra (UPF).

PROGRAMA

Los temas que se tratarán, así como el tiempo de desarrollo, se detallan en la siguiente tabla:

**Sesión**	**Tipo de sesión**	**Contenido**	**Duración**	**Núm. participantes**
1	Sesión teórica (x 2 grupos)	Conceptos básicos en Ergonomía en el trabajo Y Ergonomía participativa.	1h<br>(x2 grupos)	10-20
2	Seminario (x 3 grupos)	Factores de riesgo ergonómicos y efectos sobre la salud en unidades de hospitalización de pacientes con semi y alta dependencia, y área quirúrgica.	1 h.<br>(x3 grupos)	5-7<br>(por grupo)
3	Seminario (x 3 grupos)	Pautas y medias preventivas para reducir los riesgos ergonómicos y efectos sobre la salud en unidades de hospitalización de pacientes con semi y alta dependencia, y área quirúrgica. Elaboración de un plan de trabajo para eliminar o reducir los riesgos ergonómicos.	1 h.<br>(x3 grupos)	5-7<br>(por grupo)
4	Trabajo de preparación antes y después de cada sesión	El trabajo de preparación consiste en la revisión de la documentación, los comentarios a la documentación antes y después de cada sesión, y para los seminarios además el trabajo de discusión con sus equipos antes y después también de cada sesión.	• 2h. sesión teórica<br>• 5h para cada seminario	5-7<br>(por grupo)

Cada alumno participará en un total de 20 h:

- 1h de teoría
- 2h de seminarios prácticos
- 17h de preparación antes y después de cada sesión

CALENDARIO

Se organizarán tres grupos:

- A: equipo de enfermería (enfermeros/as y auxiliares) UH30, H Mar
- B: equipo de enfermería (enfermeros/as y auxiliares) UCI, H Mar

- C: equipo de enfermería (enfermeros/as y auxiliares) LLevants 3 y 4, CAEM

De acuerdo al siguiente calendario:

**Sesión**	**Grupo**	**Fecha**	**Horario**	**Aula**
Teórica	A+B			
Teórica	C			
Seminario 1	A			
Seminario 1	B			
Seminario 1	C			
Seminario 2	A			
Seminario 2	B			
Seminario 2	C			

EVALUACIÓN

Cada participante realizará una prueba escrita tipo test, de 30 preguntas, sobre los conceptos impartidos durante la formación.

Mediante esta prueba se evaluarán los conocimientos y las competencias en Ergonomía y en prevención de riesgos ergonómicos dentro del ámbito sanitario, adquiridas por los participantes.

RECURSOS DIDÁCTICOS

Los materiales didácticos que se utilizarán, así como la documentación necesaria para el desarrollo de las diferentes sesiones formativas serán puestos a disposición de los participantes, para su estudio previo, antes de cada sesión.

PRESENTACIÓN “ERGONOMIA LABORAL”

PROYECTO INTEVAL

Intervención: ERGONOMÍA PARTICIPATIVA

↓

GRUPO ERGO

Sesión 1: Ergonomía Laboral

Índice

1. Ergonomía Laboral
2. Introducción a la Ergonomía Participativa
3. El Método ERGOPAR
4. Material de apoyo: Presentación

1. ERGONOMÍA LABORAL

Objetivos:

- Contar con una base teórica común en ergonomía.
- Entender las situaciones de riesgo ergonómicas en el contexto laboral.
- Capacitar para la identificación de causas de exposición a factores de riesgo ergonómicos.
- Capacitar para la propuesta de medidas preventivas.

---

1. Conceptos y términos sobre ergonomía

1.1. Definiciones de ergonomía

El término ergonomía proviene de las palabras griegas ERGON (Trabajo) y NOMOS (Ley o Norma).

La utilización moderna del término se debe a Murell y fue adoptado oficialmente en julio 1949, a raíz de la creación de la primera Sociedad de ergonomía, la "Ergonomics Research Society" con el fin de "adaptar el trabajo al hombre".

Según la enciclopedia Larousse, se define Ergonomía como el estudio cuantitativo y cualitativo de las condiciones de trabajo en la empresa, que tiene como objeto el establecimiento de técnicas conducentes a una mejora de la productividad y la integración en el trabajo de los productores directos.

Entre las definiciones de los profesionales de la ergonomía encontramos, además de la anteriormente mencionada de Murell, otras como las de Singleton (1969), Faverge (1970), Cazamian (1973), McCormick (1981), Pheasant (1988). De éstas se desprenden tres cuestiones:

- Su principal sujeto de estudio es el hombre en interacción con el medio.
- Su estatus de ciencia normativa.
- Su vertiente de protección de la salud de las personas (física, psíquica y social).

En resumen, podemos decir que la ergonomía trata de alcanzar el mayor equilibrio posible entre las necesidades/posibilidades del usuario y las prestaciones/requerimientos de los productos y servicios.

1.2. Objetivo de la Ergonomía

El objetivo de la ergonomía es mejorar la "calidad de vida" y bienestar de los usuarios, reduciendo los riesgos de error. No se limita a identificar los factores de riesgo y las molestias, sino que propone soluciones positivas, soluciones que se mueven en el ámbito posibilista de las potencialidades efectivas de los usuarios, y de la viabilidad económica de cualquier proyecto.

2. Daños a la salud por trastornos musculoesqueléticos

Los trastornos musculoesqueléticos (TME) son una de las enfermedades de origen laboral más comunes que afectan a millones de trabajadores en toda Europa y cuestan a las empresas miles de millones de euros. Afrontar los TME ayuda a mejorar las vidas de los trabajadores, de la empresa y de la sociedad.

Los trastornos musculoesqueléticos normalmente afectan a la espalda, cuello, hombros y extremidades superiores, aunque también pueden afectar a las extremidades inferiores. Comprenden cualquier daño o trastorno de las articulaciones y otros tejidos. Los problemas de salud abarcan desde pequeñas molestias y dolores, que son los más frecuentes, a cuadros médicos más graves que precisan incapacidad temporal (IT o bajas), e incluso a recibir tratamiento médico. En los casos más crónicos, pueden dar como resultado una discapacidad y la necesidad de dejar de trabajar.

La mayoría de los TME relacionados con el trabajo se desarrollan a lo largo del tiempo. Normalmente no hay una única causa de los TME, sino que son varios los factores que trabajan conjuntamente. Entre las causas físicas y los factores de riesgos organizativos se incluyen:

- Manipulación de cargas, especialmente al agacharse y girarse.
- Movimientos repetitivos o forzados.
- Posturas extrañas o estáticas.
- Vibraciones, iluminación deficiente o entornos de trabajo fríos.
- Trabajo a un ritmo elevado.
- Estar de pie o sentado durante mucho tiempo en la misma posición.

Existen datos crecientes que vinculan los trastornos musculoesqueléticos con factores de riesgo psicosocial (en especial combinados con riesgos físicos), entre los que se incluyen:

- Alto nivel de exigencia de trabajo o una escasa autonomía.
- Escasa satisfacción laboral.

3. El riesgo ergonómico: factores de riesgo y sus causas de exposición

En términos generales, se entiende como "riesgo" la probabilidad de sufrir un determinado evento. Siendo un dato probabilístico, también puede ser entendido como el número de personas de una población que serán afectados por una condición particular.

Serán factores de riesgo ergonómico aquel conjunto de atributos de la tarea o del puesto, más o menos claramente definidos, que inciden en aumentar la probabilidad de que un sujeto, expuesto a ellos, desarrolle una lesión en su trabajo.

Los estudios de campo desarrollados por la OSHA (Occupational Safety and Health Administration), en los Estados Unidos, han permitido establecer la existencia de 5 riesgos que se asocian estrechamente con el desarrollo de lesiones musculoesqueléticas:

1) Desempeñar el mismo movimiento o patrón de movimientos cada varios segundos durante más de dos horas ininterrumpidas.
2) Mantener partes del cuerpo en posturas fijas o posturas peligrosas durante más de dos horas en un turno de trabajo.
3) La utilización de herramientas que producen vibración durante más de dos horas de trabajo.
4) Realizar esfuerzos vigorosos durante más de dos horas de trabajo.
5) Hacer levantamiento manual frecuente o con sobreesfuerzo.

Relación Causa-Efecto: existe mucha evidencia epidemiológica que demuestra una fuerte asociación entre el desarrollo de lesiones músculo tendinosas y determinados factores físicos del trabajo, especialmente cuando existe un alto nivel de exposición y además la exposición es a una combinación de varios factores.

4. Medidas preventivas frente al riesgo ergonómico

Las estrategias de prevención de los trastornos músculoesqueléticos se basan en intervenciones centradas en el lugar de trabajo y en factores individuales. Cada vez se reconoce en mayor medida la necesidad de adoptar un enfoque integrado que incluya ambos tipos de intervención para tratar el problema con eficacia. Las intervenciones ergonómicas se basan en un enfoque «holístico» o sistémico que considera a la vez tanto el efecto del equipo, del entorno laboral y de la organización del trabajo como al trabajador. También es importante el hecho de que los trabajadores participen activamente en su adopción.

Mediante la prevención se pretende:

- Evitar los riesgos.
- Evaluar los riesgos que no se puedan evitar.
- Combatir los riesgos en su origen.
- Adaptar el trabajo a la persona, en particular en lo que respecta a la concepción de los puestos de trabajo, así como a la elección de los equipos de trabajo y los métodos de trabajo y de producción, centrándose especialmente en atenuar el trabajo monótono y el trabajo repetitivo y a reducir los efectos de los mismos en la salud.
- Tener en cuenta la evolución de la técnica.
- Sustituir lo peligroso por lo que entraña poco o ningún peligro.
- Planificar la prevención buscando integrar, de manera coherente, la técnica, la organización del trabajo, las condiciones de trabajo, las relaciones sociales y la influencia de los factores ambientales en el trabajo.
- Adoptar medidas que antepongan la protección colectiva a la individual.
- Dar las debidas instrucciones a los trabajadores.

Las principales estrategias de prevención cubren tanto las estrategias de prevención primaria (eliminación de las causas) como de prevención secundaria (tratamiento y rehabilitación). En este caso también, las opiniones expertas señalan que, si bien debe hacerse especial hincapié en la prevención primaria, es preciso examinar todos estos factores con una visión de conjunto. Por ejemplo, los estudios muestran que es improbable que, por sí sola, la formación sea efectiva si persiste la deficiencia de los factores ergonómicos en el trabajo y que, por ejemplo, es preciso que la formación incluya cómo detectar riesgos potenciales y qué hacer una vez se detectan, así como técnicas seguras de manipulación física.

2. INTRODUCCIÓN A LA ERGONOMIA PARTICIPATIVA

Índice

- ¿Qué es la Ergonomía Participativa?
- ¿Por qué la ergonomía participativa en las empresas?
- Objetivos de la ergonomía Participativa

¿Qué es la Ergonomía Participativa?

No existe una única definición para el término Ergonomía Participativa. Son muchos los autores que han publicado su percepción y punto de vista en revistas científicas y especializadas, al igual que estudios de investigación y revisión que analizan los procedimientos desarrollados y las experiencias implementadas en empresas. Los términos que vienen repitiéndose en las distintas definiciones de ergonomía participativa son: participación, trabajadores, conocimientos, procedimientos, apoyo, mejora, actores, soluciones, implicar e influir. Estas palabras clave permiten unificar el enfoque y la manera de hacer que persigue la ergonomía participativa.

La ergonomía participativa como una estrategia para la mejora de las condiciones de trabajo a nivel ergonómico, estructurada y ordenada por medio de un procedimiento que incorpora la participación activa de los agentes sociales implicados.

¿Por qué aplicar la ergonomía participativa en las empresas?

Las ventajas que ofrecen este tipo de procedimientos, tanto para los trabajadores como para el conjunto de la empresa. Entre otras:

- Promueven la **participación** de los distintos actores en la empresa en la mejora de las condiciones de trabajo.
- Abordan una de las categorías de riesgos laborales que mayor **impacto tiene sobre la salud y el bienestar de los trabajadores**, previniendo su exposición y una de las principales causas de baja laboral.
- Permiten **identificar y tratar muchas situaciones de riesgo** sin necesidad de emplear complicados protocolos técnicos. Potencian la integración de la prevención en la empresa, facilitando la creación de una sólida cultura organizacional preventiva en la empresa.

Organismos como el Health and Safety Executive (HSE, Reino Unido), el Institut de Recherche Robert-Sauvé en Santé et Securité du Travail (IRSST, Québec-Canadá), el Institute for Work and Health (IWH, Ontario-Canadá), la Agencia Europea para la Seguridad y la Salud en el Trabajo (EU-OSHA, estados miembros de la Unión Europea) y el National Institute for Occupational Safety and Health (NIOSH, Estados Unidos), considerados todos ellos organismos e instituciones de referencia internacional en materia de seguridad y salud laboral, han desarrollado procedimientos, revisiones o publicado experiencias en ergonomía participativa de gran calidad, en los que destacan su apuesta por el desarrollo de este tipo de programas. Esta evidencia científica y el apoyo institucional, justifica el interés por desarrollar experiencias de ergonomía participativa en España, donde al igual que en otros países, los trastornos musculoesqueléticos son la patología de origen laboral más prevalente en la población trabajadora (sea considerada o no contingencia profesional), y cuya incidencia persiste año tras año mostrando las carencias del control efectivo del riesgo, en gran parte de las empresas.

Objetivo de la Ergonomía Participativa

El objetivo de la ergonomía participativa es la mejora de las condiciones de trabajo a nivel ergonómico y la prevención de los trastornos musculoesqueléticos.

La literatura nos muestra cual debe ser el objetivo de la ergonomía participativa. Aquí tenemos algunos ejemplos:

"Incrementar los conocimientos y la conciencia de los trabajadores respecto a la ergonomía en su puesto de trabajo, motivándolos a participar activamente en el programa de intervención, y en la aplicación de medidas ergonómicas en el centro de trabajo"

"Proporcionar a empresarios y trabajadores con información y asesoramiento un marco genérico (directrices) que les permita prevenir los trastornos musculoesqueléticos (TME) en su lugar de trabajo, y aplicar un programa y materiales efectivos e integrar la prevención de los TME en la organización de la empresa"

El objetivo goza de un mayor consenso, ya que la meta común coincide en que debe ser **la mejora de las condiciones de trabajo a nivel ergonómico y la prevención de los trastornos musculoesqueléticos.**

3. METODOLOGÍA ERGOPAR

Índice

- El Método ErgoPar, ¿en qué consiste?
- Fases del método ErgoPar
- Fase Intervenció
    - Identificación y análisis
    - Propuesta y planificación
    - Seguimiento

El Método ErgoPar, ¿en qué consiste?

El Método ERGOPAR es un procedimiento de ergonomía participativa para la prevención del riesgo ergonómico de origen laboral. Se ha concebido para identificar la exposición a factores de riesgo ergonómicos a consecuencia del trabajo y sus causas de exposición, consensuar las mejores medidas preventivas para la eliminación o al menos, reducción de las situaciones de riesgo, implementarlas y realizar su seguimiento y mejora continua. **(http://ergopar.istas.net/)**

El Método ErgoPar tiene un doble objetivo:

- La mejora continua de las condiciones de trabajo a nivel ergonómico mediante la implementación de medidas preventivas que eliminen o al menos, reduzcan la exposición a factores de riesgo, y
- facilitar la participación de los trabajadores, sus representantes legales y demás actores implicados en la prevención de riesgos laborales en la empresa.

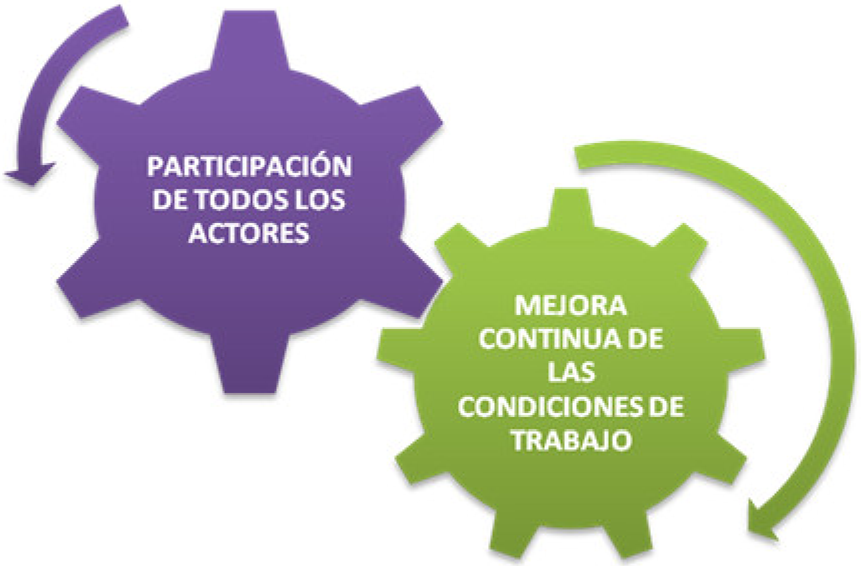

Las ventajas de llevar a cabo un procedimiento de ergonomía participativa siguiendo el Método ERGOPAR son muchas, entre ellas destacamos las siguientes:

- Incorpora el conocimiento y experiencia de todas las personas implicadas en la experiencia, destacando el proporcionado por los trabajadores que pasan a integrarse en las actuaciones preventivas en la empresa.
- Genera dinámicas y habilidades participativas y grupales que permanecen en la empresa. La principal ventaja de la ergonomía participativa es que se crea en la empresa “un saber hacer en prevención”. Mediante la formación y la experiencia, por ejemplo, los miembros del Grupo Ergo adquieren destrezas que les permiten analizar y resolver situaciones de riesgo ergonómico. Este conocimiento y las habilidades participativas en las que se ven inmersos son fundamentales para promover la continuidad del programa y la integración de la prevención de riesgos laborales en la empresa.
- Facilita la propuesta de medidas preventivas adaptadas a las necesidades y circunstancias reales de la empresa y los trabajadores. Las medidas preventivas a aplicar son propuestas, planificadas e implementadas por el personal de la empresa, lo que permite su mejor integración en las prácticas internas, respetando las peculiaridades culturales, económicas y técnicas del centro de trabajo.
- Permite flexibilidad y adaptación a los distintos contextos empresariales.
- Favorece la aceptación de las medidas preventivas. La resistencia al cambio se atenúa e incluso, desaparece en la medida en que los propios afectados participan en la

identificación de factores de riesgo ergonómico y daños de origen laboral, en la propuesta de medidas preventivas y en su seguimiento.

- Acorta los tiempos de implementación de las medidas preventivas. En base a los recursos disponibles en la empresa (económicos, tiempo, disponibilidad…) es posible acortar los plazos hasta la ejecución de las medidas preventivas.
- Permite visualizar la implicación de la dirección de la empresa en la prevención de los riesgos ergonómicos. Esta implicación, voluntariedad y compromiso firme de la dirección se visualiza desde el primer momento en el que se acuerda la aplicación del método.
- Promueve la mejora de las relaciones laborales en el centro de trabajo. El proceso participativo mejora la comunicación en la empresa y permite argumentar y aproximar las distintas perspectivas sobre las situaciones de riesgo ergonómico y el consenso de las medidas preventivas a implementar, facilitando el cumplimiento de derechos básicos de los trabajadores y sus representantes legales.

Fases del método ErgoPar

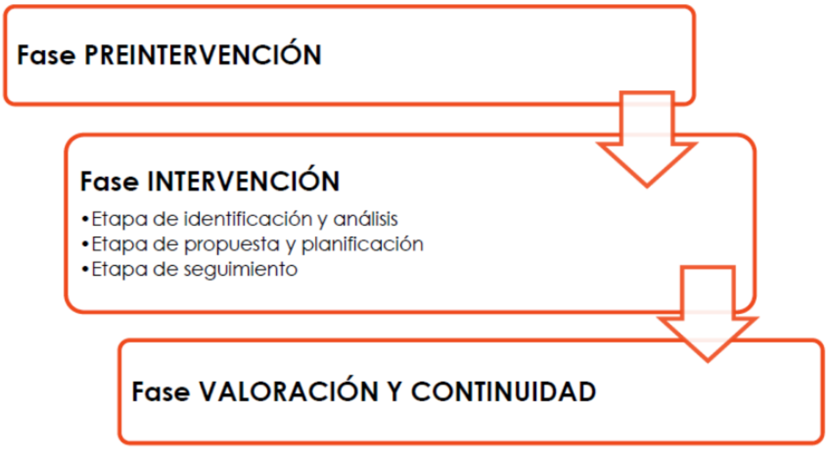

Fase de intervención

- **Identificación y análisis**

El objetivo de esta etapa es identificar exposiciones a factores de riesgo ergonómicos y daños prioritarios en los puestos de trabajo por colectivo homogéneo, para después identificar las causas de exposición a dichos factores de riesgo.

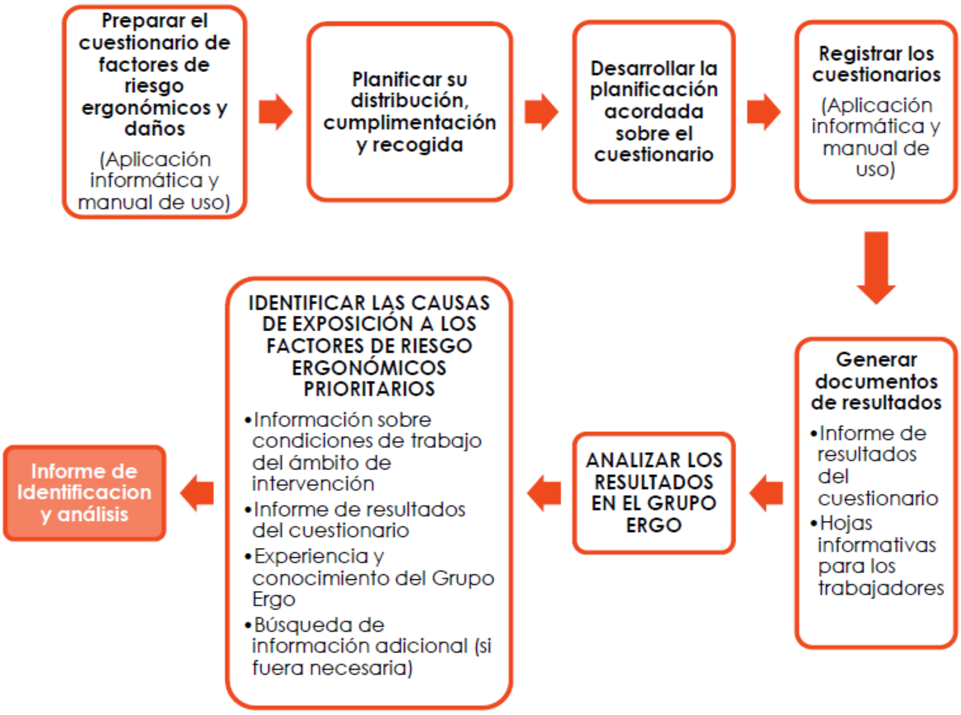

(relacionado cuestionario ErgoPar + Evaluación de la UH)

La etapa de identificación y análisis supone una parte importantísima en el conjunto del procedimiento, dado que de la información que aquí se genere, dependerá la propuesta de medidas preventivas. Una inadecuada campaña informativa (sensibilización), una deficiente distribución o cumplimentación del cuestionario, o una falta de dedicación o conocimientos por parte del Grupo Ergo en la identificación de causas, puede poner en peligro el éxito de la experiencia participativa, generando una información de escasa representatividad y poco realista.

- **Propuesta y Planificación**

El objetivo de la etapa de propuesta y planificación es obtener una propuesta de medidas preventivas consensuadas y priorizadas, que elimine o al menos reduzca la exposición a las situaciones de riesgo ergonómico identificadas (factores de riesgo y sus causas de exposición), y acordar su planificación e implementación en el ámbito de intervención.

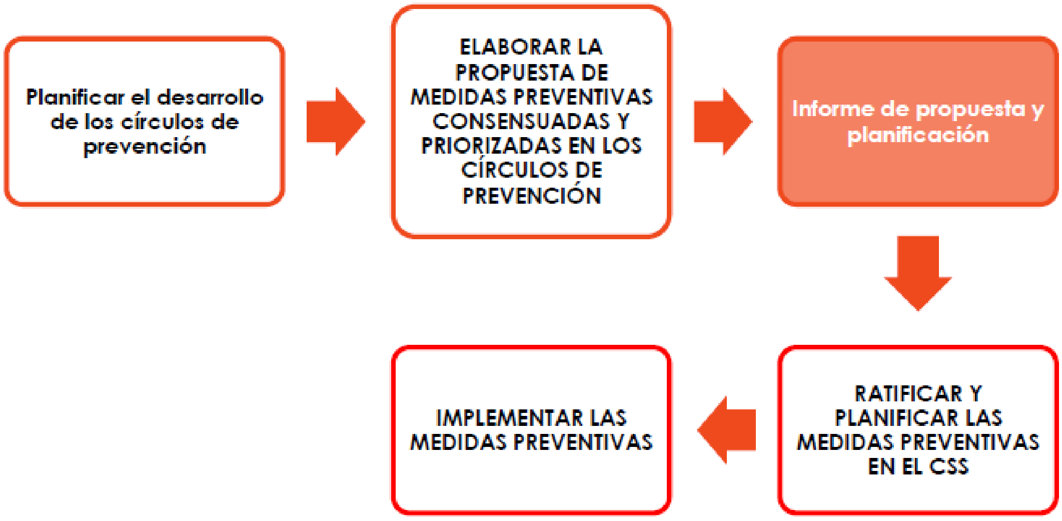

Para el logro de este objetivo, el Método ERGOPAR proporciona herramientas que favorecen la consecución de las tareas. Son las siguientes: En la búsqueda de medidas preventivas se empleará un guión para el desarrollo de círculos de prevención. En la elaboración de la propuesta de medidas preventivas consensuada y priorizada se utilizará, una ficha de trabajo. Para la planificación de las medidas preventivas a ejecutar se facilita una ficha de planificación a integrar en el documento de planificación de acciones preventivas de la empresa.

- **Seguimiento**

La etapa de seguimiento tiene un doble objetivo: controlar que se cumple en tiempo y forma la ejecución de la planificación de medidas preventivas acordadas y evaluar la eficacia de las medidas preventivas implementadas y en su caso, corregir su ineficacia para la mejora continua.

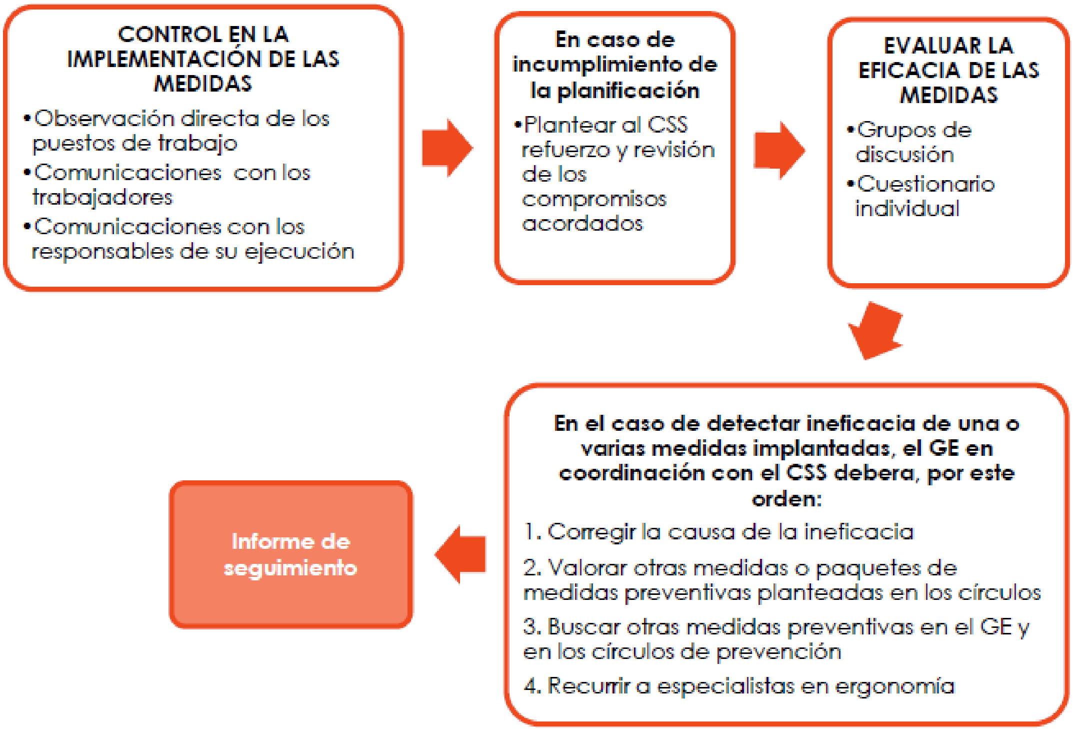
CONTROL EN LA IMPLEMENTACIÓN DE LAS MEDIDAS
•Observación directa de los puestos de trabajo
•Comunicaciones con los trabajadores
•Comunicaciones con los responsables de su ejecución
En caso de incumplimiento de la planificación
•Plantear al CSS refuerzo y revisión de los compromisos acordados
EVALUAR LA EFICACIA DE LAS MEDIDAS
•Grupos de discusión
•Cuestionario individual
En el caso de detectar ineficacia de una o varias medidas implantadas, el GE en coordinación con el CSS debera, por este orden:
1. Corregir la causa de la ineficacia
2. Valorar otras medidas o paquetes de medidas preventivas planteadas en los círculos
3. Buscar otras medidas preventivas en el GE y en los círculos de prevención
4. Recurrir a especialistas en ergonomía
Informe de seguimiento

LOGO HOSPITAL

HOJA DE REGISTRO DE PROBLEMAS

UNIDAD:

		**FACTORES DE RIESGO ERGONÓMICO**							
		**Manipulación de cargas/pacientes**					**Postura Forzada**		
Tarea/Actividad	Descripción de la actividad/tarea	Características de la carga	Esfuerzo físico necesario	Características del medio de trabajo	Exigencias de la actividad	Factores individuales de riesgo	Frecuencia de movimientos	Duración de la postura	Postura

ROL Y PARTICIPACIÓN DE LOS/LAS REFERENTES DE LAS UNIDADES/ CLÚSTERS

PROYECTO INTEVAL_Spain: ROL Y PARTICIPACIÓN DE LOS/LAS REFERENTES DE LAS UNIDADES/*CLÚSTERS*

La intervención del proyecto INTEVAL_Spain consta de tres componentes: ergonomía participativa (EP), gestión de casos y Promoción de la saud. La EP precisa del grupo ERGO, que se compone del/la coordinadora, expertos/as en ergonomía, jefes/as y supervisores/as de las unidades participantes y un grupo de 4-6 profesionales de cada unidad que representen los diferentes turnos. Estos/as profesionales son los/as REFERENTES.

Las funciones de los/as referentes van más allá del grupo ERGO. Tienen, además, un rol dinamizador dentro de su grupo (turno) en la unidad a lo largo del proyecto y de puente de comunicación entre el resto de trabajadores/as de las unidades/*clústers* participantes y la coordinación del proyecto.

Estas funciones son:

1. Entender las situaciones de riesgo ergonómico en el contexto laboral y sus causas, y proponer mejoras de las condiciones de trabajo.

2. Promover la intervención de EP involucrando al resto de profesionales de su unidad/*clúster* y turno en la detección de situaciones de riesgo ergonómico y propuesta de medidas de mejora.

3. Mantener la comunicación con la coordinación del proyecto INTEVAL_Spain, a través del Whatsapp y/o el correo electrónico, y con el equipo de profesionales de su unut¡dad/turno.

4. Consultar y comentar los materiales formativos que serán puestos a su disposición por el Equipo Técnico INTEVAL_Spain durante las sesiones del grupo ERGO.

5. Acudir a las reuniones previstas y participar de manera activa, transmitiendo la información recopilada en la unidad/*clúster* y aportando las observaciones que crean oportunas.

6. Recopilar información detallada sobre las condiciones de trabajo de la unidad/*clúster*.

7. Promover el intercambio de ideas y la innovación entre los/las profesionales de la unidad/*clúster* para la obtención de propuestas de medidas preventivas.

8. Promover y facilitar la comunicación y el consenso entre los profesionales de la unidad/*clúster* durante toda la intervención.

9. Mantener un circuito de comunicación activa y fluida con los/las jefes/as y supervisores/as de la unidad*/clúster*, transmitiéndoles a la mayor brevedad posible cualquier sugerencia, duda o problema que pueda surgir durante el transcurso de la intervención.

10. Colaborar con la coordinación del proyecto INTEVAL_Spain en la organización de actividades que tengan relación con la unidad/*clúster* que representan a lo largo del proyecto.

HORAS DE FORMACIÓN PROFESIONAL POR EL CURSO: "ERGONOMÍA EN EL ÁMBITO SANITARIO Y MÉTODO ERGOPAR"

Servicio de Salud Laboral, Parc de Salut Mar

Barcelona, abril de 2019

OBJETIVOS

3. Introducir a un grupo de trabajadores del Parc de Salut Mar (PSMAR) expuestos a riesgos ergonómicos en los conceptos básicos de la ergonomía en el ámbito sanitario.
4. Capacitarlos para la identificación de riesgos ergonómicos en sus propios puestos de trabajo y la gestión y propuesta de medidas preventivas, utilizando la metodología ERGOPAR.

METODOLOGÍA DOCENTE

La formación desarrollará los conceptos básicos de ergonomía y la implementación del Método ERGOPAR, adaptado específicamente a las características propias del sector público-sanitario. La formación combinará sesiones teóricas en el aula, con seminarios prácticos.

Los asistentes adquirirán conocimientos sobre Ergonomía Participativa y obtendrán las pautas para la detección de riesgos ergonómicos y la propuesta y aplicación de medidas preventivas, así como los roles y las acciones que deberán llevar a cabo cada uno de los participantes.

Simultáneamente, adquirirán competencias para el desarrollo de dinámicas grupales: coordinación de los participantes, gestión de la información recibida a través de los diversos documentos que se les facilitarán y recopilación de información en cuanto a riesgos ergonómicos existentes dentro de las unidades hospitalarias

Número total de participantes: 16-20 personas. Cada acción formativa contará con el siguiente número de participantes:

- Sesiones teóricas: 10-20 participantes/sesión
- Seminarios prácticos: 5-7 participantes/sesión
- Trabajo de preparación antes y después de cada sesión

DOCENTES

- Chelo Sancho, Técnic/a de Prevención de Riesgos Laborales, especialista en Ergonomía. Servicio de Salud Laboral, Parc de Salut Mar
- Olga Martínez, Champion proyecto; Centro de Investigación en Salud Laboral (CISAL), IMIM-UPF.

PROGRAMA

Los temas que se tratará cada grupo, así como el tiempo de desarrollo, se detallan en la siguiente tabla:

Sesión	Tipo de sesión	Contenido	Duración	Núm. participantes
1	Sesión teórica	Conceptos básicos en Ergonomía en el trabajo Y Ergonomía participativa.	1h	10-20
2	Seminario	Factores de riesgo ergonómicos y efectos sobre la salud en unidades de hospitalización de pacientes con semi y alta dependencia, y área quirúrgica.	1 h	5-7
3	Seminario	Pautas y medias preventivas para reducir los riesgos ergonómicos y efectos sobre la salud en unidades de hospitalización de pacientes con semi y alta dependencia, y área quirúrgica. Elaboración de un plan de trabajo para eliminar o reducir los riesgos ergonómicos.	1 h	5-7
4	Trabajo de preparación antes y después de cada sesión	El trabajo de preparación consiste en la revisión de la documentación, los comentarios a la documentación antes y después de cada sesión, y para los seminarios además el trabajo de discusión con sus equipos antes y después también de cada sesión.	• 2h. sesión teórica<br>• 5h para cada seminario	5-7

Cada alumno participará en un total de 20 h:

- 1h de teoría
- 2h de seminarios prácticos
- 17h de preparación antes y después de cada sesión

CALENDARIO

Se organizarán tres grupos:

- A: equipo de enfermería (enfermeros/as y auxiliares): **UH30, Hospital de l'Esperança.**
- B: equipo de enfermería (enfermeros/as y auxiliares): **UH07, Hospital del Mar.**

El calendario y horarios de cada grupo se muestra en la siguiente tabla:

Sesión	Grupo	Fecha	Horario	Aula
Teórica	A	08/05/2019	15:30-17:00	Sala docent 7ªpl. H. de la Esperança
Teórica	B	08/05/2019	14:00-15:30	Sala2, 10ªPl. H.del Mar
Seminario 1	A	22/05/2019	15:30-17:00	Sala polivalente 4ªpl. H. de la Esperança
Seminario 1	B	22/05/2019	14:00-15:30	Sala 2, 10ªPl. H.del Mar
Seminario 2	A	29/05/2019	15:30-17:00	Sala polivalente 4ªpl. H. de la Esperança
Seminario 2	B	29/05/2019	14:00-15:30	Sala 2, 10ªPl. H.del Mar

EVALUACIÓN

Cada participante realizará una prueba escrita tipo test sobre los conceptos impartidos durante la formación.

Mediante esta prueba se evaluarán los conocimientos y las competencias en Ergonomía y en prevención de riesgos ergonómicos dentro del ámbito sanitario, adquiridas por los participantes.

RECURSOS DIDÁCTICOS

Los materiales didácticos que se utilizarán, así como la documentación necesaria para el desarrollo de las diferentes sesiones formativas serán puestos a disposición de los participantes, para su estudio previo, antes de cada sesión.

CONSENTIMIENTO INFORMADO

DECLARACIÓN DE PARTICIPACIÓN VOLUNTARIA COMO PROFESIONAL REFERENTE DEL PROYECTO INTEVAL_Spain:

Declaro que he sido informado/a de la naturaleza y el propósito de la fase de EP, de los datos que se me pide que proporcione y de las acciones que llevaré a cabo mediante mi participación como referente de la unidad/*clúster* a la que represento. He recibido una explicación satisfactoria sobre los procedimientos de la fase de EP y su finalidad.

Comprendo que mi decisión de participar es voluntaria y doy mi consentimiento para la participación en las acciones que se realizarán durante el desarrollo de la fase de EP. Conozco mi derecho a retirar este consentimiento cuando lo desee, con la única obligación de informar mi decisión, a la mayor brevedad posible, al Equipo Investigador INTEVAL_ plus.

Por favor, si ACEPTA PARTICIPAR voluntariamente en la fase de EP del Proyecto INTEVAL_Spain, firme a continuación e indíquenos su nombre y apellidos, así como la fecha actual.

Firma trabajador/a:	Firma de la persona que obtiene el consentimiento:
Nombre trabajador/a:	(Nombre)
Fecha:	Fecha:

LOGO HOSPITAL

FULL DE REGISTRE POSSIBLES SOLUCIONS

UNIDAD:

FICHA DE PROPUESTA DE MEDIDAS PREVENTIVAS PARTICIPANTES DEL "CERCLES DE PREVENCIÓ"			
**PUESTO DE TRABAJO:**			
**TAREA**	**CAUSAS**	**ESCALA**	**MEDIDAS PREVENTIVAS**

LOGO HOSPITAL

INFORME PROPUESTAS MEDIDAS DE MEJORA

INFORMACIÓN DIRIGIDA A LOS "CERCLES DE PREVENCIÓ"

**FICHA INFORMACIÓN DE IDENTIFICACIÓN DE CAUSAS FACTORES RIESGOS ERGONOMICOS**	
Unidad:	Puesto:
Tareas/situaciones seleccionadas (orden de prioridad de 1 a 5)<br><br>1-<br>2-<br>3-<br>4-<br>5-	
**CLASIFICACIÓN**	**CAUSAS DE EXPOSICIÓN A LOS FACTORES DE RIESGOS**
Organización de trabajo	
Equipos y mobiliario	
Materiales y productos	
Entorno de trabajo y condiciones ambientales	
Tipología paciente	
Ropa de trabajo	

ERGOPAR

Un procedimiento de ergonomía participativa para la prevención del riesgo ergonómico de origen laboral

LOGO HOSPITAL

TAULA DE PLANIFICACIÓ (FORMATO EXCEL)

ERGONOMIA PARTICIPATIVA, GRUP HOSPITAL . TAULA PLANIFICACIÓ - GRUP OPERATIU

Data reunió XXX

Participen: XXXX

ID	**Mesura general**	**Mesura general, descripció**	**Tipus proposta**	**Proposta**	**Proposta descripció**	**Factibilitat**	**Responsables**	**Previsió**	**Cost afegit**	**Estat actual**	**Seguiment**

ANEXO 4: CUESTIONARIO GESTIÓN DE CASOS

Buenos días / Buenas tardes:

A continuación procedo a realizarle unas preguntas, con la finalidad de obtener información útil para poder realizar su seguimiento y orientar los servicios de salud que pueda necesitar.

Todo el tratamiento de los datos obtenidos lo realizará, de manera confidencial, el equipo investigador.

Es muy importante que responda de manera concreta y con total sinceridad. Si no entiende alguna de las preguntas o de las respuestas, por favor, no dude en preguntar.

START BACK SCREENING TOOL: DOLOR DE ESPALDA (9 preguntas)

Piense en las últimas **2** semanas y marque su respuesta a las siguientes preguntas:

		Desacuerdo	De acuerdo
		0	1
**1**	Mi dolor de espalda **se ha extendido a lo largo de mi pierna (s)** en alguna ocasión en las últimas dos semanas	☐	☐
**2**	Me ha dolido el **hombro** o **cuello** en alguna ocasión en las últimas dos semanas	☐	☐
**3**	En las últimas dos semanas, sólo he **caminado distancias cortas** por mi dolor de espalda	☐	☐
**4**	En las últimas dos semanas, me he **vestido más lentamente** de lo normal por mi dolor de espalda	☐	☐
**5**	No es seguro ser **físicamente activo** con mi dolor de espalda	☐	☐
**6**	Me he **preocupado** mucho por mi dolor de espalda en las últimas dos semanas	☐	☐
**7**	Noto que **mi dolor de espalda es terrible** y que **nunca irá a mejor**	☐	☐
**8**	En general en las últimas dos semanas, no he **disfrutado** de las cosas lo que habitualmente disfruto	☐	☐

9 En general, cómo le ha **molestado su espalda** en las últimas dos semanas:

Nada	Un poco	Moderadamente	Mucho	Extremadamente
☐	☐	☐	☐	☐
0	0	0	1	1

SOMATIZACIÓN – CUPID (5 preguntas)

A continuación, le recito una lista de cinco problemas que las personas normalmente tienen. Por favor, atienda cuidadosamente a cada uno y dígame **CUÁNTO LE HAN MOLESTADO O PREOCUPADO ESOS PROBLEMAS** durante los **ÚLTIMOS 7 DÍAS, INCLUYENDO EL DÍA DE HOY.**

		Nada	Muy poco	Algo	Bastante	Mucho
**10**	Desmayos o mareos	0	1	2	3	4
**11**	Dolores en el corazón o tórax	0	1	2	3	4
**12**	Náusea o molestia en el estómago	0	1	2	3	4
**13**	Problemas para respirar	0	1	2	3	4
**14**	Periodos de frío o calor	0	1	2	3	4

GHQ-12 (12 preguntas)

Ahora nos gustaría saber cómo se ha sentido, en general, **durante las últimas semanas.** Por favor, conteste a todas las preguntas indicando la respuesta que, a su juicio, mejor puede aplicarse a usted. Recuerde que sólo debe responder sobre los problemas recientes y los que tiene ahora, no sobre los que tuvo en el pasado.

**15**	**¿Ha podido concentrarse bien en lo que hacía?**				
Mejor que lo habitual	Igual que lo habitual	Menos que lo habitual	Mucho menos que lo habitual	No sabe	No contesta
0	1	2	3	8	9

**16**	**¿Sus preocupaciones le han hecho perder mucho sueño?**				
No, en absoluto	No más que lo habitual	Algo más que lo habitual	Mucho más que lo habitual	No sabe	No contesta
0	1	2	3	8	9

**17**	**¿Ha sentido que está desempeñando un papel útil en la vida?**				
Más útil que lo habitual	Igual que lo habitual	Menos útil que lo habitual	Mucho menos útil que lo habitual	No sabe	No contesta
0	1	2	3	8	9

**18**	**¿Se ha sentido capaz de tomar decisiones?**				
Más que lo habitual	Igual que lo habitual	Menos que lo habitual	Mucho menos que lo habitual	No sabe	No contesta
0	1	2	3	8	9

**19**	**¿Se ha notado constantemente agobiado y en tensión?**				
No, en absoluto	No más que lo habitual	Algo más que lo habitual	Mucho más que lo habitual	No sabe	No contesta
0	1	2	3	8	9

**20**	**¿Ha tenido la sensación de que no puede superar sus dificultades?**				
No, en absoluto	No más que lo habitual	Algo más que lo habitual	Mucho más que lo habitual	No sabe	No contesta
0	1	2	3	8	9

**21**	**¿Ha sido capaz de disfrutar de sus actividades normales de cada día?**				
Más que lo habitual	Igual que lo habitual	Menos que lo habitual	Mucho menos que lo habitual	No sabe	No contesta
0	1	2	3	8	9

**22**	**¿Ha sido capaz de hacer frente adecuadamente a sus problemas?**				
Más capaz que lo habitual	Igual que lo habitual	Menos capaz que lo habitual	Mucho menos capaz que lo habitual	No sabe	No contesta
0	1	2	3	8	9

**23**	**¿Se ha sentido poco feliz o deprimido?**

No, en absoluto	No más que lo habitual	Algo más que lo habitual	Mucho más que lo habitual	No sabe	No contesta
0	1	2	3	8	9

**24**	**¿Ha perdido confianza en sí mismo?**				
No, en absoluto	No más que lo habitual	Algo más que lo habitual	Mucho más que lo habitual	No sabe	No contesta
0	1	2	3	8	9

**25**	**¿Ha pensado que usted es una persona que no vale para nada?**				
No, en absoluto	No más que lo habitual	Algo más que lo habitual	Mucho más que lo habitual	No sabe	No contesta
0	1	2	3	8	9

**26**	**¿Se siente razonablemente feliz considerando todas las circunstancias?**				
Más que lo habitual	Igual que lo habitual	Menos que lo habitual	Mucho menos que lo habitual	No sabe	No contesta
0	1	2	3	8	9

EQ - 5D (6 preguntas)

Seleccione la afirmación en cada sección que describa mejor su estado de salud en el día de hoy:

**27**	**Movilidad**				
No tengo problemas para caminar	☐	Tengo algunos problemas para caminar	☐	Tengo que estar en la cama	☐

**28**	**Cuidado personal**				
No tengo problemas con el cuidado personal	☐	Tengo algunos problemas para lavarme o vestirme solo	☐	Soy incapaz de lavarme o vestirme solo	☐

**29**	**Actividades de <u>Todos los Días</u>** (ej, trabajar, estudiar, hacer tareas domésticas, actividades familiares o realizadas durante el tiempo libre)				
No tengo problemas para realizar mis actividades de todos los días	☐	Tengo algunos problemas para realizar mis actividades de todos los días	☐	Son incapaz de realizar mis actividades de todos los días	☐

**30**	**Dolor/Malestar**				
No tengo dolor ni malestar	☐	Tengo moderado dolor o malestar	☐	Tengo mucho dolor o malestar	☐

**31**	**Ansiedad/Depresión**				
No estoy ansioso/a ni deprimido/a	☐	Estoy moderadamente ansioso/a o deprimido/a	☐	Estoy muy ansioso/a o deprimido/a	☐

Para ayudar a la gente a describir lo bueno o lo malo que es su estado de salud, hemos dispuesto una escala parecida a un termómetro en el cual se marca con un 100 el mejor estado de salud que pueda imaginarse, y con un 0 el peor estado de salud que pueda imaginarse.

32 Por favor, de 0 a 100, ¿cómo puntuaría **su estado de salud hoy?**

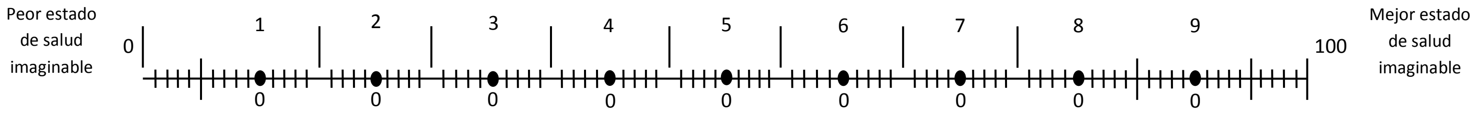

AUTOEFICACIA (8 preguntas)

33	Puedo encontrar la forma de obtener lo que quiero aunque alguien se me oponga				
Nunca o casi nunca	Solo una vez	Algunas veces	Muchas veces	Siempre	NS/NC
☐	☐	☐	☐	☐	☐

34	Puedo resolver problemas difíciles si me esfuerzo lo suficiente				
Nunca o casi nunca	Solo una vez	Algunas veces	Muchas veces	Siempre	NS/NC
☐	☐	☐	☐	☐	☐

35	Me es fácil persistir en lo que me he propuesto hasta llegar a alcanzar mis metas				
Nunca o casi nunca	Solo una vez	Algunas veces	Muchas veces	Siempre	NS/NC
☐	☐	☐	☐	☐	☐

36	Tengo confianza en que podría manejar eficazmente acontecimientos inesperados				
Nunca o casi nunca	Solo una vez	Algunas veces	Muchas veces	Siempre	NS/NC
☐	☐	☐	☐	☐	☐

37	Gracias a mis cualidades y recursos puedo superar situaciones imprevistas				
Nunca o casi nunca	Solo una vez	Algunas veces	Muchas veces	Siempre	NS/NC
☐	☐	☐	☐	☐	☐

38	Puedo resolver la mayoría de los problemas si me esfuerzo lo necesario				
Nunca o casi nunca	Solo una vez	Algunas veces	Muchas veces	Siempre	NS/NC
☐	☐	☐	☐	☐	☐

39	Si me encuentro en una situación difícil, generalmente se me ocurre qué debo hacer				
Nunca o casi nunca	Solo una vez	Algunas veces	Muchas veces	Siempre	NS/NC

☐	☐	☐	☐	☐	☐

**40**	**Al tener que hacer frente a un problema, generalmente se me ocurren varias alternativas de cómo resolverlo**				
Nunca o casi nunca	Solo una vez	Algunas veces	Muchas veces	Siempre	NS/NC
☐	☐	☐	☐	☐	☐

HÁBITOS SALUDABLES (9 preguntas)

**41**	**¿Cuántas horas a la semana realiza ejercicio físico (hacer gimnasia, natación, dar paseos…) en su tiempo libre?**			
No realiza ejercicio físico (Salto a la pregunta 43)	Menos de dos horas a la semana	Entre 2 y 4 horas a la semana	Más de 4 horas a la semana	NS/NC
☐	☐	☐	☐	☐

**42**	**¿Qué tipo de ejercicio físico realiza en su tiempo libre?**		
De intensidad leve (ej. Andar)	De intensidad moderada (ej. natación, tenis)	De intensidad alta (ej. gimnasia de competición)	NS/NC
☐	☐	☐	☐

**43**	**¿Ha habido alguna vez un periodo de su vida, en que usted haya fumado tabaco todos los días?**		
Sí, alguna vez he fumado todos los días	No he fumado en ninguna ocasión todos los días	Nunca he fumado un cigarrillo u otro tipo de tabaco (Salto a la pregunta 46)	NS/NC
☐	☐	☐	☐

**44**	**¿Cuánto tiempo ha pasado desde la última vez que usted fumó un cigarrillo u otro tipo de tabaco?**		
Menos de 30 días	Más de 1 mes pero menos de un año	Más de 12 meses (Salto a la pregunta 46)	NS/NC
☐	☐	☐	☐

**45**	**Durante los últimos 30 días, por término medio, ¿qué número de cigarrillos, puros o pipas solía fumar cada día que fumaba? Dígame el número medio o seleccione la opción correspondiente.**	
No he fumado un cigarrillo u otro tipo de tabaco en los últimos 30 días		NS/NC
☐		☐
Nº cigarrillos:	Nº puros:	Nº pipas:

**46**	**¿Cuántas horas calcula que habrá dormido verdaderamente cada noche durante el último mes?**
Horas: …………………	NS/NC ☐

**47**	**Durante el último mes, cuántas veces ha tenido usted problemas para dormir a causa de: No poder conciliar el sueño en la primera media hora**			
Ninguna en el último mes	Menos de una vez a la semana	Una o dos veces a la semana	Tres o más veces a la semana	NS/NC

☐	☐	☐	☐	☐

48	Durante el último mes, cuántas veces ha tenido usted problemas para dormir a causa de: Despertarse durante la noche o madrugada			
Ninguna en el último mes	Menos de una vez a la semana	Una o dos veces a la semana	Tres o más veces a la semana	NS/NC
☐	☐	☐	☐	☐

49	Durante el último mes, ¿cómo valoraría, en conjunto, la calidad de su sueño?			
Muy buena	Bastante buena	Bastante mala	Muy mala	NS/NC
☐	☐	☐	☐	☐

COMORBILIDAD (1 pregunta)

50	¿Padece o ha padecido alguna enfermedad o problema de salud de larga duración, es decir, que haya durado o que se espere que dure 6 meses o más? En caso afirmativo, ¿cuánto le interfiere en el desarrollo de sus actividades diarias? en una escala de 1 (no me limita) a 5 (me limita mucho)					
		1	2	3	4	5
☐	Alergia	☐	☐	☐	☐	☐
☐	Angina de pecho	☐	☐	☐	☐	☐
☐	Ansiedad relacionada con el estrés	☐	☐	☐	☐	☐
☐	Artritis/osteoartritis	☐	☐	☐	☐	☐
☐	Asma	☐	☐	☐	☐	☐
☐	Cáncer	☐	☐	☐	☐	☐
☐	Patología cardíaca isquémica	☐	☐	☐	☐	☐
☐	Patología hepática	☐	☐	☐	☐	☐
☐	Patología renal	☐	☐	☐	☐	☐
☐	EPOC (enfermedad pulmonar obstructiva crónica)	☐	☐	☐	☐	☐
☐	Depresión	☐	☐	☐	☐	☐
☐	Ansiedad	☐	☐	☐	☐	☐
☐	Diabetes	☐	☐	☐	☐	☐
☐	Epilepsia	☐	☐	☐	☐	☐
☐	Arritmia	☐	☐	☐	☐	☐
☐	Insuficiencia cardíaca	☐	☐	☐	☐	☐
☐	Colesterol alto	☐	☐	☐	☐	☐
☐	Hipertensión	☐	☐	☐	☐	☐
☐	Dolor lumbar (lumbago/ciática)	☐	☐	☐	☐	☐

☐	Dolor cervical	☐	☐	☐	☐	☐
☐	Infarto de miocardio	☐	☐	☐	☐	☐
☐	Osteoporosis	☐	☐	☐	☐	☐
☐	Íctus (accidente cerebrovascular)	☐	☐	☐	☐	☐
☐	Trastorno tiroideo	☐	☐	☐	☐	☐
☐	NS/NC	☐	☐	☐	☐	☐

ANEXO 5: PAUTA PARA EL CIERRE DE CASOS Y PERIODICIDAD DEL SEGUIMIENTO MOTIVACIONAL

Pauta cierre de casos y periodicidad del seguimiento motivacional

1. Cierre de casos

Trabajador de bajo riesgo sin IT se estima que el cierre se realizará aproximadamente un mes después del primer contacto, y en el caso de trabajador de bajo riesgo con IT, si éste recibe el alta antes de un mes se actuará como el caso anterior, y en el caso de que un trabajador de bajo riesgo siga en IT al cabo de un mes, se valorará el caso en la sesión de casos.

Trabajador de riesgo medio o alto, se cerrará el caso cuando se cumpla al menos uno de los siguientes supositos: los profesionales de los servicios consideren que el trabajador ya no necesita más sesiones; en la sesión de casos semanal se valore que su limitación funcional ha desaparecido/disminuido y que no necesita más sesiones; o bien, cuando él mismo decida dejar de participar (independientemente de si es por mejora o no).

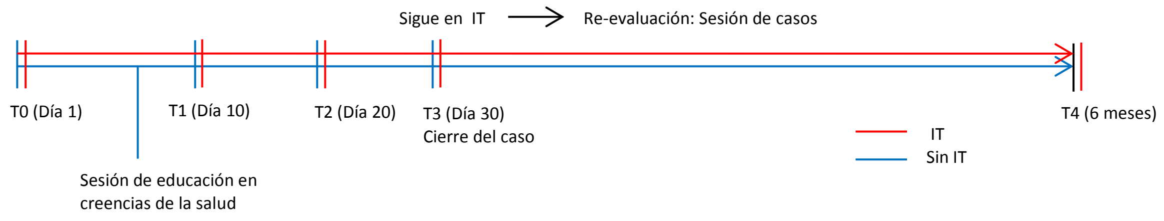

2. Seguimiento motivacional.

En trabajadores de bajo riesgo se estiman unos 5 contactos telefónicos: T0 (día 1), 1r contacto (entrevista y cuestionario); T1 (día 10), 2ndo contacto (posterior a la sesión de educación en creencias de la salud); T2 (día 20), 3r contacto (seguimiento motivacional, recordatorio creencias); T3 (día 30), 4to contacto (cierre del caso), aproximadamente 1 mes después del primer contacto); T4, 5to contacto (cuestionario 6 meses posterior al cierre del caso).

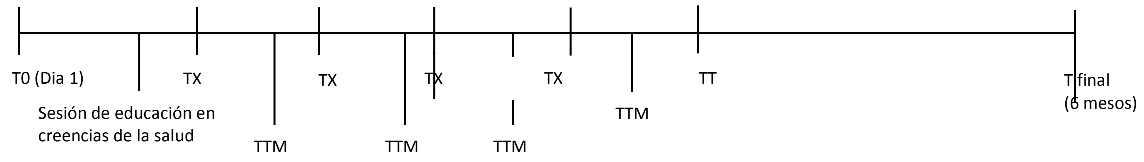

En trabajadores de riesgo medio o alto el número de contactos telefónicos variará según la duración de la pauta de tratamiento y según valoración: T0 (día 1), 1r contacto (entrevista y cuestionario); T1 (día 10)- TX, contactos periódicos según pauta de tratamiento; TT (día), cierre del caso, cuestionario; Tfinal, cuestionario 6 meses posterior al cierre del caso.

ANEXO 6: INFORME DEL COMITÉ ÉTICO DE INVESTIGACIÓN CLÍNICA

Dra. ANA MARÍA GARCÍA GARCÍA
Universitat Pompeu Fabra
Centre d'Investigació en Salut Laboral (CiSAL)
Doctor Aiguader, 88
08003 Barcelona

Benvolguda Dra. GARCÍA,

El CEIC - Parc de Salut Mar una vegada avaluats el projecte de recerca núm. 2014/5714/I titulat *"Evaluación de una intervención multifactorial en el lugar de trabajo para la prevención de trastornos musculoesqueléticos en los trabajadores (INTEVAL_Spain)"*, li comunica que ha obtingut l'aprovació. Li adjuntem el certificat corresponent.

Cordialment,

COMITÈ ÈTIC D'INVESTIGACIÓ CLÍNICA
CEIC - PARC DE SALUT MAR

Mª Teresa Navarra Alcrudo
Secretaria CEIC - Parc de Salut Mar

Barcelona, a 10 de juliol de 2014

CEIC – Parc de Salut MAR
Dr. Aiguader, 88 I 08003 Barcelona I Telèfon 93 316 06 77 I Fax 93 316 06 36
ceic-psmar@imim.es | www.parcdesalutmar.cat

Parc de Salut Barcelona MAR

Informe del Comité Ético de Investigación Clínica

Doña Mª Teresa Navarra Alcrudo Secretaria del Comité Ético de Investigación Clínica del Parc de Salut Mar

CERTIFICA

Que éste Comité ha evaluado el proyecto de investigación clínica nº 2014/5714/I titulado *"Evaluación de una intervención multifactorial en el lugar de trabajo para la prevención de trastornos musculoesqueléticos en los trabajadores (INTEVAL_Spain)"*, propuesto por la Dra. ANA MARÍA GARCÍA GARCÍA, de la Universitat Pompeu Fabra de Barcelona.

Y que considera que:

Se cumplen los requisitos necesarios de idoneidad del protocolo en relación con los objetivos del estudio y están justificados los riesgos y molestias previsibles para el sujeto.

La capacidad del investigador y los medios disponibles son apropiados para llevar a cabo el estudio.

El alcance de las compensaciones económicas que se solicitan están plenamente justificadas.

Y que éste Comité acepta que dicho proyecto de investigación sea realizado en la Universitat Pompeu Fabra por la Dra. ANA MARÍA GARCÍA GARCÍA, como investigador principal tal como recoge el ACTA de la reunión del día 8 de Julio de 2014.

Lo que firmo en Barcelona, a 10 de Julio de 2014

COMITÈ ÈTIC D'INVESTIGACIÓ CLÍNICA
CEIC - PARC DE SALUT MAR

Firmado: ....
Doña Mª Teresa Navarra Alcrudo

CEIC – Parc de Salut MAR
Dr. Aiguader, 88 | 08003 Barcelona | Telèfon 93 316 06 77 | Fax 93 316 06 36
ceic-psmar@imim.es | www.parcdesalutmar.cat
