# Supplementary material for: Multifaceted intervention for the prevention and management of musculoskeletal pain in nursing staff: Results of a cluster randomized controlled trial
Source: PLoS One. 2019 Nov 18;14(11):e0225198. doi: 10.1371/journal.pone.0225198 (PMC6860418; doi:10.1371/journal.pone.0225198)
Supplement: S3 File — Evaluation of a multifaceted intervention to reduce and manage musculoskeletal pain in nurses (INTEVAL_Spain). (PDF) [file pone.0225198.s003.pdf]

# Evaluation of a multifaceted intervention to reduce and manage musculoskeletal pain in nurses (INTEVAL_Spain)

## FIS PI14/01959

## STUDY PROTOCOL (internal document)

Barcelona, May 2016

PI14/01959

# CONTENT

Justification……… 5
Conceptual frame……….5
Hypotheses and objectives……….10
Study design……….12
Inteval intervention……….15

- Participatory ergonomics……….16
- Case management……….19
- Health promotion……….22
- Integration of the components of the inteval intervention……….23
- Usual health care of occupational health service……….23

Data collection……….23
Evaluation of the intervention……….25

- Efectiveness evaluation……….26
- Process evaluation……….27
- Economic evaluation……….29
- Statistical analyses……….29

Ethical requirements……….30
Strengths and limitations……….31
Bibliography……….33
Annexes……….38
Annexe 1: ficha informativa del proyecto inteval_spain y consentimiento informado……….39
Annexe 2: cuestionario basal……….42
Annexe 3: procedimiento para la aplicación del método ergopar……….60

- Model d'acord del comitè de seguretat i salut per a l'aplicació del mètode ergopar……….64
- Curs ergonomia en el ámbito sanitario y método ergopar……….67
- Presentación "ergonomia laboral"……….70
- Hoja de registro de problemas……….83
- Rol y participación de los/las referentes de las unidades/clústers……….84
- Horas de formación profesional por el curso de ergonomia participativa……….85
- Consentimiento informado……….88
- Full de registre possibles solucions……….89
- Informe propuestas medidas de mejora……….90
- Taula de planificació (formato excel)……….91

Annexe 4: cuestionario gestión de casos……….92
Annexe 5: pauta para el cierre de casos y periodicidad del seguimiento motivacional……….100
Annexe 6: informe del comité ético de investigación clínica……….101

## INTEVAL_Spain PROJECT TEAM

### Research team:

- Consol Serra (PI, coordination), CiSAL-University Pompeu Fabra/IMIM-Hospital del Mar Research Institute; Occupational Health Service, Parc de Salut Mar; CIBER of Epidemiology and Public Health. Barcelona.
- Pilar Peña, Occupational Health Service, Corporació Sanitaria Parc Taulí. Sabadell (Barcelona).
- José M Ramada. CiSAL-University Pompeu Fabra/IMIM-Hospital del Mar Research Institute; Occupational Health Service, Parc de Salut Mar; CIBER of Epidemiology and Public Health. Barcelona.
- Antoni Merelles, Nursing and Podotry department, University of Valencia. Valencia.
- Ana María Garcia, Public Health Department, University of Valencia. Valencia.
- Sergio Vargas-Prada, CiSAL-University Pompeu Fabra/IMIM-Hospital del Mar Research Institute; CIBER of Epidemiology and Public Health. Barcelona.Barcelona.
- Mercè Soler. CiSAL-University Pompeu Fabra/IMIM-Hospital del Mar Research Institute; CIBER of Epidemiology and Public Health. Barcelona.

### Collaborators:

- Chelo Sancho, Occupational Health Service, Parc de Salut Mar MAR; IMIM-Hospital del Mar Research Institute. Barcelona.
- Anna Amat. CiSAL-University Pompeu Fabra; IMIM-Hospital del Mar Research Institute. Barcelona.
- Olga Martinez. CiSAL-University Pompeu Fabra; IMIM-Hospital del Mar Research Institute. Barcelona.
- Gemma Salvador. Agencia de Salut Pública de Catalunya. Generalitat de Catalunya. Barcelona.
- Antonio Brieba. Walkim Barcelona.
- Georgina Badosa y Mònica Astals, mindfulness instructors. Parc de Salut Mar. Barcelona.

### Consultants:

- Pere Boix, David Coggon, Ewan MacDonald, Rosana Cortés, Fernando G. Benavides.

## JUSTIFICATION

The musculoskeletal conditions include more than 150 diagnoses that affect the locomotor system, ranging from acute and short-term conditions, such as fractures, sprains and strains, to chronic conditions associated with pain and disability. The most common feature of musculoskeletal conditions is pain, which can reduce the ability of people to work and participate in social roles, and can have an impact on mental health and well-being, and more widely in society.

The prevalence of musculoskeletal pain (MSP) is very high and is the main cause of sickness absence in health workers, especially nurses and nursing assistants who work in hospitals [1], due to the high exposure to ergonomic risk factors in their tasks, such as in the mobilization of patients [2]. In Spain, as in other European countries, all employers have to organize some type of prevention service according to the exposure to occupational risks and the size of the company. The tasks of these prevention services include risk assessment, investigation of occupational accidents, health surveillance through health examinations, and prevention, training and information covering occupational and non-occupational risks. External Prevention Services are usually the main occupational health provider for small and medium enterprises, while large companies, including hospitals, usually have a Prevention Service or Occupational Health Service (OHS), which offers better opportunities to investigate and test new approaches to improve workers' health.

## CONCEPTUAL FRAME

Complex and dynamic interactions between biological, psychological and social factors (including environmental and cultural factors) have a strong influence on the occurrence and recurrence of musculoskeletal disorders (MSDs) and the related sickness absence [3]. Although the biopsychosocial model [4] has become the dominant framework through which the etiology and prognosis of MSDs is conceptualized, its translation into practice has not been optimal [5]. Traditionally, the causes of MSDs have been investigated through the disciplines of biomechanics, physiology, genetics, epidemiology and rehabilitation. Usually, each of these disciplines has studied the development of MSDs in isolation from the other disciplines [6]. However, this fragmented approach does not offer the optimal prevention and management of MSDs. Regarding to the MSDs that affect work functioning and the

occurrence of sickness absence, the determinants and interactions involved in the causal pathways are even more complex (Figure 1).

**Figura 1.** Conceptual model for the causation of musculoskeletal disorders in workers affecting their capability to work and/or determining sickness absence (modified from National Research Council, 1999).

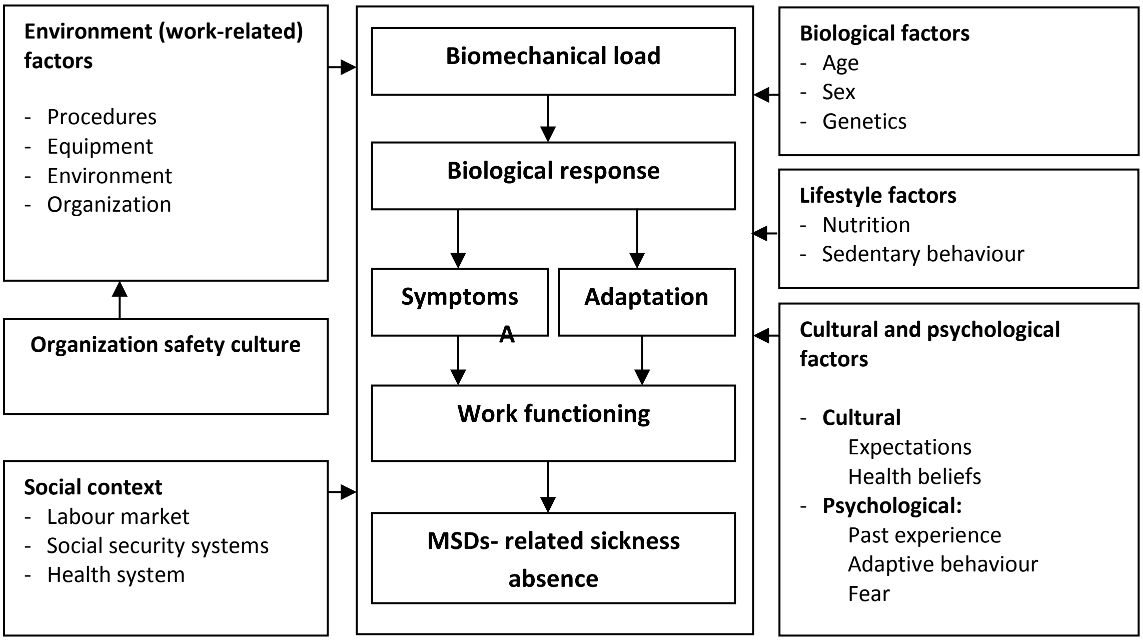


As shown in Figure 1, there several conditions are related to the work environment, tasks and organization that have been linked to the appearance of MSDs that affect different parts of the body. Manipulating objects, applying traction or pushing forces to tools or machines, uncomfortable postures or postural demands, such as twisting or bending the trunk, working in confined spaces, repetitiveness or the use of vibration tools are examples of working conditions that have been associated consistently with MSDs in the neck-shoulder region and in the hand-wrist region [7,8]. But evidence has also accumulated that links the occurrence of MSDs with work-related stressors [9]. A recent review and meta-analysis supported the conclusion that work-related psychosocial factors should be considered independent predictors of the development and persistence of MSDs, and are relevant to prevention and intervention programs in occupational health and safety [10]. For years, the causal mechanisms of this relationship were poorly understood and some speculated that poor psychosocial environments would create a situation in which workers

would be more likely to report injuries and illnesses. However, experimental research has been able to demonstrate a complex biomechanical pathway through which psychosocial risk factors have a direct role in the etiologic pathways that cause MSDs [6].

The term preventive culture in industrial organizations was first introduced by Zohar in the 1980s, and since then it has developed fruitfully in relation to occupational health and safety practices and company-level results [11-13]. A systematic review of the relationship between indicators of organizational preventive culture (defined as "collective perceptions of employees with respect to the organization's commitment to safety") and health outcomes in health workers, found a greater frequency of symptoms and MSDs in nurses working in health units and services with worse indicators of organizational preventive culture [14]. In addition, a relationship has been described between the incidence and duration of sickness absence and the employment situation, labor relations and social protection systems prevailing in countries and periods [15, 16], with marked variations, such as during periods of economic crisis [17].

In addition, there are well-established differences in the incidence and duration of MSDs according to age and sex, associated with physical, anthropometric and hormonal differences, but also probably due to different exposure profiles by sex and age to the other determinants.

Finally, focus on secondary and tertiary prevention, there is a growing interest in the cultural and psychological factors described in Figure 1, which are increasingly recognized as the main determinants of the appearance and consequences of MSDs. Although observational studies have systematically linked MSDs with certain work activities, standard ergonomic interventions in the workplace have often failed to reduce the related sickness absence [18]. Is posible that the benefits of a reduced mechanical load are counteracted by adverse impacts on health beliefs and expectations. Among patients suffering MSP, those who expect the symptom to persist and/or who do not remain active, have worse results, even when other prognostic variables are taken into account [19]. Other health beliefs, which are partly determined by culture, may also predispose to the onset or persistence of symptoms and associated sickness absence, and may contribute to the large differences observed in the prevalence of disabling MSP between countries and within of the countries over time [20]. There is also evidence showing that the tendency to somatize (ie, experience

distress from common somatic symptoms) and low mood are associated and predict MSP [21].

Based on the conceptual model described above and summarized in Figure 1, INTEVAL_Spain has been designed to evaluate a multi-component workplace intervention that comprises three main components: a participatory process to change and improve ergonomic conditions in the workplace, directed management of workers with personnel with MSDs, and a health promotion program. Research on interventions in the workplace that integrates the bio-psychosocial model and the conceptual framework of the pathways and factors that contribute to MSDs is almost non-existent, except for a Danish randomized, multifaceted and multifaceted controlled trial [22] that combined participative ergonomics, cognitive behavioral training and physical training with the aim of preventing low back pain and its consequences among nursing assistants.

Figure 2 shows our conceptual model in relation to the opportunities of INTEVAL _Spain to evaluate the impact of different factors on the occurrence and consequences of MSDs.

**Figura 2.** Focus of INTEVAL multi-country multi-component intervention to control and prevent musculoskeletal disorders in workers.

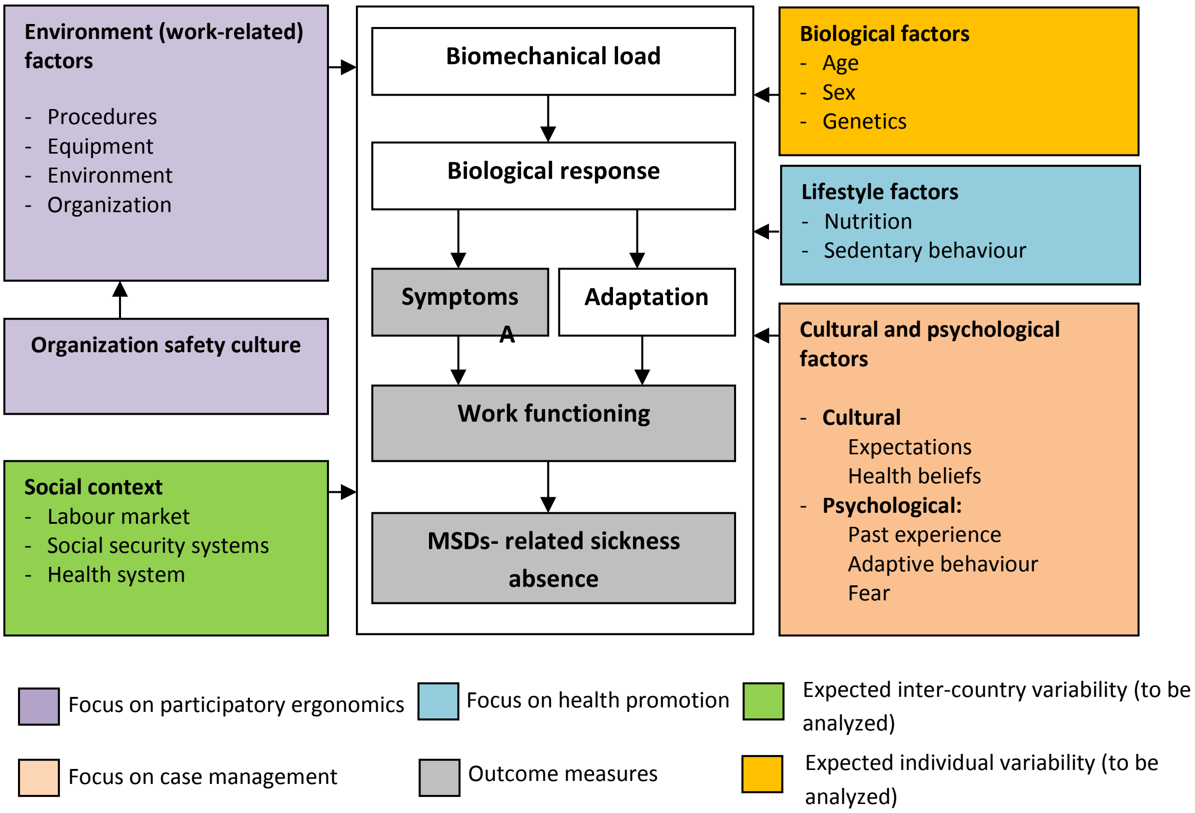

The INTEVAL_Spain project is derived from the following hypotheses: (a) the bio-psychosocial model is the appropriate framework through which the etiology and prognosis of MSDs should be conceptualized; (b) a participatory "problem-solving process" based both on management support and on worker participation is a determining factor for successful health and safety practices aimed at preventing workplace MSDs; (c) the specific management of cases of workers with functionally limiting MSDs will favor remaining at work or returning to it beforehand; (d) a combination of primary, secondary and tertiary prevention (including hazard control, behavioral and cultural changes, early diagnosis and adequate follow-up of cases) is necessary for effective control of work disability related to MSDs; (e) the resources available for health and safety in European companies, regardless of their activity, size or location, can be used more efficiently to prevent MSDs related to work and to reduce the consequences of MSDs on employees.

The biopsychosocial model simultaneously considers the biological, psychological and social factors that can impact on health and general well-being, linking the three factors, and has been recognized and accepted by the WHO as established in the international classification of functioning, disability and health [23]. Interventions based on the biopsychosocial approach have been used in the management of temporary disabilities [24] and in vocational rehabilitation initiatives [25]. From the results of mostly isolated interventions, the evidence suggests that a combination of several specific approaches could give a perspective of the best results [26].

It has been observed that the ergonomic interventions with greater probability of success are those that involve the company's organizational preventive culture and with a high commitment of the interested parties [27]. Ergonomic improvements in working conditions can produce greater benefits if they are participatory [28], that is, the employees actively participate in the identification of work modifications that reduce the physical and psychosocial burden and can make their work easier and confortable. A systematic review supports the effectiveness of participatory ergonomic interventions to reduce pain, episodes of sickness absence and lost workdays due to MSDs [29]. In addition, there are indications that this type of intervention can be cost-benefit [30]. In addition, there is evidence that there are additional benefits of participatory interventions in the workplace, including improvements in the organizational preventive culture and associated attitudes and behaviors, provided that the type of participation has been previously well defined and organized in accordance with the specific context of each one [31].

In addition, the scientific literature provides several examples of successful case management programs for workers and people of working age with MSDs [25, 32]. Case management is a collaborative process based on evidence that assesses, plans, implements, coordinates, monitors and evaluates the options and services required to meet the needs of health, welfare, education and employment of a person, using communication and the resources available to promote quality and cost-effective results [33]. This process involves a summ of directed and individualized steps that help and support the recovery of workers with MSDs (or any other condition) towards an early and safe return to their best possible functional and work capacities. The available evidence suggests that case management interventions reduce the duration of sickness absence, the symptoms of MSDs and the sickness absence related, and improve job retention [25, 32, 34]. It has also been suggested that these types of programs can be profitable [34].

The integration of activities on healthy lifestyles at work is also an important component of occupational health programs to reduce MSDs [35], which will also include different strategies that promote physical activity of workers, well-being emotional and healthy diet [36-39]. As for healthcare workers, the promotion of healthy lifestyles should be a priority because of its double impact on their own health and that of patients, fostering these lifestyles in the general population.

The project INTEVAL_Spain is in the spectrum of "theory to practice" (theory to application). In this sense, the participation of key stakeholders in the workplace in the development and implementation of the intervention could be considered as a first activity of transfer of "know-how", which will benefit the participating companies. We are aware of the enormous opportunities for the transfer of knowledge within the INTEVAL_Spain Project, supporting what has been called "evidence-based health and safety at work" [40].

## HYPOTHESES AND OBJECTIVES

### Hypothesis

An intervention in the workplace that combines: primary prevention through a) participatory ergonomics to identify and control conditions and work situations associated with the appearance of MSDs in workers (primary prevention), and b) a program to promote health based on the promotion of healthy eating habits through the Mediterranean Diet;

and secondary and tertiary prevention through c) personalized program of case management for the early diagnosis and personalized management of workers affected by functionally limiting MSDs (secondary prevention) and by the adaptation and facilitation of the return to work of workers in sickness absence due to MSDs (tertiary prevention); it will have a positive impact in terms of a reduction of at least 20% in the prevalence of MSP, as well as incidence and duration of associated sickness absence and in terms of cost-effectiveness and cost-benefit.

## Objectives

The main objective of the INTEVAL_Spain Project is to evaluate a multifaceted intervention formed by three different components and encompassing the three levels of prevention to prevent and manage the MSP in the nursing staff.

The specific objectives are:

1. Analyze the effectiveness of the intervention in terms of health measured with the reduction of MSP, reduction of the incidence and duration of sickness absence, and improvement of the work functioning.
2. Analyze the process of the intervention based on the indicators of context, recruitment, scope, supply dose, received dose, fidelity, adherence and satisfaction; and through qualitative information, collected in semi-structured interviews.
3. Analyze the cost-effectiveness and cost-utility of the intervention from the perspective of society and health system, measuring the effectiveness in terms of MSP and quality of life.
4. Analyze the cost-benefit of the intervention from the perspective of the hospital, measuring the benefits in terms of MSP.

## STUDY DESIGN

Cluster Randomized Controlled Trial (CRCT) with two arms: an intervention group and a late intervention control group, where the clusters are independent hospital units and the participants are nurses highly exposed to risk factors ergonomic at work.

### Scope, selection of companies and calculation of the simple

The companies participating in the trial must meet four conditions that are considered relevant to the objectives and feasibility of the study:

a) Size (at least 500 workers).
b) Commitment and explicit interest on the part of the managers of the company to carry out the intervention and its evaluation.
c) Existence to the company of a Prevention Service that maintains routine quality records in occupational health, and also interested in developing the intervention.
d) Existence of work units with exposure to significant musculoskeletal risks, based on the information available from the Prevention Service.

Based on these criteria, two tertiary hospitals in Barcelona city and Sabadell (province of Barcelona) have been selected with a similar level of care complexity, with a work population of about 3,500 workers each, of which around 60% are staff of nursing, and with a Prevention Service of their own.

Table 1 shows the companies participating in the project.

**Table 1.** Description of the companies participating in the INTEVAL_Spain Project.

| **Company** | **Sector** | **Description of company activities** | **Number of workers (November 2015)** |
|---|---|---|---|
| Parc de Salut MAR (PSMAR) | Third level hospital | Centers of specialized attention of acute, psychiatry and sociosanitary; primary care. | 3,730 |
| Corporació Sanitària Parc Taulí (CSPT) | Third level hospital | Centers of specialized attention of acute, psychiatry and sociosanitary; primary care. | 3,817 |
| **Total** | | | **7,547** |

The estimates of the sample size for the study were carried out according to the following criteria:

a) Prevalence of back pain of occupational origin (main MSD related to work), according to data available in the VII National Survey of Working Conditions [1], estimated at 80% for health workers.

b) Expected impact of the intervention, estimated in terms of prevalence of MSP and based on results observed in previous studies [40, 41], having been established in the hypothesis in a reduction of 20%.

c) Alpha values (type I error) = 12: 05, statistical power = 0.80, ICC intraclass correlation coefficient = 0.05.

According to these criteria, and applying the orders of Stata Sampsi and sampclus, the participation of 294 subjects is required. The minimum sample size of 300 subjects distributed as follows was agreed: 150 in the intervention group and 150 in the control group.

The units of the companies participating in the study have a variable number of between 20 and 60 workers. Therefore, according to the information available, the total number of workers included in the study would vary between 160 and 480 subjects.

### Selection of clusters and randomization

Nursing staff (nurses and assistants) will be selected from hospital units with high musculoskeletal risks due to exposure to ergonomic factors in the workplace and the type of patients (medium and high dependency), according to the information available and knowledge of the prevention technicians of the prevention service of the participating companies.

Eigth distributed clusters were agreed as follows (table 2):

- Parc de Salut Mar: 3 intervention + 3 control
- Corporació Sanitaria Parc Taulí: 1 intervention + 1 control

The distribution of the units to the intervention and control groups is done by simple randomization stratified by center.

**Table 2.** Units/clusters and number of workers.

| Company | Units/Clusters | Number of workers |
|---|---|---|
| Parc de Salut MAR (PSMAR) | Surgical unit | 106 |
| | ICU: intensive care unit | 87 |
| | UH04: cardiology and pulmonology | 43 |
| | UH30: traumatology and nephrology | 44 |
| | Llevants 3- 4: psychogeriatrics | 48 |
| | AGU: acute geriatric unit | 36 |
| Corporació Sanitària Parc Taulí (CSPT) | UH06: traumatology and nephrology | 57 |
| | UH08: neurology | 52 |
| **Total** | | **473** |

## Criteria for eligibility and recruitment of participants

The study subjects are the nursing professionals (nurses and assistants), including employees with a sickness absence, of the units/clusters included and who voluntarily accept to participate.

Exclusion criteria are:

a) Have a temporary contract of less than 3 months that is not renewable in time.
b) Work in several different units.
c) Be on a sabbatical leave.

In order to recruit the participants, on-site information sessions will be organized in the units and all work shifts (morning, afternoon and evening), jointly with the delivery and collection of the informed consents and the baseline questionnaires:

a) Initial informative sessions: before communicating the units that will be intervention or control group, information sessions will be held in each unit and covering the different shifts. These sessions will last between 30 minutes and one hour, and in them the Champion together with the person of the research team responsible for each hospital center will explain that the unit has been selected to participate in a research study, in which they can be intervention or control group and that the main data will be collected mainly from questionnaires.

b) Compliance and delivery of the informed consents (Annex 1) and baseline questionnaires (Annex 2): at the same meeting the informed consents and the baseline questionnaires will be delivered. These can be completed at the time or in a maximum period of two weeks, to facilitate greater participation. A space will be enabled in the control of each unit where consents and questionnaires will be placed to complete, as well as for the workers to deliver their already completed. The Champion of the project will check periodically if the empty questionnaires remain and to pick up the completed ones.
c) After filling out and returning these documents, the units will be randomized and their status as intervention or control groups will be informed.
d) In the intervention units another informative session will be held, between 30 minutes and one hour, and in this the Champion together with the person of the research team responsible for each hospital will present the intervention and the different activities to be developed.

**Blinding**

This intervention is not blinded:

- At the patient level: the condition of inclusion in the intervention or the control group can not be blinded, although the clusters will be randomized after signing the informed consent and completing the baseline questionnaire.
- At the professional level: the services provided and the participating OHS professionals can not be blinded since they are involved in the implementation of the intervention.
- At the level of analysis: the researcher who will perform the analyzes will also be in charge of creating and cleaning the database, so he will be aware of the intervention and control units in order to categorize them.

## INTEVAL INTERVENTION

The INTEVAL intervention covers the three levels of prevention (primary, secondary and tertiary prevention), lasts one year and is subsequently implemented in the control group.

It consists of 3 components: participatory ergonomics, case management and a health promotion program (Figure 3).

**Figure 3.** Algorithm of the INTEVAL intervention.

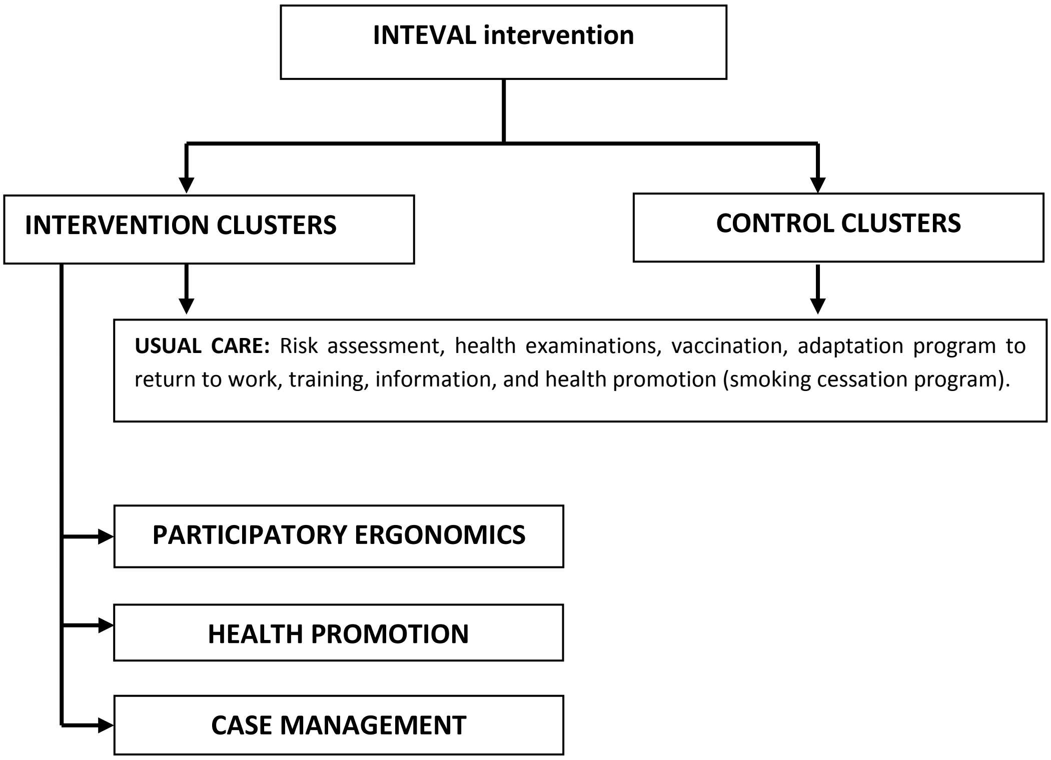


- **Participatory ergonomics**

The ERGOPAR methodology will be followed, consisting of a standardized procedure of participatory ergonomics at work that has been developed and piloted [42, 43]. ERGOPAR involves the direct participation of workers in the identification and solution of ergonomic problems related to work, taking a step beyond the more traditional ergonomic practices based largely on technical measures, changes in the environment, and the provision of information and training for workers.

The team of participatory ergonomics will plan the process under the supervision of the coordinator (Champion), who will act as leader and facilitator of the intervention. The Champion will organize and direct the work to be developed. This can be an ergonomist, but it could also be another professional with experience in leading work groups and/or carrying out participatory interventions in the workplace.

Methodology (Figure 4 and Annex 3):

1. **Preparation and constitution phase of the ERGO working group for each unit/group of intervention, which will include:**
    a) Coordinator (Champion).
    b) Prevention technicians.
    c) Supervisors of the unit/group of intervention.
    d) Referents: workers of the intervention work unit, in general 1 for each work shift (morning, afternoon, night A and night B), volunteers and with functions of invigoration of the intervention in each unit. They will be given a document with their functions, they will sign an informed consent and as compensation their participation will be recognized as 20 hours of continuous training.
    e) A Prevention Delegate.

Functions: The ERGO group is responsible for the development and implementation of the intervention and its members must receive basic training on ergonomics and participatory methods. The intervention of participatory ergonomics involves the direct participation of workers in the identification and solution of ergonomic problems related to work, taking a step beyond the more traditional ergonomic practices based largely on technical measurements, environmental changes, and the provision of information and training to workers [44]. As part of the Project, the Ergo team will be established in each company where the intervention is developed.

The ERGO group will hold 3 meetings of 1 hour each:

- Meeting 1: training of the ERGO group by the prevention technician.
- Meeting 2: identification of ergonomic problems in the unit and its prioritization
- Meeting 3: identification of improvement/preventive measures.

The participants to each meeting will sign an attendance sheet that will tell them in turn as ongoing training.

At this point, a signed agreement with the participating companies (Top management and Health and Safety Committee) will be necessary to guarantee the correct execution of the intervention.

2. **Diagnostic phase:**

Consists in the distribution to the workers of the intervention work unit of a self-completed questionnaire previously validated [42], in which data on musculoskeletal damage and exposure to risk factors for MSDs at work are collected (see below in "Data Collect").

This information is later analyzed and discussed by the components of the ERGO group in order to identify risk factors and key points for improving working conditions, and their prioritization.

3. **Treatment phase and prevention circles:**

The treatment phase consists of the fact that the information collected in the questionnaires is shared and discussed with the workers of the intervention unit in prevention circles, formed by all the workers of each unit.

The prevention circles agree and propose, in informal meetings energized by the referents, a prioritized list of measures to improve working conditions to avoid or reduce the ergonomic problems identified. The final list is agreed at the meeting of the ERGO group. These measures may include structural, technical, organizational, training/information improvements and in the workplace.

4. **Implementation of the proposed improvement measures, operating group:**

The proposal for improvements in phase 3 is transferred to the OHS, the Health and Safety Committee (HSC), and those responsible for its implementation in the company. It has been estimated between six and twelve months the time required to complete all phases of the intervention in each work unit [43], although it depends on other variables (organizational preventive culture in the company, complexity of the measures and of the company itself, etc.).

The Operative group is dynamic and consists of those responsible for the implementation of the proposed measures, and is coordinated and energized by the Prevention Service.

As a tool for monitoring the implementation of improvement proposals, the operating group will use the planning table, according to the model in Excel designed for the study.

In coordination meetings between the HSC, the Directorate and the OHS, Ergopar follow-up will be included in the permanent agenda of the meetings.

**Figure 4.** Participatory ergonomics component, ERGOPAR method.

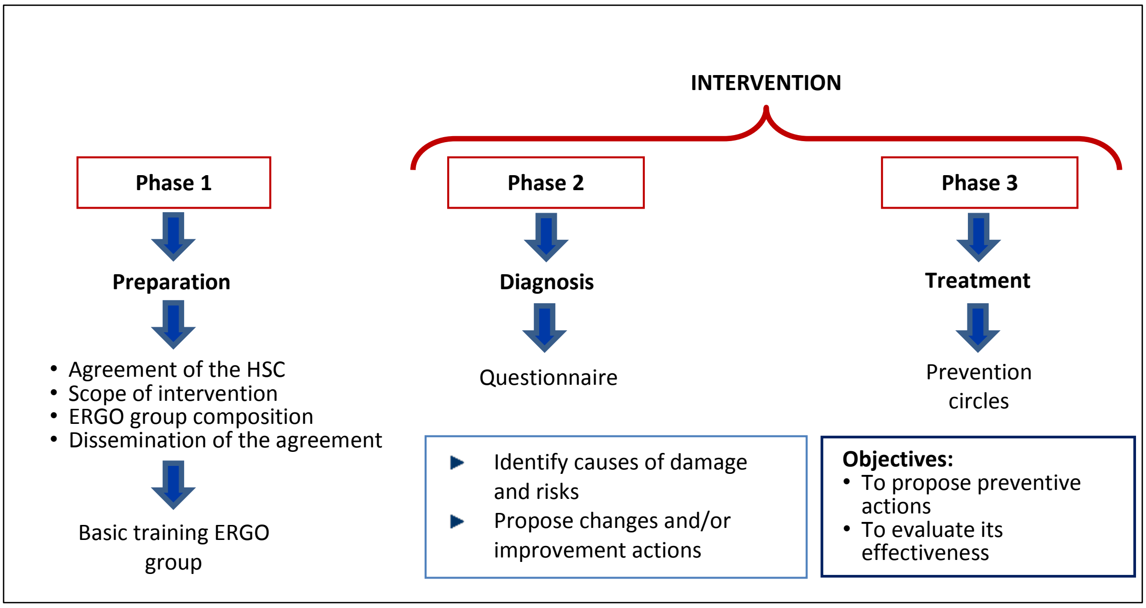


- ## Case Management

The case management component consists of the individual approach of workers with a limiting MSD, with the aim of contributing to the recovery of workers with MSDs and, in those with sickness absence, returning to work in adequate health conditions. Services derived from the case management will be organized by the hospitals themselves and using their own researchers.

Case management is defined by the Case Management Society UK as a collaborative process that values, plans, implements, coordinates, monitors and evaluates the options and services required to cover individual health, assistance, educational and employment needs, using the communication and available resources to promote quality and cost-effective results [32].

In the context of the INTEVAL intervention, case management is also understood as a multidisciplinary service and applied to the conceptual framework of the biopsychosocial model of health to manage in all stages of the disease, in our case the MSDs, as well as measures based on the evidence to achieve vocational rehabilitation. It will focus on the work and maintenance of the workers in their jobs and the improvement of the functional capacity to work. It will involve mainly telephone contact with affected workers in combination with personal interviews.

In this intervention there will be a Case Manager, who will be trained based on the objectives and components of this intervention. It is not necessary to have a health profile, however, it is recommended that you have an empathetic but robust personality to coordinate and manage the intervention process. It will provide guidance and support in the referral, evaluation and management of cases.

Methodology (Figure 5)

Workers with some musculoskeletal condition can voluntarily opt for case management:

a) By themselves.
b) At the proposal a supervisor.
c) At the proposal of the occupational practitioner of the OHS.

Workers with underlying organic pathology (red flag) are excluded from this intervention component and managed according to standard medical practice.

A qualified case manager (Vocational Rehabilitation Case Management course, Healthy Working Lives, University of Glasgow) assigns participants to three strata of management and treatment, according to their risk level of persistent musculoskeletal symptoms: low, medium or high. This profile is obtained in a telephone interview through a questionnaire that includes validated tools to generate a risk profile that assesses the presence of pain, comorbidity, limitations to carry out daily activities, fear, beliefs and negative expectations regarding the prognosis of pain, to the presence of anxiety and other mood disorders [45-51] (Annex 4).

- Workers assigned to the low risk group attend an educational session about beliefs related to health and pain.
- Workers assigned to medium and high risk groups receive a specific and personalized treatment that includes rehabilitation, physiotherapy and cognitive-behavioral therapy.

Each case will be studied separately and the specific will be adapted to each worker. However, also as guidance, a guideline is established to determine the closure of cases and the periodicity of motivational follow-ups (Annex 5).

**Figure 5.** Algorithm of Case Management

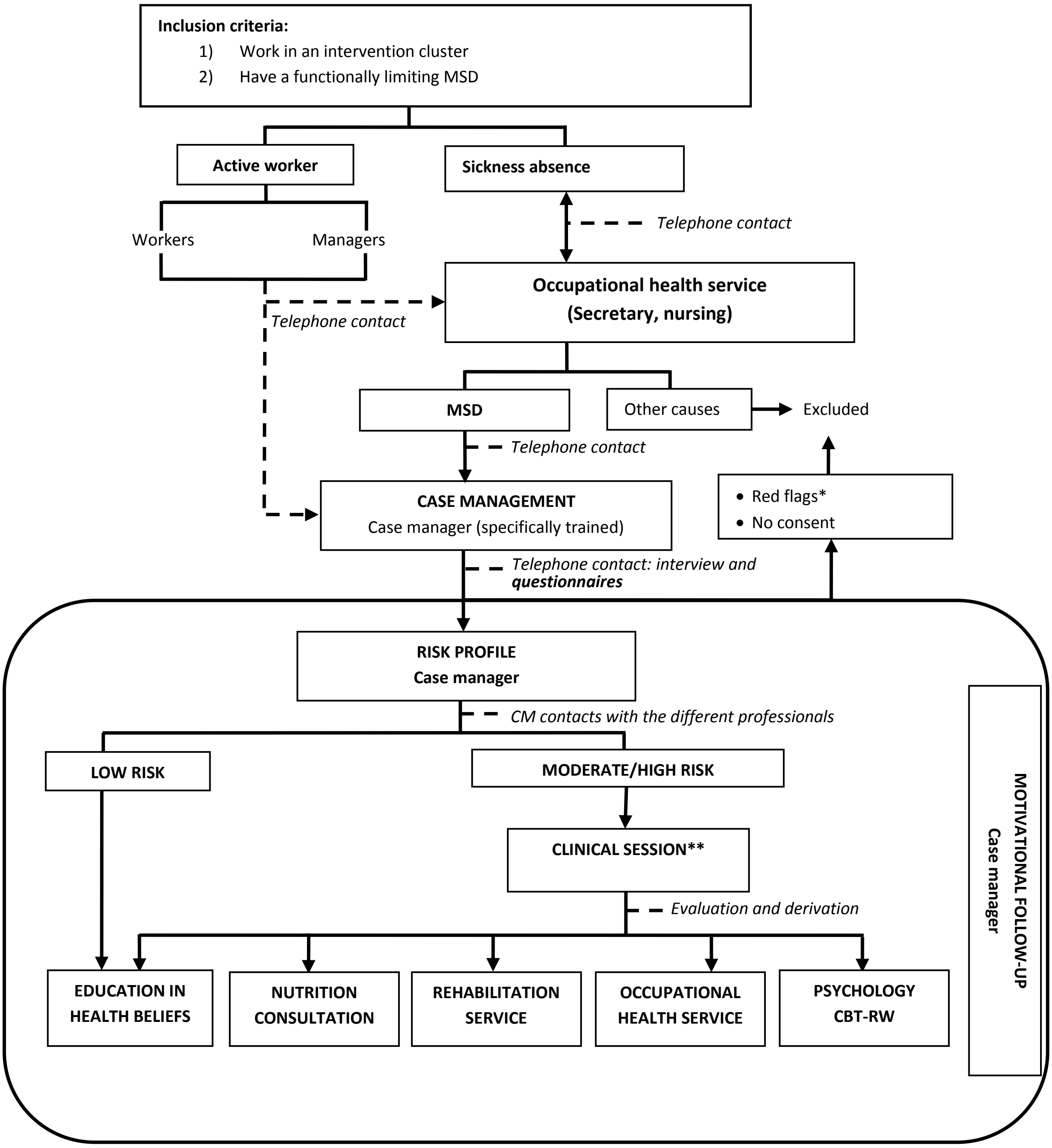


* Red Flags: Presence of warning signs (severe trauma or minor trauma in patients with osteoporosis, constitutional symptoms: fever, weight loss, malaise), pregnancy or puerperium, morbid obesity (BMI ≥ 40), recent surgery (< 3 months), neurodegenerative disease, decompensated psychiatric pathology, decompensated cardiac or respiratory pathology, osteoporosis with symptomatic vertebral fractures.

** It will be derived to the different services based on identified needs.

In parallel, cases may be presented to the weekly clinical session with members of the OHS to assess possible specific needs at work, such as adjustments or improvements in the work to help workers to stay and/or return to work soon.

The case manager will contact the worker by phone periodically to carry out a motivational follow-up, in addition to coordinating the services and planning the sessions.

- **Health Promotion**

A health promotion program offered to all nurses and nursing assistants of the participating intervention units will be created and the application will be voluntary and free. Will include:

a) Mindfulness training: defined as a self-regulation approach for stress reduction and emotional management [29], and will consist of an adapted course of 4 sessions of 2 hours each in the stress reduction training (MBRS), which has been shown to be effective in healthcare workers [52].
b) Nordic Walking Training: defined as a walking technique that uses specially designed sticks to actively involve the upper body and arms with ample scientific evidence of its benefits on various health outcomes, including the MSDs [36], which It will consist of a training program of 12 sessions of 1.5 hours/session for 12 weeks.
c) Healthy diet based on the Mediterranean diet: one of the healthiest dietary models that currently exist [37, 38] divided into two main activities:
    a. Healthy nutrition face-to-face session: session carried out by a nutritionist/chef to promote adherence to the Mediterranean diet and healthy eating habits. Estimated time: 2-3h.
    b. Web platform: healthy recipes will be shared on a regular basis (2 recipes per month) supervised by a nutritionist and coordinated by the Champion. In addition, it will be completed with tips for a healthy lifestyle and a discussion forum.

On-site briefings will be organized in the units to present each of these activities to the participants, by units and work shifts.

- **Integration of the components of the Inteval intervention**

All the components of the intervention are integrated and require the coordination of a Champion who acts as a leader and facilitator of the intervention, organizing and directing the work that the study team must develop.

Their tasks include:

- Group motivation.
- Communication between the research team and the participants.
- Planning of meetings (briefings and meetings of research teams).
- Organization of health promotion activities (calendar planning).
- Collection and processing of data.
- Writing of reports.

- **Usual health care of Occupational Health Service**

During the intervention period, the OHS of each participating hospital will continue to provide standard occupational health practices for both intervention units and control units. These practices include:

a) Occupational risk assessments.
b) The investigation of work accidents.
c) Health surveillance.
d) The cessation of smoking.
e) Training and information.
f) The evaluation of experts in occupational health at all levels of the hospital (managers, supervisors, workers).
g) The usual support program for return to work, mainly to interventions related to workplace adaptations, clinical support and management of permanent disability.

## DATA COLLECTION

The instruments for collecting data will be collected through three sources:

a) Questionnaires administered to the participants.
b) Existing records in the participating companies.

c) Qualitative interviews.

**Questionnaires**

A part of the data will be collected through standardized and validated questionnaires.

For the evaluation of the effectiveness of the intervention, these questionnaires will be administered at the initial moment, follow-up of six and twelve months (see section "Evaluation of the intervention", in paper format, and the Champion of the project will process them in the online registration.

To ensure a correct registration, general instructions are given:

- If any of the questions in a single answer has more than one box checked, it is canceled and both must be left blank (missing).
- Always use a comma (,) instead of a period (.) When decimals are placed.
- Fill open questions always with a lowercase letter, including the first letter.

A sample of 1% of questionnaires will be selected and its processing will be checked to detect possible inconsistencies and/or identify errors. All questionnaires will be reviewed when 10% or more errors are detected.

The questionnaires used in the evaluation of the process in relation to case management will be administered by telephone.

**Records**

The data of the following registers will be available:

- Internal registration of the Champion: it will store the data referring to the process indicators, and the costs of the intervention.
- Data will be drawn mainly from two sources: the Champion's internal record, and the hospital records (Prevention Service, HR, and analytical accounting department).
- Hospital records: data on sickness absence (OHS and HR), data on the cost of the disease (Prevention Service and Analytical Accounting Department).

**Qualitative interviews**

Two groups of semi-structured interviews will be developed: one of the research team and the other with a group of study participants from each hospital, in order to provide data for the process evaluation (see process evaluation).

## EVALUATION OF THE INTERVENTION

The evaluation of the intervention will be divided into:

- Evaluation of health outcomes.
- Evaluation of the process of implementation of the intervention.
- Economic evaluation.

Table 3 shows the data collection periods according to the indicators and type of evaluation; and Table 4 presents the tools that make up the INTEVAL questionnaires.

**Table 3.** Evaluation, indicators and data collection periods.

| Evaluation | Indicators | T0 (baseline) | T1 (6 months) | T2 (12 months) | Intervention period |
|---|---|---|---|---|---|
| **Health Outcomes** | MSP prevalence | X | X | X | |
| | Incidence and duration of sickness absence due to MSDs | | | | X |
| | Work functioning | X | X | X | |
| | Organizational preventive culture | X | X | X | |
| **Co-variables** | Age, sex, birth country, shift, contract, seniority in the company and position | X | X | X | |
| **Process** | Recruitment | | | | X |
| | Context | | | X | |
| | Reach | | | | X |
| | Dose delievered | | | | X |
| | Dose received | | | | X |
| | Fidelity | | | | X |
| | Satisfaction | | | X | |
| | Role of stakeholders (implementation strategy) | | | | X |
| **Economic** | Intervention costs | | | | X |
| | Ilness costs | | | | X |
| | MSP prevalence (effectiveness) | X | X | X | |
| | Quality of life (EQ-5D) | X | X | X | |

- **Efectiveness evaluation**

The main outcome variables will be the prevalence of MSP and the sickness absence associated.

As secondary outcome variables, the work functioning and organizational preventive culture will be taken into account.

a) **Prevalence of musculoskeletal pain:** MSP will be measured through the Spanish adaptation of the Nordic questionnaire included in the ERGOPAR method [42]. This tool measures pain in the neck, shoulders and back, back, elbows, hands, legs, knees and feet by asking "Do you have discomfort or pain in this area?" The data are collected at baseline, at six and 12 months.
b) **Sickness absence**: the data of the episodes and the duration of the temporary incapacities due to a musculoskeletal condition are recorded in the HR and OHS records during the study period and up to one year before the intervention.
c) **Secondary outcome variables**
    a. **Work functioning**: it is collected in the baseline questionnaire, six and twelve months, through the Spanish version of the Work Role Functioning Questionnaire (WRFQ-SPV)) [53-55]. This tool is a self-administered questionnaire that measures perceived difficulties at the time of carrying out its work due to health problems [56] and consists of 27 items divided into five sub-domains: work scheduling demands, production demands, demands physical, mental demands and social demands. The score of this questionnaire ranges from 0 to 100, with a maximum score of 100 (having 100% of its functional capacity).
    b. **Organizational preventive culture:** measured through the IWH Organizational Performance Metric (IWH-OPM) [57], a questionnaire based on scientific evidence of eight elements that serves to help organizations assess and improve their health and safety performance and measured in the baseline questionnaire, at six and twelve months.

**Co-variables:** In the questionnaires used and from the routine records of the companies will also be collected, information on age, sex, country of birth, job, type of contract, distribution of working time and seniority in the company and in the position of the workers in the participating units.

**Table 4.** INTEVAL questionnaires: objectives and data collection periods

| INTEVAL Questionnaire and tools | Objective | Q0 baseline | Q1 (6 months) | | Q2 (12 months) | |
|---|---|---|---|---|---|---|
| | | | I | C | I | C |
| Nordic Questionnaire (ERGOPAR Method), adapted to the healthcare sector | Collect sociodemographic variables, assess musculoskeletal disorders through self-perceived pain, and know the work's own postures, as well as the asociated burden. | X | | | | |
| Participation to the activities | Know the participation in the activities developed in the INTEVAL project | | X | | X | |
| Context question (improvements) | Evaluate the improvement in mobilizations, transfers, load handling | | | | X | X |
| Prevention service question | Evaluate the knowledge of the participants regarding the OHS staff | X | X | X | X | X |
| IWH Organizational Performance Metric Questionnaire | Evaluate the preventive culture of the participants | X | X | X | X | X |
| Labour satisfaction | Evaulate the satisfaction | X | X | X | X | X |
| CoPsoQ-istas21 (short version) | Evaluate the psychosocial environment and productivity | X | | | X | X |
| Work Role Functioning Questionnaire (WRFQ-Sv) | Evaluate functional capacity to work | X | X | X | X | X |
| European Questionnaire five dimensions (EQ-5D-3L) | Evaluate the quality of life | X | X | X | X | X |
| Method Ergopar evaluation questions | Evaluate the ERGOPAR method | | | | X | |
| Participation in the case management | Know the participation in case management | | | | X | |

## • Process evaluation

The intervention process will be evaluated using quantitative and qualitative data.

### Quantitative evaluation

It will be based on indicators for process evaluation [58, 59]: recruitment, context, scope, supply dose, received dose, fidelity and satisfaction. Also, we will add the role of the key people (implementation strategy). The quantitative data of the process evaluation will be collected through questionnaires and records of the Champion.

a) **Context:** the questionnaires of the six and twelve months of follow-up will be compiled with three questions related to the aspects that affect their usual workload (improvement of the manual mobilization of the patients, technical aids and load handling).

b) **Recruitment:** refers to the procedures used to approach and attract future participants of the program, and is defined as the proportion of potential workers who sign the informed consent to participate in the study, on the total of possible participants.
c) **Reach:** it will be calculated as the proportion of people who answered the questionnaires (baseline) of those who will sign the informed consent. For each component of the intervention, the scope will be calculated as the proportion of people who will have participated in each one of them.
d) **Dose delivered**: it will be calculated as the number of hours of services offered.
e) **Dose received:** according to the degree to which the participants have actively participated in each component of the intervention.
f) **Fidelity**: it will include the fidelity of the intervention and the adhesion of the participants.
    a. The fidelity of the intervention will be calculated as the proportion between the sessions and/or activities carried out on the total of the planned ones.
    b. The adherence of the participants will be calculated as the ratio between their attendance and the dose offer.
g) **Satisfaction**: it will be collected at the end of each component of the intervention and in the 12-month follow-up questionnaire, through the questions "Have you met your expectations?", and "in general, what is your satisfaction?" scale from 1 to 10, with 10 being the maximum satisfaction.

**Qualitative evaluation**

The qualitative data will be used to identify the key points and possible improvements, as an indicator of implementation strategy and will be collected through semi-structured interviews.

Two groups of semi-structured interviews will be developed: one of the research team and the other with a group of study participants from each hospital. The objective of these groups will be to carry out the evaluation of the internal process to detect the limitations and barriers, in order to make improvements at the organizational level, as well as helping to understand some results.

The points discussed in the interviews will be:

a) Communication
b) Questionnaires (format, distribution, etc.),

c) Components of the intervention:
   a. Participatory ergonomics (meetings, organization, implemented measures and dissemination)
   b. Case management (dissemination and format and quality of the course)
   c. Nordic walking (dissemination and format and quality of the course)
   d. Mindfulness (dissemination and format and quality of the course)
   e. Healthy diet (online website, activity of the chef's session and place and time of the chef's session).

- **Economic evaluation**

A cost-utility and cost-effectiveness analyses will be carried out from the perspectives of the national health system and society; as well as a cost-benefit analysis from the perspective of the hospital.

- Cost-utility analyzes will be carried out to analyze changes in quality-adjusted life years (QALYs), as measured by EQ-5D-3L [49], and the corresponding costs for each perspective. In addition, the cost-effectiveness analysis will measure the effectiveness in terms of prevalence of MSP.
- The perspective of the national health system will include the direct costs of the Spanish public health services (direct costs of the disease: visits to the general practitioner, specialists, diagnostic tests and medication) and the costs of the intervention; the social perspective, will include all these costs and also the loss of production (indirect costs).
- Direct costs will be calculated from administrative data from clinical records. Indirect costs will be estimated using the approximation of human capital due to disability (company records).
- The costs of the intervention (that is, the time of the experts) will be obtained from the study records and will be converted into unit costs according to the corresponding collective agreements.

- **Statistical analyses**

Special considerations for the cluster analyses will be taken into account. Most inferences will be referred to at the group level, but as recommended [60], adjusted and unadjusted estimates will be obtained for the grouping.

Statistical analyzes adapted for randomized controlled trials with clusters will be carried out. Descriptive analyzes of the characteristics of the participants and of the characteristics referred to the intervention and control groups will be carried out through traditional tests, including t test for continuous and normal variables; Wilconxon test for non-normal variables; McNemar test or chi square for categorical variables. For repeated measurements, the ANOVA, Friedman or Cochran Q tests will be applied according to the typology of the variables. The procedure of generalized estimation equations (GEE) will be used for the MSP analysis, the work functioning and the organizational preventive culture; comparing the baseline difference with the 12-month follow-up of the intervention group, with respect to the baseline moment difference, the 12-month follow-up of the control group. The models will be adjusted by the cluster design and by possible confounding variables.

The incidence of sickness absence will be analyzed through a logistic regression model. A Cox proportional hazard model will estimate the risk ratio of returning to work rather after intervention in the intervention group compared to the control group.

The statistical analyzes will be carried out with STATA 13 (StataCorp, 2013). Stata Statistical Software: version 13. College Station, TX: StataCorp LP).

## ETHICAL REQUIREMENTS

- **Data confidentiality**

All the data collected during this study will be analyzed exclusively by the research team of the INTEVAL_Spain Project. The information will be used anonymously and will always be identified numerically, avoiding revealing any type of private information. The treatment of the collected data will be carried out in an aggregate manner, never individually. The company will not have access to the information collected, and it will never be used for any purpose other than the INTEVAL_Spain Project. Both the members of the Commission and the professionals who participate giving administrative support to case management must guarantee the confidentiality of the data of the professionals included in the project.

The Center for Research in Occupational Health (CISAL), Pompeu Fabra University (UPF), Barcelona, undertakes to treat all information with strict confidentiality, complying with the

provisions of Organic Law 15/1999, on data protection, and other current legislation on data protection.

- **Voluntariness**

The collaboration in the INTEVAL_Spain Project is totally voluntary. In case of not wanting to collaborate in the project, this will not harm the worker in any work aspect. In addition, following the regulations on data protection (Organic Law 15/1999 on the Protection of Personal Data), if you wish to cancel your data in relation to this program, you can contact the medical team of the Prevention service.

- **Approval of the clinical research ethics committee**

Prior to the implementation of the program, this project (nº2014 / 5714 / I) obtained the approval from the Clinical Research Ethics Committee of the Parc de Salut Mar (Annex 6).

## STRENGTHS AND LIMITATIONS

The randomized controlled trial (RCT) is the basic methodological paradigm for the evaluation of health interventions. Randomization ensures that the allocation of a unit of work in the intervention or control group is exclusively due to chance, thus avoiding effects of confusion and selection biases. The availability of a control group makes it possible to distinguish between epidemiological associations and/or statistics and cause-effect relationships, which is fundamental in etiological studies. Also, the prospective design with at least one measure before the intervention and several measurements afterwards allows to study the changes over time.

However, the implementation of controlled trials in the workplace is much more complicated and difficult than in the clinical setting. This is one of the great challenges in this type of intervention studies in occupational health, for which the following aspects have been required to be more adequate [61]: (1) quality of the intervention; (2) quality of the intervention process; (3) sample selection and size; (4) theoretical basis; (5) random assignment; (6) follow-up time; (7) statistical analyzes.

All these elements have been carefully considered in the design of the present study. However, alterations in the implementation process, participation problems and the contamination of the control groups are potential threats.

## BIBLIOGRAPHY


1. Almodóvar A, Galiana ML, Hervás P, et al. VII Encuesta Nacional de Condiciones de Trabajo. Madrid: Instituto Nacional de Seguridad e Higiene en el Trabajo; 2011.
2. Holtermann A, Clausen T, Aust B, Mortensen OS, Andersen LL. Risk for low back pain from diferent frequencies, load mass and trunk postures of lifting and carrying among female healthcare workers. Int Arch Occup Environ Health. 2013;86–4:463–470.
3. National Research Council. Steering Committee for the Workshop on Work-Related Musculoskeletal Injuries. Work-Related Musculoskeletal Disorders: Report, Workshop Summary, and Workshop Papers. Washington: National Academy Press; 1999.
4. Engel G. The need for a new medical model: A challenge for biomedicine. Science 1977;196:129-36.
5. Pincus T, Kent P, Bronfort G, Loisel P, Pransky G, Hartvigsen J. Twenty-five years with the biopsychosocial model of low back pain - is it time to celebrate? A report from the twelfth international forum for primary care research on low back pain. Spine. 2013;38: 2118–23.
6. Marras WS. State-of-the-art research perspectives on musculoskeletal disorder causation and control: the need for an intergraded understanding of risk. 2004. J Elect Kines. 2004;14:1–5.
7. Malchaire J, Cock N, Vergrachat S. Review of the factors associated with musculoskeletal problems in epidemiological studies. Int Arch Occup Environ Health. 2001; 74: 79-90.
8. Luttman A, Jager M, Griefahn B, Caffier G, Liebers F, Steinberg U. Preventing musculoskeletal disorders in the workplace. Geneve: World Health Organization; 2003.
9. Devereux J, Rydstedt L, Nelly V, Weston P, Buckle P. The role of work stress and psychological factors in the development of musculoskeletal disorders. Norwich: Health and Safety Executive Books; 2004.
10. Hauke A, Flintrop J, Brun E, Rugulies R. The impact of work-related psychosocial stressors on the onset of musculoskeletal disorders in specific body regions: a review and meta-analysis of 54 longitudinal studies. Work & Stress. 2011; 25(3): 243-256
11. Zohar D. Safety climate in industrial organizations: theoretical and applied implications. J Appl Psicol. 1980;65(1);96–102.
12. Glendon AI, Stanton NA. Perspectives on safety culture. Safety Sci. 2000; 34:193–214.
13. Garcia AM, Boix P, Canosa C. Why do workers behave unsafely at work? Determinants of safe work practices in industrial workers. Occup Environ Med. 2004; 61:239–46.

14. Gershon RR, Stone PW, Zeltser M, Faucett J, MacDavitt K, Chou SS. Organizational climate and nurse health outcomes in the United States: a systematic review. Ind Health. 2007;45(5):622-36.
15. Gimeno D, Benavides FG, Benach J, Amick BC 3rd. Distribution of sickness absence in the European Union countries. Occup Environ Med. 2004;61(10):867-9.
16. Benavides FG. Ill health, social protection, labour relations, and sickness absence. Occup Environ Med. 2006; 63(4): 228–229.
17. Aaviksoo E, Kiivet RA. Sickness benefit cuts mainly affect blue-collar workers. Scand J Public Health. 2014;42(6):497-503.Driessen et al, 2010)
18. Chou R, Shekelle P. Will this patient develop persistent disabling low back pain? JAMA. 2010;303(13):1295-302.
19. Coggon D, Ntani G, Palmer KT, Felli VE, Harari R, Barrero LH, et al. Disabling musculoskeletal pain in working populations: is it the job, the person, or the culture?. Pain. 2013;154(6):856-63.
20. Vargas-Prada S, Martínez JM, Coggon D, Delclos G, Benavides FG, Serra C. Health beliefs, low mood, and somatizing tendency: contribution to incidence and persistence of musculoskeletal pain with and without reported disability. Scand J Work Environ Health. 2013;39:589–598.
21. Rasmussen CDN, Holtermann A, Mortensen OS, Søgaard K, Jørgensen MB. Prevention of low back pain and its consequences among nurses' aides in elderly care: a stepped-wedge multi-faceted cluster-randomized controlled trial. BMC Public Health. 2013;13:1088.
22. World Health Organization. Towards a Common Language for Functioning, Disability and Health - ICF. Geneva: WHO; 2002.
23. Smedley J, Harris EC, Cox V, Ntani G, Coggon D. Evaluation of a case management service to reduce sickness absence. Occup Med. 2013;63(2):89-95.
24. Demou E, Gibson I, Macdonald EB. Identification of the factors associated with outcomes in a Condition Management Programme. BMC Public Health. 2012;12:927.
25. Hoefsmit N, Houkes I, Nijhuis FJ. Intervention characteristics that facilitate return to work after sickness absence: a systematic literature review. J Occup Rehabil. 2012;22(4):462-77.
26. Westgaard RH, Winkel J. Ergonomic intervention research for improved musculoskeletal health: a critical review. Int J Ind Ergon. 1997; 20: 463–500.

27. Silverstein B, Clark R. Interventions to reduce work-related musculoskeletal disorders. J Elect Kines. 2004;14: 135–152.
28. Cole D, Rivilis I, Van Eerd D, Cullen K, Irvin E, Kramer D. Effectiveness of Participatory Ergonomic Interventions: A Systematic Review. Toronto, Ontario: Institute for Work & Health; 2005.
29. Tompa E, Dolinschi R, Natale J. Economic evaluation of a participatory ergonomics intervention in a textile plant. Appl Ergonomics. 2013; 44: 480-7.
30. Lallemand C. Contributions of participatory ergonomics to the improvement of safety culture in an industrial context. Work. 2012;41 Suppl 1:3284-90.
31. Brown JD, Mackay E, Demou J, Craig J, Frank J, Macdonald E. The EASY (Early Access to Support for You) sickness absence service: A four year evaluation of the impact on absenteeism. Scand J Work Environ Health. 2015; 41(2):204-215.
32. Case Management Society UK (CMSUK) [Internet]. Case management [accessed 23rd March 2015]. Available from: http://www.cmsuk.org/content.aspx?content=4.
33. Kamper SJ, Apeldoorn AT, Chiarotto A, Smeets RJ, Ostelo RW, Guzman J, et al. Multidisciplinary biopsychosocial rehabilitation for chronic low back pain. Cochrane Database Syst Rev. 2014;9:CD000963.
34. The National Institute of Occupational Safety and Health (NIOSH). NIOSH Total Worker Health. Webinar Series. https://www.cdc.gov/niosh/twh/. Accessed 21 Mar 2014.
35. 29. Zeller JM, Levin PF. Mindfulness interventions to reduce stress among nursing personnel: An occupational health perspective. Workplace Health Saf. 2013;61–2:85–89.
36. 30. Tschentscher M, Niederseer D, Niebauer J. Health benefits of Nordic Walking: a systematic review. Am J Prev Med. 2013;44–1:76–84.
37. 31. Martínez-González MA, Corella D, Salas-Salvadó J, Ros E, Covas MI, Fiol M et al. Cohort Profile: design and methods of the PREDIMED study. Int J Epidemiol. 2012;41–2:377–85.
38. 32. Saulle R, Semyonov L, La Torre G. Cost and cost-effectiveness of the Mediterranean diet: results of a systematic review. Nutrients. 2013;5–11:4566–4586.
39. Kramer DM, Wells RP, Carlan N, Aversa T, Bigelow PP, Dixon SM, et al. Did you have an impact? A theory-based method for planning and evaluating knowledge-transfer and exchange activities in occupational health and safety. Int J Occup Saf Ergon. 2013;19(1):41-62

40. Best ML. An ecology of text: using text retrieval to study a life on the net. Artif Life. 1997;3–4:261–287.
41. Evanoff BA, Bohr PC, Wolf LD. Effects of a participatory ergonomics team among hospital orderlies. Am J Ind Med. 1999;35–4:358–365.
42. Gadea R, Sevilla M, García A. ERGOPAR 2.0. Un procedimiento de ergonomía participativa para la prevención de trastornos musculoesqueléticos de origen laboral. Madrid: Instituto Sindical de Trabajo, Ambiente y Salud (ISTAS); 2014.
43. García AM, Gadea R, Sevilla MJ, Ronda E. Validación de un cuestionario para identificar daños y exposición a riesgos ergonómicos en el trabajo. Rev Esp Salud Pública. 2011;85:331–340.
44. Haines H, Wilson JR. Development of a framework for participatory ergonomics. Norwich: Health and Safety Executive Books; 1998.
45. Hill JC, Dunn KM, Lewis M, Mullis R, Main CJ, Foster NE, Hay EM. A Primary Care Back Pain Screening Tool: Identifying Patient Subgroups for Initial Treatment. Arthritis Rheum. 2008;59–5:632–641.
46. Hill JC, Whitehurst DGT, Lewis M, Bryan S, Dunn KM, Foster NE, et al. Comparison of stratified primary care management for low Back pain with current best practice (STarT Back): a randomized controlled trial. Lancet. 2011;378:1560–1571.
47. Vargas-Prada S, Martínez JM, Coggon D, Delclos G, Benavides FG, Serra C. Health beliefs, low mood, and somatizing tendency: contribution to incidence and persistence of musculoskeletal pain with and without reported disability. Scand J Work Environ Health. 2013;39:589–598.
48. Sánchez-López P, Dresch V. The 12-Item General Health Questionnaire (GHQ-12): reliability, external validity and factor structure in the Spanish population. Psicothema. 2008;20–4:839–843.
49. Williams A. EQ-5D concepts and methods. Eds. Kind P, Brooks R, Rabin R. Dordrecht: Springer. 2005:1–17.
50. Schwarzer R, Jerusalem M. Generalized Self-Efficacy scale. In: Weinman J, Wright S, Johnston M, editors. Measures in health psychology: A user's portfolio. Causal and control beliefs.Windsor: NFER-NELSON; 1995. pp. 35–37.
51. Bayliss EA, Ellis JL, Steiner JF. Seniors' self-reported multimorbidity captured biopsychosocial factors not incorporated into two other data-based morbidity measures. J Clin Epidemiol. 2009;62–5:550–557.

52. Bishop SR. What do we really know about mindfulness-based stress reduction? Psychosom Med. 2002;64:71–84.
53. Ramada JM, Serra C, Amick BC, III, Castano JR, Delclos GL. Cross-Cultural Adaptation of the Work Role Functioning Questionnaire to Spanish Spoken in Spain. J Occup Rehabil. 2013;23–4:566–575.
54. Ramada JM, Serra C, Amick BC, III, Abma FI, Castano JR, Pidemunt G, et al. Reliability and Validity of the Work Role Functioning Questionnaire (Spanish Version) J Occup Rehabil. 2014;24–4:640–649.
55. Ramada JM, Delclos GL, Amick BC, III, Abma FI, Pidemunt G, Castano JR, et al. Responsiveness of the Work Role Functioning Questionnaire (Spanish Version) in a General Working Population. J Occup Environ Med. 2014;56–2:189–194.
56. Amick BC, III, Lerner D, Rogers WH, Rooney T, Katz JN. A review of health-related work outcome measures and their uses, and recommended measures. Spine. 2000;25:3152–3160.
57. Organizational Indices Committee of the Occupational Health and Safety Council of Ontario . Benchmarking organizational leading indicators for the prevention and management of injuries and illnesses. Toronto: Institute for Work & Health; 2011.
58. Linnan L, Steckler A. Process evaluation for public health interventions and research. An overview. San Francisco: Jossey-bass; 2002.
59. Wierenga D, Engbers LH, van Empelen P, Hildebrandt VH. The design of a real-time formative evaluation of the implementation process of lifestyle interventions at two worksites using a 7-step strategy (BRAVO@work) BMC Public Health. 2012;12:619.
60. Campbell MK, Piaggio G, Elbourne DR, Altman DG. Consort 2010 statement: extension to cluster randomised trials. BMJ. 2012;345:e5661.
61. Kirstensen TS, Hannerz H, Hogh A, Borg V. The Copenhagen Psychosocial Questionnaire— a tool for the assessment and improvement of the psychosocial work environment. Scand J Work Environ Health 2005;31(6):438-449

# ANNEXES

## ANEXO 1: FICHA INFORMATIVA DEL PROYECTO INTEVAL_SPAIN Y CONSENTIMIENTO INFORMADO

### FICHA INFORMATIVA DEL PROYECTO INTEVAL

El Centro de Investigación en Salud Laboral (CISAL), de la Universidad Pompeu Fabra (UPF), de Barcelona, está desarrollando el Proyecto INTEVAL en el Parc de Salut Mar (PSMAR), Barcelona.

**OBJETIVO:** El Proyecto INTEVAL consiste en una intervención multifactorial, compuesta de tres partes coordinadas entre sí:

1. Ergonomía Participativa, basada en el Método ERGOPAR, cuyo objetivo es identificar y mejorar las condiciones y situaciones de trabajo asociadas a la aparición de trastornos musculoesqueléticos (TME) en los trabajadores, y que incorpora la participación de los trabajadores a lo largo de dicho proceso.
2. Gestión de Casos, que consiste en el diagnóstico precoz y manejo personalizado de aquellos trabajadores afectados por TME discapacitantes, así como la posible adecuación y facilitación del retorno al trabajo, en condiciones seguras, de aquellos trabajadores en situación de Incapacidad Temporal (IT) a causa de un TME discapacitante.
3. Promoción de la salud que consiste en el ofrecimiento de varias actividades grupales para fomentar el ejercicio físico, la dieta mediterránea, el bienestar emocional

**PARTICIPACIÓN:**

Su participación en este proyecto es sumamente importante, ya que contribuirá a proporcionarnos datos muy valiosos, a partir de los cuales se podrán planificar y desarrollar cambios destinados a mejorar las condiciones actuales de trabajo de los trabajadores.

Durante el año de transcurso del estudio todos los trabajadores deberán cumplimentar varios cuestionarios sobre la percepción de su propia salud y sobre las exigencias y condiciones de su actual puesto de trabajo, capacidad funcional, calidad de vida y creencias de salud; respondiendo a preguntas sobre datos personales, datos sobre su trabajo actual, molestias físicas durante el tiempo de trabajo, si le impidieron o no realizar las acciones propias de su puesto laboral, su opinión sobre las causas y su opinión sobre su salud en general.

Durante la Ergonomía Participativa, todos los trabajadores tendrán oportunidad de participar en el proceso de identificación de problemas y soluciones liderados por los referentes de cada unidad. Éstos participarán a su vez en las reuniones de trabajo planificadas.

Durante la Gestión de Casos, podrá ser contactado telefónicamente para llevar un seguimiento de la evolución de su estado de salud, pudiendo ser derivado a alguno de los servicios previstos.

Durante las actividades de Promoción de la salud, los trabajadores que se inscriban adquirirán conocimientos sobre cómo tener cuidado de su propia salud (Marcha Nórdica, Mindfulness y alimentación saludable) y se comprometerán a asistir a todas las sesiones de las actividades a las que se haya inscrito.

**CONFIDENCIALIDAD:** Todos los datos recopilados durante este estudio serán analizados, exclusivamente por el equipo investigador del Proyecto INTEVAL. La información será utilizada de forma anónima y será identificada siempre de forma numérica, evitando revelar cualquier tipo de información privada. El tratamiento de los datos recopilados se llevará a cabo de manera agregada, nunca de manera individual. La empresa no tendrá acceso a la información recopilada, y ésta nunca será utilizada para otro fin que no sea el Proyecto INTEVAL.

El Centro de Investigación en Salud Laboral (CISAL), de la Universidad Pompeu Fabra (UPF), de Barcelona, se compromete a tratar toda la información con estricta confidencialidad, cumpliendo lo previsto en la Ley Orgánica 15/1999, de protección de datos, y demás legislación vigente en materia de protección de datos.

**VOLUNTARIEDAD:** Su colaboración en el Proyecto INTEVAL es totalmente voluntaria. En caso de no querer colaborar en el proyecto, esto no le perjudicará en ningún aspecto laboral. También puede, si cambia de opinión, rescindir su colaboración en el proyecto en cualquier momento, informando de su decisión al equipo de investigación a la mayor brevedad posible.

Si tiene alguna duda durante el desarrollo del proyecto, puede ponerse en contacto con Jose Mª Ramada, responsable del Proyecto INTEVAL a través del siguiente E-mail: proyectotinteval@gmail.com

Muchas gracias por su tiempo y su colaboración.

Firma responsable Proyecto INTEVAL:

## CONSENTIMIENTO INFORMADO

**TÍTULO DEL ESTUDIO:**

Proyecto INTEVAL. Evaluación de una intervención multifactorial en el lugar de trabajo para la prevención y gestión de trastornos musculoesqueléticos en los trabajadores de empresas públicas.

**DECLARACIÓN DE PARTICIPACIÓN VOLUNTARIA:**

Declaro que he sido informado/a de la naturaleza y el propósito del Proyecto INTEVAL, de los datos que se me pide que proporcione y de las acciones que llevaré a cabo mediante mi participación. He recibido una explicación satisfactoria sobre los procedimientos del proyecto y su finalidad.

Comprendo que mi decisión de participar es voluntaria.

Presto mi consentimiento para la recopilación de datos y para la cumplimentación de diversos cuestionarios, así como para la participación en las acciones que se realizarán durante el desarrollo del proyecto.

Conozco mi derecho a retirar este consentimiento cuando lo desee, con la única obligación de informar mi decisión, a la mayor brevedad posible, al Equipo Investigador INTEVAL.

☐ Sí ACEPTO PARTICIPAR voluntariamente en este estudio, firme a continuación e indíquenos su nombre y apellidos, así como la fecha actual.

☐ NO ACEPTO PARTICIPAR voluntariamente en este estudio, firme a continuación e indíquenos su nombre y apellidos, así como la fecha actual.

| | |
|---|---|
| Firma trabajador/a: | Firma de la persona que obtiene el consentimiento: |
| Nombre trabajador/a: | Dra.Consol Serra Pujadas |
| Fecha: | |

## ANEXO 2: CUESTIONARIO BASAL

**CUESTIONARIO ERGOPAR**

Apreciado/a compañero/a:

Tu participación completando este cuestionario es totalmente anónima y voluntaria, y nos será muy útil para identificar necesidades de mejora en este tema.

Es muy importante que respondas según tu propia opinión y con total sinceridad, pues sólo así podremos extraer conclusiones válidas. Por favor, completa toda la información que se solicita o señala con una X la casilla que mejor represente tu opinión al respecto.

Todo el tratamiento de los datos obtenidos lo realizará, de manera confidencial, el equipo investigador. De esta manera se garantiza por completo el anonimato de los trabajadores encuestados, y en ningún momento se relacionarán las respuestas con personas identificables.

Si tienes cualquier duda o comentario sobre este cuestionario, puedes contactar con Anna Amat.

Muchas gracias por tu participación,

Anna Amat

UPF – CiSAL

**DATOS PERSONALES Y LABORALES**

**1.** Eres: ☐ Hombre Mu[illegible] ☐

**2.** Edad: …… (años)

**3.** ¿Cuánto tiempo llevas trabajando en el PSMAR? ……… (años)
Si es menos de 1 año, marca esta casilla…… ☐

**4.** Antigüedad en el puesto de trabajo actual: ……… (años)
Si es menos de 1 año, marca esta casilla…… ☐

**5.** Tu horario es:

| | | | |
|---|---|---|---|
| Turno de mañana……………………….. | ☐ | Turno de tarde ………………………….. | ☐ |
| Turno de noche………………………….. | ☐ | Otro (especificar)……………………….. | ☐ |

**6.** Habitualmente, ¿cuántas **horas al día** trabajas en este puesto?......... **horas/día**

**7.** Tu contrato es:

| | | | |
|---|---|---|---|
| Indefinido………………………………… | ☐ | Eventual (temporal)………………….. | ☐ |
| D.I. Residente………………………….. | ☐ | Interino/a…………………………………. | ☐ |
| Funcionario……………………………… | ☐ | Otro (especificar)……………………….. | ☐ |

**8.** Indica la unidad en la que actualmente estás trabajando:

| | | | |
|---|---|---|---|
| UGA (HOSPITAL DE LA ESPERANZA)……… | ☐ | UH30 (HOSPITAL DEL MAR)……………… | ☐ |
| LLEVANT 3 o 4 (CAEM)………………….. | ☐ | UCI (HOSPITAL DEL MAR)………………… | ☐ |
| UH04 (HOSPITAL DEL MAR)……………… | ☐ | BLOQUE QUIRÚRGICO (HOSPITAL DEL MAR) | ☐ |

**9.** Trabajas como:

| | | | |
|---|---|---|---|
| Enfermero/a ………………………….. | ☐ | Auxiliar de enfermería…………….. | ☐ |
| Enfermero/a equipo complemento............................. | ☐ | Auxiliar de enfermería equipo complemento………………………….. | ☐ |

**10.** Eres … (puedes señalar más de una opción, si es tu caso):

| | | | |
|---|---|---|---|
| Trabajador/a ………………………….. | ☐ | Responsable de Servicio/UH …… | ☐ |
| Delegado/a de prevención ……… | ☐ | Miembro del Comité de Seguridad y Salud ………………… | ☐ |

## DAÑOS A LA SALUD DERIVADOS DEL TRABAJO

**11.** Para cada zona corporal indica si tienes MOLESTIA O DOLOR, su FRECUENCIA, si te ha IMPEDIDO REALIZAR TU TRABAJO ACTUAL y si esa molestia o dolor se han producido COMO CONSECUENCIA DE LAS TAREAS QUE REALIZAS EN TU ACTUAL PUESTO DE TRABAJO (AL QUE TE HAS REFERIDO EN LA PREGUNTA 9).

| | **¿Tienes molestia o dolor en esta zona?** | | | **¿Con qué frecuencia?** | | | **¿Te ha impedido alguna vez realizar tu TRABAJO ACTUAL?** | | **¿Se ha producido como consecuencia de tu actual PUESTO DE TRABAJO?** | |
|---|---|---|---|---|---|---|---|---|---|---|
| | No | Molestia | Dolor | No | A veces | Muchas veces | No | Sí | No | Sí |
| 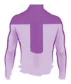 Cuello, hombro y/o espalda dorsal | ☐ | ☐ | ☐ | ☐ | ☐ | ☐ | ☐ | ☐ | ☐ | ☐ |
| 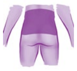 Espalda lumbar | ☐ | ☐ | ☐ | ☐ | ☐ | ☐ | ☐ | ☐ | ☐ | ☐ |
| 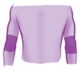 Codos | ☐ | ☐ | ☐ | ☐ | ☐ | ☐ | ☐ | ☐ | ☐ | ☐ |
| 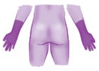 Manos y/o muñecas | ☐ | ☐ | ☐ | ☐ | ☐ | ☐ | ☐ | ☐ | ☐ | ☐ |
| 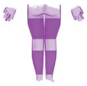 Piernas | ☐ | ☐ | ☐ | ☐ | ☐ | ☐ | ☐ | ☐ | ☐ | ☐ |
| 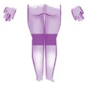 Rodillas | ☐ | ☐ | ☐ | ☐ | ☐ | ☐ | ☐ | ☐ | ☐ | ☐ |
| 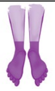 Pies | ☐ | ☐ | ☐ | ☐ | ☐ | ☐ | ☐ | ☐ | ☐ | ☐ |

## POSTURAS PROPIAS DEL TRABAJO

Contesta a cada pregunta SIEMPRE EN RELACIÓN CON UNA JORNADA HABITUAL EN TU ACTUAL PUESTO DE TRABAJO (AL QUE TE HAS REFERIDO EN LA PREGUNTA 9).

**12.** ¿Durante CUÁNTO TIEMPO tienes que trabajar adoptando o realizando estas posturas?

| | **Nunca/Menos de 30 minutos** | **Entre 30 minutos y 2 horas** | **Entre 2 y 4 horas** | **Más de 4 horas** |
|---|---|---|---|---|
| Sentado (silla, taburete, apoyo lumbar, etc.) | ☐ | ☐ | ☐ | ☐ |
| De pie, sin andar apenas | ☐ | ☐ | ☐ | ☐ |
| Caminando | ☐ | ☐ | ☐ | ☐ |
| Caminando mientras subo o bajo niveles diferentes (peldaños, escalera, rampa, etc.) | ☐ | ☐ | ☐ | ☐ |
| De rodillas/en cuclillas | ☐ | ☐ | ☐ | ☐ |

**13.** ¿Durante CUÁNTO TIEMPO tienes que trabajar adoptando o realizando estas posturas forzadas de CUELLO/CABEZA?

| | **Nunca/ Menos de 30 minutos** | **Entre 30 minutos y 2 horas** | **Entre 2 y 4 horas** | **Más de 4 horas** | **Esta postura, ¿tienes que REPETIRLA cada pocos segundos, o MANTENERLA FIJA un tiempo?** | |
|---|---|---|---|---|---|---|
| | | | | | **La repito** | **La mantengo fija** |
| 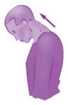 Inclinar el cuello / cabeza hacia delante | ☐ | ☐ | ☐ | ☐ | ☐ | ☐ |
| 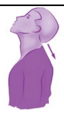 Inclinar el cuello /cabeza hacia atrás | ☐ | ☐ | ☐ | ☐ | ☐ | ☐ |
| 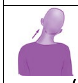 Inclinar el cuello /cabeza hacia un lado o ambos | ☐ | ☐ | ☐ | ☐ | ☐ | ☐ |
| 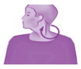 Girar el cuello / cabeza | ☐ | ☐ | ☐ | ☐ | ☐ | ☐ |

**14.** ¿Durante CUÁNTO TIEMPO tienes que trabajar adoptando o realizando estas posturas forzadas de ESPALDA/TRONCO?

| | Nunca/ Menos de 30 minutos | Entre 30 minutos y 2 horas | Entre 2 y 4 horas | Más de 4 horas | Esta postura, ¿tienes que REPETIRLA cada pocos segundos, o MANTENERLA FIJA un tiempo? | |
|---|---|---|---|---|---|---|
| | | | | | La repito | La mantengo fija |
| 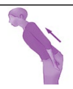 Inclinar la espalda/tronco hacia delante | ☐ | ☐ | ☐ | ☐ | ☐ | ☐ |
| 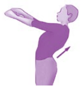 Inclinar la espalda/ tronco hacia atrás | ☐ | ☐ | ☐ | ☐ | ☐ | ☐ |
| 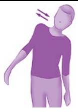 Inclinar la espalda/ tronco hacia un lado o ambos | ☐ | ☐ | ☐ | ☐ | ☐ | ☐ |
| 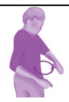 Girar la espalda/ tronco | ☐ | ☐ | ☐ | ☐ | ☐ | ☐ |

**15.** ¿Durante CUÁNTO TIEMPO tienes que trabajar adoptando o realizando estas posturas de HOMBROS, MUÑECAS Y TOBILLOS/PIES?

| | Nunca/ Menos de 30 minutos | Entre 30 minutos y 2 horas | Entre 2 y 4 horas | Más de 4 horas | Esta postura, ¿tienes que REPETIRLA cada pocos segundos, o MANTENERLA FIJA un tiempo? | |
|---|---|---|---|---|---|---|
| | | | | | La repito | La mantengo fija |
| 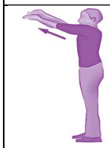 Las manos por encima de la cabeza o los codos por encima de los hombros | ☐ | ☐ | ☐ | ☐ | ☐ | ☐ |
| 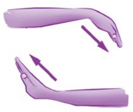 Una o ambas muñecas dobladas hacia arriba o hacia abajo, hacia los lados o giradas (giro de antebrazo) | ☐ | ☐ | ☐ | ☐ | ☐ | ☐ |
| 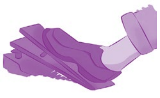 Ejerciendo presión con uno de los pies | ☐ | ☐ | ☐ | ☐ | ☐ | ☐ |

**16.** ¿Durante CUÁNTO TIEMPO tienes que trabajar realizando estas acciones con las MANOS?

| | **Nunca/Menos de 30 minutos** | **Entre 30 minutos y 2 horas** | **Entre 2 y 4 horas** | **Más de 4 horas** |
|---|---|---|---|---|
| 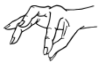 Sostener, presionar o levantar objetos o herramientas con los dedos en forma de pinza | ☐ ☐ | ☐ ☐ | ☐ ☐ | ☐ ☐ |
| 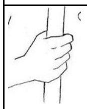 Empujar , agarrar o arrastrar con fuerza objetos o herramientas con las manos | | | | |
| 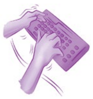 Utilizar de manera intensiva a los dedos (ordenador, botones, etc.) | ☐ | ☐ | ☐ | ☐ |

**17.** MOVILIZACIÓN Y MANIPULACIÓN MANUAL DE PACIENTES. Responde en relación a cada una de las siguientes acciones.

| **MOVILIZACIÓN MANUAL DE PACIENTES** | |
|---|---|
| 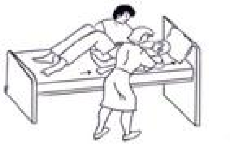 | **¿Durante CUÁNTO TIEMPO tienes que trabajar realizando esta acción DURANTE UNA JORNADA HABITUAL en tu puesto de trabajo actual?** |
| Nunca / Menos de 30 minutos...................... ☐<br>Entre 30 minutos y 2 horas........................... ☐ | Entre 2 y 4 horas............................................. ☐<br>Más de 4 horas................................................ ☐ |
| **La tipología de PACIENTES que manipulas DURANTE UNA JORNADA HABITUAL en tu puesto de trabajo actual es:** | |
| **Paciente colaborador** (puede valerse por sí solo): | |
| Nunca............................................................. ☐<br>Entre 1 y 5 pacientes..................................... ☐ | Entre 6 y 10 pacientes.................................... ☐<br>Más de 10 pacientes....................................... ☐ |
| **Paciente parcialmente colaborador** (debe ser parcialmente levantado): | |
| Nunca............................................................. ☐<br>Entre 1 y 5 pacientes..................................... ☐ | Entre 6 y 10 pacientes.................................... ☐<br>Más de 10 pacientes....................................... ☐ |
| **Paciente no colaborador** (debe ser completamente levantado, no se vale por sí solo): | |
| Nunca............................................................. ☐<br>Entre 1 y 5 pacientes..................................... ☐ | Entre 6 y 10 pacientes.................................... ☐<br>Más de 10 pacientes....................................... ☐ |

| Señala con qué **FRECUENCIA** movilizas pacientes de **PESO ELEVADO:** | | | | |
|---|---|---|---|---|
| | Todos los días | Varias veces por semana | Ocasionalmente | Nunca |
| Pacientes entre 80-150 kg. | ☐ | ☐ | ☐ | ☐ |
| Pacientes de más de 150 kg. | ☐ | ☐ | ☐ | ☐ |

| **Señala con qué FRECUENCIA movilizas PACIENTES:** | | | | | |
|---|---|---|---|---|---|
| | | Todos los días | Varias veces por semana | Ocasionalmente | Nunca |
| 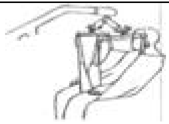 | Con la ayuda de grúas | ☐ | ☐ | ☐ | ☐ |
| 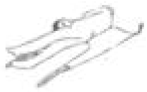 | Con otros equipos de ayuda (transfers, discos, …) | ☐ | ☐ | ☐ | ☐ |
| 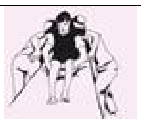 | Con ayuda de otra persona | ☐ | ☐ | ☐ | ☐ |

**MOVILIZACIÓN MANUAL DE PACIENTES (EMPUJAR, ARRASTRAR, DESPLAZAR) SITUADOS EN ALGÚN EQUIPO DE DESPLAZAMIENTO (CAMA, CAMILLA, ETC…)**

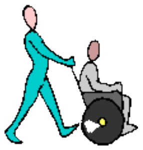
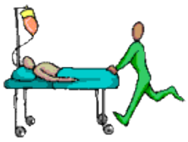

**¿Durante CUÁNTO TIEMPO tienes que trabajar realizando esta acción (empujar o arrastrar pacientes con ayuda de equipos de desplazamiento) DURANTE UNA JORNADA HABITUAL en tu puesto de trabajo actual?**

| | | | |
|---|---|---|---|
| Nunca / Menos de 30 minutos………………………… | ☐ | Entre 2 y 4 horas……………………………………………….. | ☐ |
| Entre 30 minutos y 2 horas……………………………… | ☐ | Más de 4 horas………………………………………………… | ☐ |

| **Señala con qué FRECUENCIA movilizas pacientes SITUADOS EN ALGÚN EQUIPO DE DESPLAZAMIENTO:** | | | | |
|---|---|---|---|---|
| | Todos los días | Varias veces por semana | Ocasionalmente | Nunca |
| Con ayuda de otra persona | ☐ | ☐ | ☐ | ☐ |

**18.** MANIPULACIÓN MANUAL DE CARGAS DE MÁS DE 3 KG EN TOTAL (excluyendo manipulación de pacientes). Responde en relación a cada una de las siguientes tres acciones.

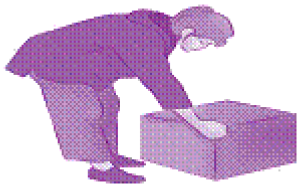

**COGER Y/O DEJAR MANUALMENTE objetos, herramientas o materiales de MÁS DE 3 KG.**

**¿Durante CUÁNTO TIEMPO tienes que trabajar realizando esta acción?**

| | | | |
|---|---|---|---|
| Nunca / Menos de 30 minutos………………………… | ☐ | Entre 2 y 4 horas……………………………………………….. | ☐ |
| Entre 30 minutos y 2 horas……………………………… | ☐ | Más de 4 horas………………………………………………… | ☐ |

**RESPONDE SÓLO SI MANIPULAS CARGAS DE 3 O MÁS KILOS:**

**Los PESOS que con mayor frecuencia coges y/o dejas son de:**

| | | | |
|---|---|---|---|
| Entre 3 y 5 kilos…………………………………………………. | ☐ | Entre 15 y 25 kilos…………………………………………….. | ☐ |
| Entre 5 y 15 kilos……………………………………………….. | ☐ | Más de 25 kilos………………………………………………….. | ☐ |
| **Señala si habitualmente:** | | | |
| ☐ Coges y/o dejas carga tú solo/a (sin ayuda de otra persona) | | | |
| ☐ Coges y/o dejas la carga por debajo de tus rodillas | | | |
| ☐ Coges y/o dejas la carga por encima de tus hombros | | | |
| ☐ Mantienes los brazos extendidos sin poder apoyar la carga en tu cuerpo | | | |
| ☐ Manipulas la carga con dificultad por no tener buen agarre (sin asa) | | | |
| ☐ Tienes que coger y/o dejar la carga cada pocos segundos | | | |

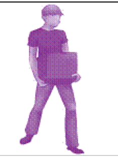

**TRANSPORTAR MANUALMENTE objetos, herramientas o materiales de MÁS DE 3 KG.**

| | | | |
|---|---|---|---|
| **¿Durante CUÁNTO TIEMPO tienes que trabajar realizando esta acción?** | | | |
| Nunca / Menos de 30 minutos……………………….. | ☐ | Entre 2 y 4 horas………………………………………………. | ☐ |
| Entre 30 minutos y 2 horas……………………………. | ☐ | Más de 4 horas…………………………………………………. | ☐ |
| **RESPONDE SÓLO SI MANIPULAS CARGAS DE 3 O MÁS KILOS:** | | | |
| **Los PESOS que con mayor frecuencia coges y/o dejas son de:** | | | |
| Entre 3 y 5 kilos…………………………………………………. | ☐ | Entre 15 y 25 kilos…………………………………………….. | ☐ |
| Entre 5 y 15 kilos……………………………………………….. | ☐ | Más de 25 kilos………………………………………………….. | ☐ |
| **Señala si habitualmente:** | | | |
| ☐ Transportas la carga tú solo/a (sin la ayuda de otra persona) | | | |
| ☐ Transportas la carga con los brazos extendidos, sin apoyar la carga en tu cuerpo y sin doblar los codos | | | |
| ☐ Transportas la carga con dificultad, por no tener un buen agarre | | | |
| ☐ Caminas más de 10 metros transportando la carga | | | |
| ☐ Tienes que transportar la carga cada pocos segundos | | | |

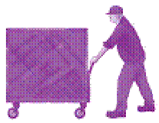

**EMPUJAR Y/O ARRASTRAR MANUALMENTE o utilizando algún equipo (carro de enfermería, de ropa, de medicamentos… ) de MÁS DE 3 KG.**

| | | | |
|---|---|---|---|
| **¿Durante CUÁNTO TIEMPO tienes que trabajar realizando esta acción?** | | | |
| Nunca / Menos de 30 minutos……………………….. | ☐ | Entre 2 y 4 horas………………………………………………. | ☐ |
| Entre 30 minutos y 2 horas……………………………. | ☐ | Más de 4 horas…………………………………………………. | ☐ |
| **Señala si habitualmente:** | | | |
| ☐ Tienes que hacer mucha fuerza para iniciar el empuje y/o arrastre | | | |

| | |
|---|---|
| ▢ | Tienes que hacer mucha fuerza para desplazar la carga |
| ▢ | La zona donde tienes que poner las manos al empujar y/o arrastrar no es adecuada (muy alta, muy baja, difícil de agarrar, etc.) |
| ▢ | Tienes que caminar más de 10 metros empujando y/o arrastrando la carga |
| ▢ | Tienes que empujar y/o arrastrar la carga cada pocos segundos |

**19.** En general, ¿cómo valorarías las **EXIGENCIAS FÍSICAS** EN TU ACTUAL PUESTO DE TRABAJO (AL QUE TE HAS REFERIDO EN LA PREGUNTA 9)?

Muy bajas………………………………………………………………………… ▢
Bajas……………………………………………………………………………… ▢
Moderadas……………………………………………………………………… ▢
Altas……………………………………………………………………………… ▢
Muy altas………………………………………………………………………… ▢

**20.** En relación a las POSTURAS Y ACCIONES PROPIAS EN TU ACTUAL PUESTO DE TRABAJO, **ordena del 1 al 5** las acciones que piensas que **afectan más a tu SALUD Y BIENESTAR** (siendo 1 la acción que afecte más, y 5 la que menos)

Movilizaciones y transferencias…………………………………………... ▢

Bipedestación prolongada…………………………………………………… ▢

Manipulación de cargas……………………………………………………… ▢

Factores psicosociales………………………………………………………… ▢

Espacio/instalaciones/factores ambientales………………………… ▢

Otros (especificar)……………………………………………………………. ▢
……………………………………………………………………………………………
……………………………………………………………………………………………
……………………………………………………………………………………………
……………………………………………………………………………………………

**¿Qué prácticas de seguridad y salud se realizan en el PSMAR?**

**Nos interesa conocer tu grado de acuerdo con cada una de las siguientes afirmaciones, señalando SÍ o NO dependiendo de si estás de acuerdo o no con la afirmación, o rodeando con un círculo la puntuación que mejor represente tu opinión al respecto. El valor "0" significa que estás totalmente en desacuerdo con la afirmación, y el valor "10" significa que estás totalmente de acuerdo. En caso de que haya alguna cuestión que desconozcas, señala la opción "No sé".**

**21.** En mi empresa hay personas cuyo trabajo consiste en ocuparse de la prevención de riesgos laborales:

☐ SÍ ☐ NO ☐ No sé

**22.** Las personas que se encargan de la prevención de riesgos laborales en mi empresa tienen autoridad suficiente para llevar a cabo todas las mejoras y cambios necesarios para proteger la salud y seguridad de los trabajadores:

| Totalmente en desacuerdo | | | | | | | | | | Totalmente de acuerdo | |
|---|---|---|---|---|---|---|---|---|---|---|---|
| 0 | 1 | 2 | 3 | 4 | 5 | 6 | 7 | 8 | 9 | 10 | ☐ No sé |

**23.** En mi empresa se le da tanta importancia a la salud y seguridad de los trabajadores como al rendimiento y calidad en el trabajo:

| Totalmente en desacuerdo | | | | | | | | | | Totalmente de acuerdo | |
|---|---|---|---|---|---|---|---|---|---|---|---|
| 0 | 1 | 2 | 3 | 4 | 5 | 6 | 7 | 8 | 9 | 10 | ☐ No sé |

**24.** Valoro muy positivamente las actuaciones y mejoras en materia de seguridad y salud que puedan afectar a mi puesto de trabajo:

| Totalmente en desacuerdo | | | | | | | | | | Totalmente de acuerdo | |
|---|---|---|---|---|---|---|---|---|---|---|---|
| 0 | 1 | 2 | 3 | 4 | 5 | 6 | 7 | 8 | 9 | 10 | ☐ No sé |

**25.** En mi puesto de trabajo se realizan regularmente (al menos una vez cada año) evaluaciones y/o intervenciones para mejorar los aspectos de seguridad y salud en el trabajo:

☐ SÍ ☐ NO ☐ No sé

**26.** Dispongo de toda la información que necesito para trabajar de forma segura y sin perjudicar mi salud:

| Totalmente en desacuerdo | | | | | | | | | | Totalmente de acuerdo | |
|---|---|---|---|---|---|---|---|---|---|---|---|
| 0 | 1 | 2 | 3 | 4 | 5 | 6 | 7 | 8 | 9 | 10 | ☐ No sé |

**27.** En mi empresa se considera la opinión del trabajador respecto a las decisiones que afectan a su seguridad y salud:

| Totalmente en desacuerdo | | | | | | | | | | Totalmente de acuerdo | |
|---|---|---|---|---|---|---|---|---|---|---|---|
| 0 | 1 | 2 | 3 | 4 | 5 | 6 | 7 | 8 | 9 | 10 | ☐ No sé |

**28.** Obtengo un reconocimiento positivo por parte de mis encargados y superiores si realizo mis tareas evitando la exposición a riesgos laborales, protegiendo mi salud y/o la de mis compañeros:

| Totalmente en desacuerdo | | | | | | | | | | Totalmente de acuerdo | |
|---|---|---|---|---|---|---|---|---|---|---|---|
| 0 | 1 | 2 | 3 | 4 | 5 | 6 | 7 | 8 | 9 | 10 | ☐ No sé |

**29.** Dispongo de las herramientas y/o equipos necesarios para trabajar de manera segura y sin perjudicar mi salud:

| Totalmente en desacuerdo | | | | | | | | | | Totalmente de acuerdo | |
|---|---|---|---|---|---|---|---|---|---|---|---|
| 0 | 1 | 2 | 3 | 4 | 5 | 6 | 7 | 8 | 9 | 10 | ☐ No sé |

**Desempeño y satisfacción laboral en el PSMAR**

**Las siguientes preguntas son acerca de la frecuencia con la que experimentaste determinadas situaciones en relación con la ejecución de tu trabajo en el PSMAR durante las últimas 4 semanas (28 días).**

**30.** Rodea con un círculo la puntuación que mejor refleje tu experiencia en ese periodo.

| | **Todo el tiempo** | **La mayor parte del tiempo** | **A veces** | **Casi nunca** | **Nunca** |
|---|---|---|---|---|---|
| **a.** ¿Con qué frecuencia tuviste **molestias o problemas de salud** que limitaron la cantidad o la calidad del trabajo que podías hacer? | 1 | 2 | 3 | 4 | 5 |
| **b.** ¿Con qué frecuencia tu rendimiento en el trabajo fue **más elevado** que el de la mayoría de los trabajadores en tu sección o puesto de trabajo? | 1 | 2 | 3 | 4 | 5 |
| **c.** ¿Con qué frecuencia tu rendimiento en el trabajo fue **más bajo** que el de la mayoría de los trabajadores en tu sección o puesto de trabajo? | 1 | 2 | 3 | 4 | 5 |
| **d.** ¿Con qué frecuencia **no trabajaste** en momentos en los que se suponía que debías estar trabajando? | 1 | 2 | 3 | 4 | 5 |
| **e.** ¿Con qué frecuencia te percataste de que no estabas trabajando tan **cuidadosamente** como deberías? | 1 | 2 | 3 | 4 | 5 |
| **f.** ¿Con qué frecuencia la **calidad** de tu trabajo fue inferior a lo que debería haber sido? | 1 | 2 | 3 | 4 | 5 |

| | | | | | |
|---|---|---|---|---|---|
| **g.** ¿Con qué frecuencia **no te concentraste** lo suficiente en tu trabajo? | 1 | 2 | 3 | 4 | 5 |

**Responde a las siguientes preguntas relacionadas con tu satisfacción en tu puesto de trabajo actual. Rodea con un círculo la puntuación que mejor represente tu opinión al respecto. El valor "0" significa que no estás nada satisfecho y el valor "6" que estás totalmente satisfecho.**

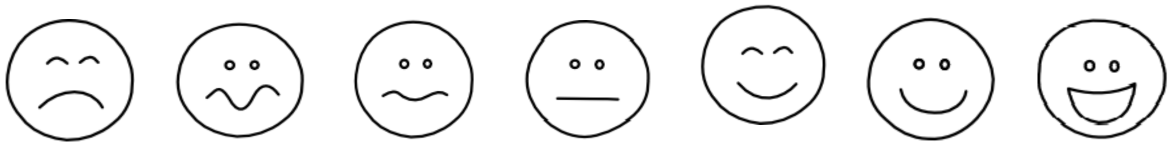

| **Totalmente insatisfecho** | **Muy poco satisfecho** | **Poco satisfecho** | **Neutral** | **Satisfecho** | **Muy satisfecho** | **Totalmente satisfecho** |
|---|---|---|---|---|---|---|
| 0 | 1 | 2 | 3 | 4 | 5 | 6 |

**31.** En relación con tu actual puesto de trabajo en el PSMAR, ¿cómo estás de satisfecha/o con

| | **Totalmente insatisfecho** | **Muy poco satisfecho** | **Poco satisfecho** | **Neutral** | **Satisfecho** | **Muy satisfecho** | **Totalmente satisfecho** |
|---|---|---|---|---|---|---|---|
| **a.** … los compañeros en tu puesto y/o sección de trabajo? | 0 | 1 | 2 | 3 | 4 | 5 | 6 |
| **b.** … las tareas que debes realizar en tu puesto de trabajo? | 0 | 1 | 2 | 3 | 4 | 5 | 6 |
| **c.** … las condiciones de trabajo en tu puesto? | 0 | 1 | 2 | 3 | 4 | 5 | 6 |
| **d.** … tu superior inmediato? | 0 | 1 | 2 | 3 | 4 | 5 | 6 |
| **e.** …tus actuales condiciones de contratación/empleo? | 0 | 1 | 2 | 3 | 4 | 5 | 6 |
| **f.** … los materiales y equipos disponibles para realizar tu trabajo? | 0 | 1 | 2 | 3 | 4 | 5 | 6 |

| | | | | | | | |
|---|---|---|---|---|---|---|---|
| **g.** … tu trabajo en general? | 0 | 1 | 2 | 3 | 4 | 5 | 6 |

**32.** Responde a las siguientes preguntas relacionadas con tu puesto de trabajo actual. Rodea con un círculo la puntuación que mejor represente tu opinión al respecto.

| | **Todo el tiempo** | **La mayor parte del tiempo** | **A veces** | **Casi nunca** | **Nunca** |
|---|---|---|---|---|---|
| **a.** ¿Tienes que trabajar muy rápido? | 1 | 2 | 3 | 4 | 5 |
| **b.** ¿Tienes que trabajar intensamente? (mucha cantidad de trabajo en poco tiempo) | 1 | 2 | 3 | 4 | 5 |
| **c.** ¿Tu trabajo, en general, te exige demasiado? | 1 | 2 | 3 | 4 | 5 |
| **d.** ¿Tu trabajo permite que aprendas cosas nuevas? | 1 | 2 | 3 | 4 | 5 |
| **e.** ¿Tu trabajo exige que muestres iniciativa? | 1 | 2 | 3 | 4 | 5 |
| **f.** ¿Puedes elegir cómo hacer tu trabajo? | 1 | 2 | 3 | 4 | 5 |
| **g.** ¿Puedes elegir qué hacer en tu trabajo? | 1 | 2 | 3 | 4 | 5 |
| **h.** ¿Recibes ayuda y apoyo de tus compañeros de trabajo? | 1 | 2 | 3 | 4 | 5 |
| **i.** ¿Recibes ayuda y apoyo de tu superior inmediato/a? | 1 | 2 | 3 | 4 | 5 |

**TRABAJO Y SALUD**

En las siguientes preguntas le pedimos que nos indique, para las **ÚLTIMAS CUATRO SEMANAS**, la cantidad de tiempo en que tuvo dificultad para realizar ciertos aspectos de su trabajo. Marque la casilla **"NO aplicable a mi trabajo"** sólo en caso de que la pregunta se refiera a algo que no es parte de su trabajo.

**33.** En las ÚLTIMAS CUATRO SEMANAS, ¿durante cuánto tiempo de su trabajo le fue difícil realizar las siguientes actividades por motivos de su salud física o problemas emocionales?

| | **Fue difícil todo el tiempo (100%)** | **Fue difícil la mayor parte del tiempo** | **Fue difícil la mitad del tiempo (50%)** | **Fue difícil una parte del tiempo** | **Nunca fue difícil (0%)** | **NO aplicable a mi trabajo** |
|---|---|---|---|---|---|---|
| **a.** Trabajar el número de horas requeridas | ☐ | ☐ | ☐ | ☐ | ☐ | ☐ |
| **b.** Empezar la jornada de trabajo con facilidad | ☐ | ☐ | ☐ | ☐ | ☐ | ☐ |
| **c.** Ponerse a trabajar nada más llegar al trabajo | ☐ | ☐ | ☐ | ☐ | ☐ | ☐ |
| **d.** Hacer su trabajo sin parar a hacer descansos adicionales | ☐ | ☐ | ☐ | ☐ | ☐ | ☐ |
| **e.** Ajustarse a una rutina u horario | ☐ | ☐ | ☐ | ☐ | ☐ | ☐ |
| **f.** Manejar su carga de trabajo | ☐ | ☐ | ☐ | ☐ | ☐ | ☐ |
| **g.** Trabajar lo suficientemente rápido | ☐ | ☐ | ☐ | ☐ | ☐ | ☐ |
| **h.** Acabar el trabajo a tiempo | ☐ | ☐ | ☐ | ☐ | ☐ | ☐ |

| | | | | | | |
|---|---|---|---|---|---|---|
| **i.** Hacer su trabajo sin cometer errores | ☐ | ☐ | ☐ | ☐ | ☐ | ☐ |
| **j.** Satisfacer a las personas que evalúan su trabajo | ☐ | ☐ | ☐ | ☐ | ☐ | ☐ |
| | **Fue difícil todo el tiempo (100%)** | **Fue difícil la mayor parte del tiempo** | **Fue difícil la mitad del tiempo (50%)** | **Fue difícil una parte del tiempo** | **Nunca fue difícil (0%)** | **NO aplicable a mi trabajo** |
| **k.** Tener sensación de trabajo bien hecho | ☐ | ☐ | ☐ | ☐ | ☐ | ☐ |
| **l.** Sentir que ha hecho lo que es capaz de hacer | ☐ | ☐ | ☐ | ☐ | ☐ | ☐ |
| **m.** Caminar o desplazarse a distintos lugares de trabajo | ☐ | ☐ | ☐ | ☐ | ☐ | ☐ |
| **n.** Levantar, cargar o mover objetos de más de 5 kg de peso, en el trabajo | ☐ | ☐ | ☐ | ☐ | ☐ | ☐ |
| **ñ.** Permanecer sentado, de pie o en una misma posición durante más de 15 minutos, mientras se trabaja | ☐ | ☐ | ☐ | ☐ | ☐ | ☐ |
| **o.** Repetir los mismos movimientos una y otra vez mientras trabaja | ☐ | ☐ | ☐ | ☐ | ☐ | ☐ |
| **p.** Doblarse, girarse o alcanzar un objeto mientras trabaja | ☐ | ☐ | ☐ | ☐ | ☐ | ☐ |
| **q.** Usar equipos o herramientas de mano | ☐ | ☐ | ☐ | ☐ | ☐ | ☐ |
| **r.** Mantener la mente en su trabajo | ☐ | ☐ | ☐ | ☐ | ☐ | ☐ |

| | | | | | | |
|---|---|---|---|---|---|---|
| **s.** Pensar con claridad mientras trabaja | ☐ | ☐ | ☐ | ☐ | ☐ | ☐ |
| **t.** Hacer el trabajo con cuidado | ☐ | ☐ | ☐ | ☐ | ☐ | ☐ |
| **u.** Concentrarse en su trabajo | ☐ | ☐ | ☐ | ☐ | ☐ | ☐ |
| **v.** Trabajar sin perder el hilo (de las ideas) | ☐ | ☐ | ☐ | ☐ | ☐ | ☐ |
| | **Fue difícil todo el tiempo (100%)** | **Fue difícil la mayor parte del tiempo** | **Fue difícil la mitad del tiempo (50%)** | **Fue difícil una parte del tiempo** | **Nunca fue difícil (0%)** | **NO aplicable a mi trabajo** |
| **w.** Leer o usar los ojos con facilidad mientras trabaja | ☐ | ☐ | ☐ | ☐ | ☐ | ☐ |
| **x.** Hablar con la gente cara a cara, en reuniones o por teléfono | ☐ | ☐ | ☐ | ☐ | ☐ | ☐ |
| **y.** Controlar su genio delante de otras personas mientras trabaja | ☐ | ☐ | ☐ | ☐ | ☐ | ☐ |
| **z.** Ayudar a otras personas a acabar el trabajo | ☐ | ☐ | ☐ | ☐ | ☐ | ☐ |

**34.** Seleccione la afirmación en cada sección que describa mejor su estado de salud en el día de hoy:

| **a.** | **Movilidad** | | | | |
|---|---|---|---|---|---|
| No tengo problemas para caminar | ☐ | Tengo algunos problemas para caminar | ☐ | Tengo que estar en la cama | ☐ |

| **b.** | **Cuidado personal** | | | | |
|---|---|---|---|---|---|
| No tengo problemas con el cuidado personal | ☐ | Tengo algunos problemas para lavarme o vestirme solo | ☐ | Soy incapaz de lavarme o vestirme solo | ☐ |

| c. | **Actividades de <u>Todos los Días</u>** (ej, trabajar, estudiar, hacer tareas domésticas, actividades familiares o realizadas durante el tiempo libre) | | | | |
|---|---|---|---|---|---|
| No tengo problemas para realizar mis actividades de todos los días | ☐ | Tengo algunos problemas para realizar mis actividades de todos los días | ☐ | Son incapaz de realizar mis actividades de todos los días | ☐ |

| d. | **Dolor/Malestar** | | | | |
|---|---|---|---|---|---|
| No tengo dolor ni malestar | ☐ | Tengo moderado dolor o malestar | ☐ | Tengo mucho dolor o malestar | ☐ |

| e. | **Ansiedad/Depresión** | | | | |
|---|---|---|---|---|---|
| No estoy ansioso/a ni deprimido/a | ☐ | Estoy moderadamente ansioso/a o deprimido/a | ☐ | Estoy muy ansioso/a o deprimido/a | ☐ |

Para ayudar a la gente a describir lo bueno o lo malo que es su estado de salud, hemos dispuesto una escala parecida a un termómetro en el cual se marca con un 100 el mejor estado de salud que pueda imaginarse, y con un 0 el peor estado de salud que pueda imaginarse.

**35.** Por favor, de 0 a 100, ¿cómo puntuaría **su estado de salud hoy?**

Peor estado de salud imaginable — 0 — 10 — 20 — 30 — 40 — 50 — 60 — 70 — 80 — 90 — 100 — Mejor estado de salud

**36.** Aquí están algunas cosas que otros pacientes nos han dicho sobre su dolor. Por favor, para cada afirmación marque una casilla del 0 al 6 para indicar hasta qué punto las actividades físicas tales como inclinarse, levantar peso, caminar o conducir afectan o afectarían a su dolor de espalda

| | | En total desacuerdo | | Ni en acuerdo ni desacuerdo | | | Totalmente de acuerdo | |
|---|---|---|---|---|---|---|---|---|
| | | 0 | 1 | 2 | 3 | 4 | 5 | 6 |
| 2 | La actividad física hace que mi dolor empeore | ☐ | ☐ | ☐ | ☐ | ☐ | ☐ | ☐ |
| 3 | La actividad física podría dañar mi espalda | ☐ | ☐ | ☐ | ☐ | ☐ | ☐ | ☐ |
| 4 | No debería hacer las actividades físicas que empeoran mi dolor, ni las que podrían empeorarlo | ☐ | ☐ | ☐ | ☐ | ☐ | ☐ | ☐ |
| 5 | No puedo realizar las actividades físicas que empeoran mi dolor, ni las que podrían empeorarlo | ☐ | ☐ | ☐ | ☐ | ☐ | ☐ | ☐ |

Las siguientes afirmaciones se refieren a cómo su trabajo normal afecta o afectaría a su dolor de espalda.

| | | En total desacuerdo | | Ni en acuerdo ni desacuerdo | | | Totalmente de acuerdo | |
|---|---|---|---|---|---|---|---|---|
| | | 0 | 1 | 2 | 3 | 4 | 5 | 6 |
| 6 | Mi dolor se debe a mi trabajo, o a un accidente en el trabajo | ▢ | ▢ | ▢ | ▢ | ▢ | ▢ | ▢ |
| 7 | Mi trabajo agravó mi dolor | ▢ | ▢ | ▢ | ▢ | ▢ | ▢ | ▢ |
| 8 | Mi trabajo es demasiado pesado para mí | ▢ | ▢ | ▢ | ▢ | ▢ | ▢ | ▢ |
| 9 | Mi trabajo empeora mi dolor, o podría empeorarlo | ▢ | ▢ | ▢ | ▢ | ▢ | ▢ | ▢ |
| 10 | Mi trabajo puede dañar mi espalda | ▢ | ▢ | ▢ | ▢ | ▢ | ▢ | ▢ |
| 11 | Con mi dolor actual no debería hacer mi trabajo normal | ▢ | ▢ | ▢ | ▢ | ▢ | ▢ | ▢ |
| 12 | No creo que pueda regresar a mi trabajo habitual en los próximos 3 meses | ▢ | ▢ | ▢ | ▢ | ▢ | ▢ | ▢ |

**37.** Indica cualquier otra CUESTIÓN, COMENTARIO U OBSERVACIÓN que consideres de interés en relación con los temas tratados en este cuestionario:

**MUCHAS GRACIAS POR TU COLABORACIÓN**

## ANEXO 3: PROCEDIMIENTO PARA LA APLICACIÓN DEL MÉTODO ERGOPAR

L’aplicació del mètode ERGOPAR té com a objectiu final la proposta i implementació de mesures de millora de les condicions de treball ergonòmiques de la/es unitat/s seleccionada/es a cada centre. Requereix d’una preparació, tant a nivell de grup com institucional, una infraestructura i condicions per a la seva implementació, unes accions i una visibilitat franca que és clau per a que els professionals de la/es unitat/es i la institució sigui conscient de les mesures implementades i que revertirà en una satisfacció a tots nivells.

En aquest document es detallen totes aquestes accions de preparació, implementació, seguiment i comunicació (taula 1), així com s’annexen els models de documents i documentació necessària en tot el procés (Documents).

**Documents**

3.1 Model d’Acord del Comitè de Seguretat i Salut del (nom institució) per a l’aplicació del mètode ERGOPAR.

3.2 Curs “Ergonomia en el ámbito sanitario y Método ERGOPAR”.

3.3 Document “Ergonomia laboral.doc”

3.4 Full de registre de problemes.

3.5 Rol i participació dels/de les referents de les unitats/clústers

3.6 Full de registre de solucions.

3.7 Model Informe propostes mesures de millora

3.8 Taula de planificació

**Taula 1. Accions, responsables i contingut/guió de l'aplicació del mètode ERGOPAR**

| FASE | ACCIÓ | Responsable/s | Contingut/guió | Documentació/Observacions |
|---|---|---|---|---|
| PREPARACIÓ | 1. Acord Comitè Seguretat i Salut (CSS) | Cap servei de salut laboral/servei de prevenció (SSL/SP), President CSS | Tot els membres del CSS (representants empresa i delegats de prevenció) han de signar el l'acord/compromís de suport a l'ERGOPAR de la/es unitat/s seleccionada/es (model annex 1). | Reunió més propera del Comitè de Seguretat i Salut. Idealment ha d'estar signat abans d'iniciar el procés ERGOPAR. Cal haver prèviament acordat prèviament amb la Direcció l'aplicació del mètode EROPAR fins al final. |
| | 2. Constitució del grup ERGO | Cap SSL/SP | El grup està format per:<br>• Comandament/s de la unitat<br>• Referents: treballadors de l'equip de la unitat a nivell dels 4 torns (matí, tarda, nit A, nit B). Habitualment proposats pel cap de la unitat. Han de ser líders del seu equip unitat-torn<br>• Expert SSL: ergònom/a<br>• Champion | Crear grup WhatsApp: referents, comandaments, ergònom, champion, cap SSL/SP (administrador). |
| | 3. Acreditació i reconeixement tasca referents | Formació continuada/Cap SSL/SP | Curs de formació, 20 hores de formació continuada acreditada per carrera professional. (annex 2) | Enviar proposta curs a Formació continuada. |
| | 4. Avaluació de riscos | Tècnic de prevenció assignat a la unitat | Actualització de l'avaluació de riscos laborals de la unitat. | Es lliura presencialment al comandament de la unitat, junt amb una explicació sobre el contingut de l'avaluació. |
| **IMPLEMENTACIÓ** (cont.) | 5. Reunió grup ERGO 1 | Ergònom, Champion | • Formació en ergonomia laboral (conceptes d'ergonomia, manipulació manual de càrregues i postures forçades) i participativa (mètode ERGOPAR)<br>• Avaluació de riscos<br>• Resultats qüestionaris basals<br>• Explicació del full de registre de problemes per consensuar amb els cercles de prevenció de cadascun del torns i que caldrà fer entrega complimentat pels participants del grup en la següent reunió. | Full de signatures per formació continuada (tots signen). Cada institució disposa del seu model<br>Documentació que s'entrega **1 setmana ABANS** de la reunió:<br>• Document "Ergonomia laboral.doc" Annex 3<br>• Full de registre de problemes. Annex 4<br>• Consentiment informat sobre la tasca de referent als Referents. Annex 5 |

| FASE | ACCIÓ | Responsable/s | Contingut/guió | Documentació/Observacions |
|---|---|---|---|---|
| IMPLEMENTACIÓ | 6. Cercles de prevenció | Referents | Discussió de cada referent amb tot el seu equip de la unitat-torn sobre els problemes ergonòmics de la unitat, en reunions informals a la mateixa unitat. | Emplenar el full de registre de problemes |
| | 7. Reunió grup ERGO 2 | Ergònom, Champion | Posta en comú dels problemes identificats a cadascun dels torns i elaboració del llistat de problemes. | Full de signatures per formació continuada (tots signen). Cada institució disposa del seu model<br>S'entrega el full de registre de les possible solucions Annex 6 |
| | 8. Llistat de problemes | Champion, Ergònom | Elaboració del document de problemes | S'envia als referents la versió final |
| | 9. Cercles de prevenció | Referents | Discussió en reunions informals amb equip unitat-torn les possibles solucions als problemes identificats | Emplenar el full de registre de solucions |
| | 10. Reunió grup ERGO 3 | Ergònom, Champion | Posta en comú de les solucions proposades a cadascun dels torns i elaboració del llistat de solucions, i priorització | Full de signatures per formació continuada (tots signen). Cada institució disposa del seu model |
| | 11. Document final de problemes, prioritats i solucions | Champion, Ergònom | Elaboració del document final "INFORME PROPUESTAS MEDIDAS DE MEJORA" (model annex 7) | S'envia la versió final a tot el grup ERGO. Pot haver-hi algunes rondes de comentaris fins a la versió final. |
| | 12. Presentació del document final de problemes, prioritats i solucions | Cap SSL/SP | Presentació del document final "INFORME PROPUESTAS MEDIDAS DE MEJORA" al Comitè de Seguretat i Salut. Afegir l'ERGOPAR a l'ordre del dia permanent del Comitè. | |
| **PLANIFICACIÓ, IMPLEMENTACIÓ I SEGUIMENT DE SOLUCIONS** (cont.) | 13. Grup operatiu | Cap SSL/SP | • Funció: planificar i implementar les mesures proposades (solucions) pel grup ERGO.<br>• Constitució: Cap SSL/SP, Cap Unitat, Ergònom, Cap Infermeria (segons hospital), Cap de serveis generals/hoteleria i altres, segons necessitats.<br>• Reunions periòdiques: habitualment mensuals, taula de planificació | Documentació:<br>• Taula de planificació (model annex 8)<br>• Registre del cost de les mesures implementades (pot estar inclòs a la taula de planificació) |

| FASE | ACCIÓ | Responsable/s | Contingut/guió | Documentació/Observacions |
|---|---|---|---|---|
| **PLANIFICACIÓ, IMPLEMENTACIÓ I SEGUIMENT DE SOLUCIONS** | 14. Informe final | Champion/Cap SSL/SP, grup ERGO | • Informe final de la implementació de mesures/solucions que s'elabora al final de la intervenció INTEVAL_Spain. Aquest informe ha de ser compartit i validat pel grup ERGO de la unitat.<br>• Presentació de l'informe final al CSS i a la Direcció, especialment la Direcció d'Infermeria. | Document: Informe **final** de la implementació de mesures/solucions (ERGOPAR INTEVAL_Spain). |
| | 15. Seguiment de la implementació de mesures/solucions fins al final i informe definitiu | Cap SSL/SP | El compromís de l'ERGOPAR, va més enllà del projecte INTEVAL_Spain i per tant cal seguir la planificació i implementació de mesures/solucions fins a esgotar la llista. Al final de tot caldrà realitzar l'informe definitiu, que serà igualment presentat al CSS i la Direcció. | Document: Informe **definitiu** de la implementació de mesures/solucions (ERGOPAR). |
| **COMUNICACIÓ** | 16. Comunicació la incorporació de mesures/solucions proposades pel grup ERGO | Champion | Informar periòdicament sobre l'estat actual de la implementació de les mesures de millora i cada vegada que s'implementi una solució, i feed-back dels referents i equip unitat:<br>• WhatsApp des de la coordinació (champion) als referents, i d'aquests a l'equip unitat-torn.<br>• Informació escrita (rètol) a l'espai reservat a INTEVAL_Spain del plafó/suro de la unitat. | |

- **MODEL D'ACORD DEL COMITÈ DE SEGURETAT I SALUT PER A L'APLICACIÓ DEL MÈTODE ERGOPAR.**

**Data de l'acord:**

**Assistents**

Membres del Comitè Seguretat i Salut:

| | |
|---|---|
| **Representants empresa del *[nom institució]*:** | |
| **Delegats/des de Prevenció del *[nom institució]*:** | |

**Propòsit de l'acord**

El propòsit de l'acord és aplicar el Mètode ERGOPAR al *[nom institució]*. El Mètode ERGOPAR permet, mitjançant un procediment participatiu, identificar problemes de naturalesa ergonòmica en l'empresa, prioritzar-los i proposar mesures preventives per a la seva correcció.

**Agents implicats en l'acord**

La direcció de l'empresa i els seus representants, i la representació legal dels treballadors, al Comitè de Seguretat i Salut.

**Objectiu**

Eliminar o reduir els trastorns musculoesquelètics d'origen laboral que poguessin afectar la salut dels treballadors de l'empresa mitjançant l'aplicació de mesures preventives específiques.

**Àmbit d'intervenció**

S'acorda la constitució de 3 unitats assistencials del *[nom institució]*, en les quals s'intervindrà de forma progressiva, amb un disseny abans - després.

Les unitats plantejades són: *[nom de la/es unitat/es intervenció]*. Cadascuna d'elles amb 36, 24 i 20. En total són 80 llocs de treball.

Les raons que justifiquen l'aplicabilitat del mètode en aquests llocs de treballs són diverses:

- [Exemple: UGA : detectat el risc de manipulació manual de càrregues (MMC), com a conseqüència de l'assistència a pacients complexes i amb alta dependència física i psíquica.]

**Participació**

A més de la implicació dels components del Grup Ergo, el Mètode ERGOPAR requereix participació a tots els nivells de l'empresa. L'aplicació del mètode requereix emplenar un qüestionari, entrevistes, observació de llocs de treball, reunions informatives i reunions de treball amb treballadors.

**Cronograma**

| Acció | Unitat | Data prevista |
|---|---|---|
| 1. Entrega i recollida de qüestionaris inicials a tots els treballadors/es de les respectives unitats | | |
| 2. Processament i anàlisi dels qüestionaris i elaboració de l'informe de resultats | | |
| 3. Randomització: selecció dels clústers d'intervenció i control | | |
| 4. Constitució del Grup Ergo l'encarregat de l'execució del Mètode ERGOPAR, garantint per part de la direcció de l'empresa que cadascun dels seus membres comptarà amb el suport necessaris per al seu avanç | | |
| 5. Sessió 1: Formació específica del Grup Ergo | | |
| 6. Sessió 2 i 3 reunió identificació problemes i solucions del Grup Ergo | | |
| 7. Constitució cercles de prevenció | | |
| 8. Presentació dels resultats del Grup Ergo i cercles de prevenció en el Comitè de Seguretat i Salut | | |
| 9. Constitució del Grup Operatiu. Planificació e implementació de les mesures proposades | | |
| 10. Entrega i recollida de qüestionaris post-intervenció grups d'intervenció i control | | |
| 11. Processament i anàlisi dels qüestionaris i elaboració de l'informe sobre l'avaluació de la intervenció | | |

**Signen:**

Membres del Comitè de Seguretat i Salut

- **CURS ERGONOMIA EN EL ÁMBITO SANITARIO Y MÉTODO ERGOPAR**

## TÍTULO DEL CURSO: "ERGONOMÍA EN EL ÁMBITO SANITARIO Y MÉTODO ERGOPAR"

**Servicio de Salud Laboral/Servicio de Prevención,** *[nom institució]*

**Barcelona,** *[data]*

**OBJETIVOS**

1. Introducir a un grupo de trabajadores del Parc de Salut Mar (PSMAR) expuestos a riesgos ergonómicos en los conceptos básicos de la ergonomía en el ámbito sanitario.
2. Capacitarlos para la identificación de riesgos ergonómicos en sus propios puestos de trabajo y la gestión y propuesta de medidas preventivas, utilizando la metodología ERGOPAR.

**METODOLOGÍA DOCENTE**

La formación desarrollará los conceptos básicos de ergonomía y la implementación del Método ERGOPAR, adaptado específicamente a las características propias del sector público-sanitario. La formación combinará sesiones teóricas en el aula, con seminarios prácticos.

Los asistentes adquirirán conocimientos sobre Ergonomía Participativa y obtendrán las pautas para la detección de riesgos ergonómicos y la propuesta y aplicación de medidas preventivas, así como los roles y las acciones que deberán llevar a cabo cada uno de los participantes.

Simultáneamente, adquirirán competencias para el desarrollo de dinámicas grupales: coordinación de los participantes, gestión de la información recibida a través de los diversos documentos que se les facilitarán y recopilación de información en cuanto a riesgos ergonómicos existentes dentro de las unidades hospitalarias

Número total de participantes: 16-20 personas. Cada acción formativa contará con el siguiente número de participantes:

- Sesiones teóricas: 10-20 participantes/sesión
- Seminarios prácticos: 5-7 participantes/sesión
- Trabajo de preparación antes y después de cada sesión

**DOCENTES**

- *[nom ergònom]*, Técnic/a de Prevención de Riesgos Laborales, especialista en Ergonomía. Servicio de Salud Laboral/Servicio de Prevencióbn, *[nom institució]*.

- *[nom champion]*, Centro de Investigación en Salud Laboral (CISAL), Universidad Pompeu Fabra (UPF).

**PROGRAMA**

Los temas que se tratarán, así como el tiempo de desarrollo, se detallan en la siguiente tabla:

| Sesión | Tipo de sesión | Contenido | Duración | Núm. participantes |
|---|---|---|---|---|
| 1 | Sesión teórica (x 2 grupos) | Conceptos básicos en Ergonomía en el trabajo Y Ergonomía participativa. | 1h<br>(x2 grupos) | 10-20 |
| 2 | Seminario (x 3 grupos) | Factores de riesgo ergonómicos y efectos sobre la salud en unidades de hospitalización de pacientes con semi y alta dependencia, y área quirúrgica. | 1 h.<br>(x3 grupos) | 5-7<br>(por grupo) |
| 3 | Seminario (x 3 grupos) | Pautas y medias preventivas para reducir los riesgos ergonómicos y efectos sobre la salud en unidades de hospitalización de pacientes con semi y alta dependencia, y área quirúrgica. Elaboración de un plan de trabajo para eliminar o reducir los riesgos ergonómicos. | 1 h.<br>(x3 grupos) | 5-7<br>(por grupo) |
| 4 | Trabajo de preparación antes y después de cada sesión | El trabajo de preparación consiste en la revisión de la documentación, los comentarios a la documentación antes y después de cada sesión, y para los seminarios además el trabajo de discusión con sus equipos antes y después también de cada sesión. | • 2h. sesión teórica<br>• 5h para cada seminario | 5-7<br>(por grupo) |

Cada alumno participará en un total de 20 h:

- 1h de teoría
- 2h de seminarios prácticos
- 17h de preparación antes y después de cada sesión

**CALENDARIO**

Se organizarán tres grupos:

- A: equipo de enfermería (enfermeros/as y auxiliares) UH30, H Mar
- B: equipo de enfermería (enfermeros/as y auxiliares) UCI, H Mar

- C: equipo de enfermería (enfermeros/as y auxiliares) LLevants 3 y 4, CAEM

De acuerdo al siguiente calendario:

| **Sesión** | **Grupo** | **Fecha** | **Horario** | **Aula** |
|---|---|---|---|---|
| Teórica | A+B | | | |
| Teórica | C | | | |
| Seminario 1 | A | | | |
| Seminario 1 | B | | | |
| Seminario 1 | C | | | |
| Seminario 2 | A | | | |
| Seminario 2 | B | | | |
| Seminario 2 | C | | | |

**EVALUACIÓN**

Cada participante realizará una prueba escrita tipo test, de 30 preguntas, sobre los conceptos impartidos durante la formación.

Mediante esta prueba se evaluarán los conocimientos y las competencias en Ergonomía y en prevención de riesgos ergonómicos dentro del ámbito sanitario, adquiridas por los participantes.

**RECURSOS DIDÁCTICOS**

Los materiales didácticos que se utilizarán, así como la documentación necesaria para el desarrollo de las diferentes sesiones formativas serán puestos a disposición de los participantes, para su estudio previo, antes de cada sesión.

- **PRESENTACIÓN “ERGONOMIA LABORAL”**

# PROYECTO INTEVAL

# Intervención: ERGONOMÍA PARTICIPATIVA

↓

# GRUPO ERGO

# Sesión 1: Ergonomía Laboral

# Índice

1. Ergonomía Laboral
2. Introducción a la Ergonomía Participativa
3. El Método ERGOPAR
4. Material de apoyo: Presentación

# 1. ERGONOMÍA LABORAL

**Objetivos:**

- Contar con una base teórica común en ergonomía.
- Entender las situaciones de riesgo ergonómicas en el contexto laboral.
- Capacitar para la identificación de causas de exposición a factores de riesgo ergonómicos.
- Capacitar para la propuesta de medidas preventivas.

---

## 1. Conceptos y términos sobre ergonomía

### 1.1. Definiciones de ergonomía

El término ergonomía proviene de las palabras griegas ERGON (Trabajo) y NOMOS (Ley o Norma).

La utilización moderna del término se debe a Murell y fue adoptado oficialmente en julio 1949, a raíz de la creación de la primera Sociedad de ergonomía, la "Ergonomics Research Society" con el fin de "adaptar el trabajo al hombre".

Según la enciclopedia Larousse, se define Ergonomía como el estudio cuantitativo y cualitativo de las condiciones de trabajo en la empresa, que tiene como objeto el establecimiento de técnicas conducentes a una mejora de la productividad y la integración en el trabajo de los productores directos.

Entre las definiciones de los profesionales de la ergonomía encontramos, además de la anteriormente mencionada de Murell, otras como las de Singleton (1969), Faverge (1970), Cazamian (1973), McCormick (1981), Pheasant (1988). De éstas se desprenden tres cuestiones:

- Su principal sujeto de estudio es el hombre en interacción con el medio.
- Su estatus de ciencia normativa.
- Su vertiente de protección de la salud de las personas (física, psíquica y social).

En resumen, podemos decir que la ergonomía trata de alcanzar el mayor equilibrio posible entre las necesidades/posibilidades del usuario y las prestaciones/requerimientos de los productos y servicios.

### 1.2. Objetivo de la Ergonomía

El objetivo de la ergonomía es mejorar la “calidad de vida” y bienestar de los usuarios, reduciendo los riesgos de error. No se limita a identificar los factores de riesgo y las molestias, sino que propone soluciones positivas, soluciones que se mueven en el ámbito posibilista de las potencialidades efectivas de los usuarios, y de la viabilidad económica de cualquier proyecto.

## 2. Daños a la salud por trastornos musculoesqueléticos

Los trastornos musculoesqueléticos (TME) son una de las enfermedades de origen laboral más comunes que afectan a millones de trabajadores en toda Europa y cuestan a las empresas miles de millones de euros. Afrontar los TME ayuda a mejorar las vidas de los trabajadores, de la empresa y de la sociedad.

Los trastornos musculoesqueléticos normalmente afectan a la espalda, cuello, hombros y extremidades superiores, aunque también pueden afectar a las extremidades inferiores. Comprenden cualquier daño o trastorno de las articulaciones y otros tejidos. Los problemas de salud abarcan desde pequeñas molestias y dolores, que son los más frecuentes, a cuadros médicos más graves que precisan incapacidad temporal (IT o bajas), e incluso a recibir tratamiento médico. En los casos más crónicos, pueden dar como resultado una discapacidad y la necesidad de dejar de trabajar.

La mayoría de los TME relacionados con el trabajo se desarrollan a lo largo del tiempo. Normalmente no hay una única causa de los TME, sino que son varios los factores que trabajan conjuntamente. Entre las causas físicas y los factores de riesgos organizativos se incluyen:

- Manipulación de cargas, especialmente al agacharse y girarse.
- Movimientos repetitivos o forzados.
- Posturas extrañas o estáticas.
- Vibraciones, iluminación deficiente o entornos de trabajo fríos.
- Trabajo a un ritmo elevado.
- Estar de pie o sentado durante mucho tiempo en la misma posición.

Existen datos crecientes que vinculan los trastornos musculoesqueléticos con factores de riesgo psicosocial (en especial combinados con riesgos físicos), entre los que se incluyen:

- Alto nivel de exigencia de trabajo o una escasa autonomía.
- Escasa satisfacción laboral.

## 3. El riesgo ergonómico: factores de riesgo y sus causas de exposición

En términos generales, se entiende como "riesgo" la probabilidad de sufrir un determinado evento. Siendo un dato probabilístico, también puede ser entendido como el número de personas de una población que serán afectados por una condición particular.

Serán factores de riesgo ergonómico aquel conjunto de atributos de la tarea o del puesto, más o menos claramente definidos, que inciden en aumentar la probabilidad de que un sujeto, expuesto a ellos, desarrolle una lesión en su trabajo.

Los estudios de campo desarrollados por la OSHA (Occupational Safety and Health Administration), en los Estados Unidos, han permitido establecer la existencia de 5 riesgos que se asocian estrechamente con el desarrollo de lesiones musculoesqueléticas:

1) Desempeñar el mismo movimiento o patrón de movimientos cada varios segundos durante más de dos horas ininterrumpidas.
2) Mantener partes del cuerpo en posturas fijas o posturas peligrosas durante más de dos horas en un turno de trabajo.
3) La utilización de herramientas que producen vibración durante más de dos horas de trabajo.
4) Realizar esfuerzos vigorosos durante más de dos horas de trabajo.
5) Hacer levantamiento manual frecuente o con sobreesfuerzo.

Relación Causa-Efecto: existe mucha evidencia epidemiológica que demuestra una fuerte asociación entre el desarrollo de lesiones músculo tendinosas y determinados factores físicos del trabajo, especialmente cuando existe un alto nivel de exposición y además la exposición es a una combinación de varios factores.

## 4. Medidas preventivas frente al riesgo ergonómico

Las estrategias de prevención de los trastornos músculoesqueléticos se basan en intervenciones centradas en el lugar de trabajo y en factores individuales. Cada vez se reconoce en mayor medida la necesidad de adoptar un enfoque integrado que incluya ambos tipos de intervención para tratar el problema con eficacia. Las intervenciones ergonómicas se basan en un enfoque «holístico» o sistémico que considera a la vez tanto el efecto del equipo, del entorno laboral y de la organización del trabajo como al trabajador. También es importante el hecho de que los trabajadores participen activamente en su adopción.

Mediante la prevención se pretende:

- Evitar los riesgos.
- Evaluar los riesgos que no se puedan evitar.
- Combatir los riesgos en su origen.
- Adaptar el trabajo a la persona, en particular en lo que respecta a la concepción de los puestos de trabajo, así como a la elección de los equipos de trabajo y los Métodos de trabajo y de producción, centrándose especialmente en atenuar el trabajo monótono y el trabajo repetitivo y a reducir los efectos de los mismos en la salud.
- Tener en cuenta la evolución de la técnica.
- Sustituir lo peligroso por lo que entraña poco o ningún peligro.
- Planificar la prevención buscando integrar, de manera coherente, la técnica, la organización del trabajo, las condiciones de trabajo, las relaciones sociales y la influencia de los factores ambientales en el trabajo.
- Adoptar medidas que antepongan la protección colectiva a la individual.
- Dar las debidas instrucciones a los trabajadores.

Las principales estrategias de prevención cubren tanto las estrategias de prevención primaria (eliminación de las causas) como de prevención secundaria (tratamiento y rehabilitación). En este caso también, las opiniones expertas señalan que, si bien debe hacerse especial hincapié en la prevención primaria, es preciso examinar todos estos factores con una visión de conjunto. Por ejemplo, los estudios muestran que es improbable que, por sí sola, la formación sea efectiva si persiste la deficiencia de los factores ergonómicos en el trabajo y que, por ejemplo, es preciso que la formación incluya cómo detectar riesgos potenciales y qué hacer una vez se detectan, así como técnicas seguras de manipulación física.

## 2. INTRODUCCIÓN A LA ERGONOMIA PARTICIPATIVA

---

### ***Índice***

- ¿Qué es la Ergonomía Participativa?
- ¿Por qué la ergonomía participativa en las empresas?
- Objetivos de la ergonomía Participativa

---

**¿Qué es la Ergonomía Participativa?**

No existe una única definición para el término Ergonomía Participativa. Son muchos los autores que han publicado su percepción y punto de vista en revistas científicas y especializadas, al igual que estudios de investigación y revisión que analizan los procedimientos desarrollados y las experiencias implementadas en empresas. Los términos que vienen repitiéndose en las distintas definiciones de ergonomía participativa son: participación, trabajadores, conocimientos, procedimientos, apoyo, mejora, actores, soluciones, implicar e influir. Estas palabras clave permiten unificar el enfoque y la manera de hacer que persigue la ergonomía participativa.

**La ergonomía participativa como una estrategia para la mejora de las condiciones de trabajo a nivel ergonómico, estructurada y ordenada por medio de un procedimiento que incorpora la participación activa de los agentes sociales implicados**.

**¿Por qué aplicar la ergonomía participativa en las empresas?**

Las ventajas que ofrecen este tipo de procedimientos, tanto para los trabajadores como para el conjunto de la empresa. Entre otras:

- Promueven la **participación** de los distintos actores en la empresa en la mejora de las condiciones de trabajo.
- Abordan una de las categorías de riesgos laborales que mayor **impacto tiene sobre la salud y el bienestar de los trabajadores**, previniendo su exposición y una de las principales causas de baja laboral.
- Permiten **identificar y tratar muchas situaciones de riesgo** sin necesidad de emplear complicados protocolos técnicos. Potencian la integración de la prevención en la empresa, facilitando la creación de una sólida cultura organizacional preventiva en la empresa.

Organismos como el Health and Safety Executive (HSE, Reino Unido), el Institut de Recherche Robert-Sauvé en Santé et Securité du Travail (IRSST, Québec-Canadá), el Institute for Work and Health (IWH, Ontario-Canadá), la Agencia Europea para la Seguridad y la Salud en el Trabajo (EU-OSHA, estados miembros de la Unión Europea) y el National Institute for Occupational Safety and Health (NIOSH, Estados Unidos), considerados todos ellos organismos e instituciones de referencia internacional en materia de seguridad y salud laboral, han desarrollado procedimientos, revisiones o publicado experiencias en ergonomía participativa de gran calidad, en los que destacan su apuesta por el desarrollo de este tipo de programas. Esta evidencia científica y el apoyo institucional, justifica el interés por desarrollar experiencias de ergonomía participativa en España, donde al igual que en otros países, los trastornos musculoesqueléticos son la patología de origen laboral más prevalente en la población trabajadora (sea considerada o no contingencia profesional), y cuya incidencia persiste año tras año mostrando las carencias del control efectivo del riesgo, en gran parte de las empresas.

**Objetivo de la Ergonomía Participativa**

El objetivo de la ergonomía participativa es la mejora de las condiciones de trabajo a nivel ergonómico y la prevención de los trastornos musculoesqueléticos.

La literatura nos muestra cual debe ser el objetivo de la ergonomía participativa. Aquí tenemos algunos ejemplos:

"Incrementar los conocimientos y la conciencia de los trabajadores respecto a la ergonomía en su puesto de trabajo, motivándolos a participar activamente en el programa de intervención, y en la aplicación de medidas ergonómicas en el centro de trabajo"

"Proporcionar a empresarios y trabajadores con información y asesoramiento un marco genérico (directrices) que les permita prevenir los trastornos musculoesqueléticos (TME) en su lugar de trabajo, y aplicar un programa y materiales efectivos e integrar la prevención de los TME en la organización de la empresa"

El objetivo goza de un mayor consenso, ya que la meta común coincide en que debe ser **la mejora de las condiciones de trabajo a nivel ergonómico y la prevención de los trastornos musculoesqueléticos.**

## 3. METODOLOGÍA ERGOPAR

***Índice***

- El Método Ergopar, ¿en qué consiste?
- Fases del Método Ergopar
- Fase Intervenció
    - Identificación y análisis
    - Propuesta y planificación
    - Seguimiento

### El Método Ergopar, ¿en qué consiste?

El Método ERGOPAR es un procedimiento de ergonomía participativa para la prevención del riesgo ergonómico de origen laboral. Se ha concebido para identificar la exposición a factores de riesgo ergonómicos a consecuencia del trabajo y sus causas de exposición, consensuar las mejores medidas preventivas para la eliminación o al menos, reducción de las situaciones de riesgo, implementarlas y realizar su seguimiento y mejora continua. **(http://Ergopar.istas.net/)**

El Método Ergopar tiene un doble objetivo:

- La mejora continua de las condiciones de trabajo a nivel ergonómico mediante la implementación de medidas preventivas que eliminen o al menos, reduzcan la exposición a factores de riesgo, y
- facilitar la participación de los trabajadores, sus representantes legales y demás actores implicados en la prevención de riesgos laborales en la empresa.

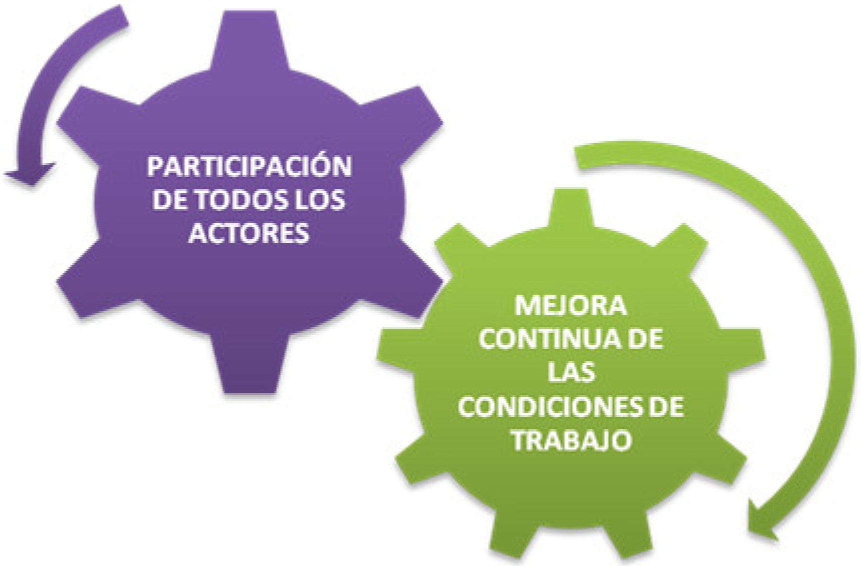


Las ventajas de llevar a cabo un procedimiento de ergonomía participativa siguiendo el Método ERGOPAR son muchas, entre ellas destacamos las siguientes:

- Incorpora el conocimiento y experiencia de todas las personas implicadas en la experiencia, destacando el proporcionado por los trabajadores que pasan a integrarse en las actuaciones preventivas en la empresa.
- Genera dinámicas y habilidades participativas y grupales que permanecen en la empresa. La principal ventaja de la ergonomía participativa es que se crea en la empresa "un saber hacer en prevención". Mediante la formación y la experiencia, por ejemplo, los miembros del Grupo Ergo adquieren destrezas que les permiten analizar y resolver situaciones de riesgo ergonómico. Este conocimiento y las habilidades participativas en las que se ven inmersos son fundamentales para promover la continuidad del programa y la integración de la prevención de riesgos laborales en la empresa.
- Facilita la propuesta de medidas preventivas adaptadas a las necesidades y circunstancias reales de la empresa y los trabajadores. Las medidas preventivas a aplicar son propuestas, planificadas e implementadas por el personal de la empresa, lo que permite su mejor integración en las prácticas internas, respetando las peculiaridades culturales, económicas y técnicas del centro de trabajo.
- Permite flexibilidad y adaptación a los distintos contextos empresariales.
- Favorece la aceptación de las medidas preventivas. La resistencia al cambio se atenúa e incluso, desaparece en la medida en que los propios afectados participan en la

identificación de factores de riesgo ergonómico y daños de origen laboral, en la propuesta de medidas preventivas y en su seguimiento.

- Acorta los tiempos de implementación de las medidas preventivas. En base a los recursos disponibles en la empresa (económicos, tiempo, disponibilidad…) es posible acortar los plazos hasta la ejecución de las medidas preventivas.
- Permite visualizar la implicación de la dirección de la empresa en la prevención de los riesgos ergonómicos. Esta implicación, voluntariedad y compromiso firme de la dirección se visualiza desde el primer momento en el que se acuerda la aplicación del Método.
- Promueve la mejora de las relaciones laborales en el centro de trabajo. El proceso participativo mejora la comunicación en la empresa y permite argumentar y aproximar las distintas perspectivas sobre las situaciones de riesgo ergonómico y el consenso de las medidas preventivas a implementar, facilitando el cumplimiento de derechos básicos de los trabajadores y sus representantes legales.

**Fases del Método Ergopar**

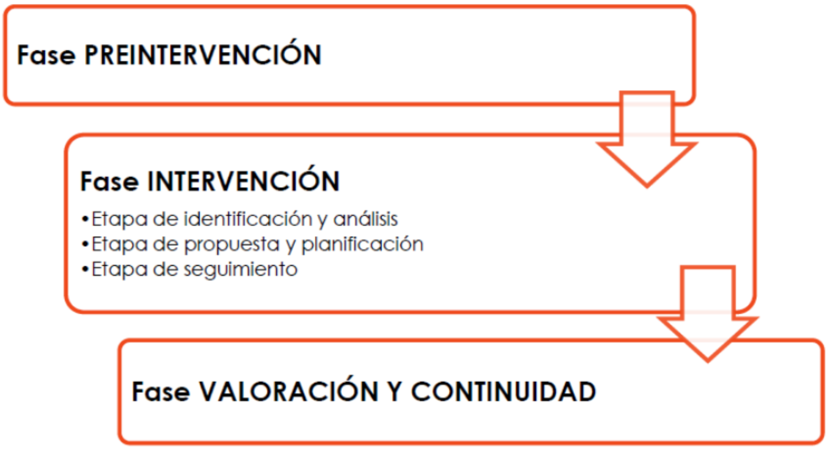


**Fase de intervención**

- **Identificación y análisis**

El objetivo de esta etapa es identificar exposiciones a factores de riesgo ergonómicos y daños prioritarios en los puestos de trabajo por colectivo homogéneo, para después identificar las causas de exposición a dichos factores de riesgo.

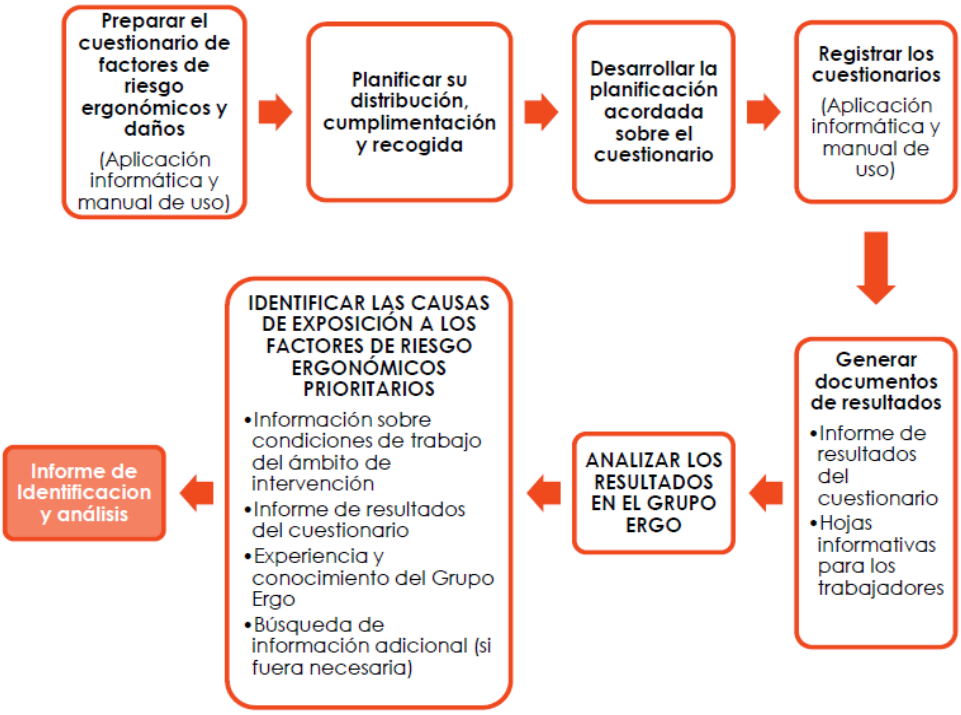


*(relacionado cuestionario Ergopar + Evaluación de la UH)*

La etapa de identificación y análisis supone una parte importantísima en el conjunto del procedimiento, dado que de la información que aquí se genere, dependerá la propuesta de medidas preventivas. Una inadecuada campaña informativa (sensibilización), una deficiente distribución o cumplimentación del cuestionario, o una falta de dedicación o conocimientos por parte del Grupo Ergo en la identificación de causas, puede poner en peligro el éxito de la experiencia participativa, generando una información de escasa representatividad y poco realista.

- **Propuesta y Planificación**

El objetivo de la etapa de propuesta y planificación es obtener una propuesta de medidas preventivas consensuadas y priorizadas, que elimine o al menos reduzca la exposición a las situaciones de riesgo ergonómico identificadas (factores de riesgo y sus causas de exposición), y acordar su planificación e implementación en el ámbito de intervención.

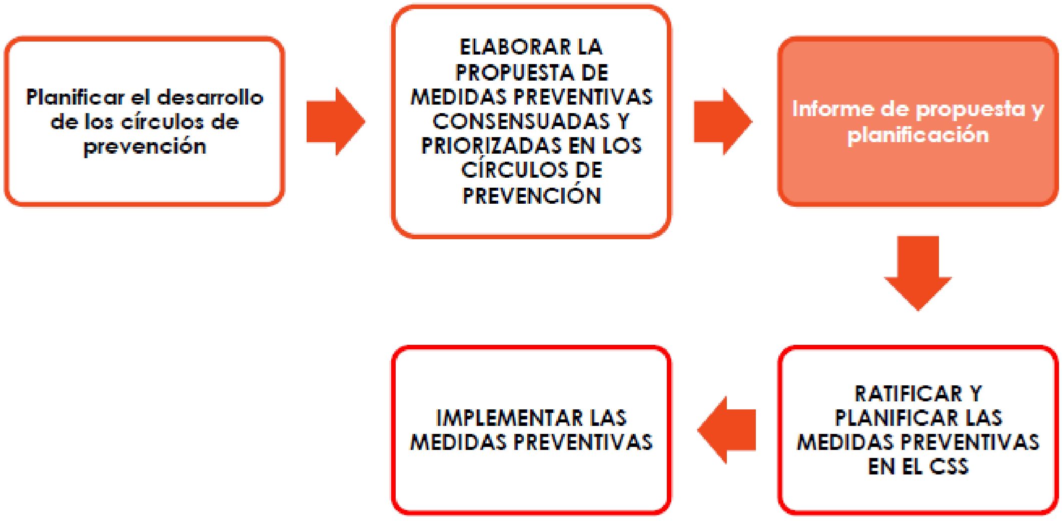


Para el logro de este objetivo, el Método ERGOPAR proporciona herramientas que favorecen la consecución de las tareas. Son las siguientes: En la búsqueda de medidas preventivas se empleará un guión para el desarrollo de círculos de prevención. En la elaboración de la propuesta de medidas preventivas consensuada y priorizada se utilizará, una ficha de trabajo. Para la planificación de las medidas preventivas a ejecutar se facilita una ficha de planificación a integrar en el documento de planificación de acciones preventivas de la empresa.

- **Seguimiento**

La etapa de seguimiento tiene un doble objetivo: controlar que se cumple en tiempo y forma la ejecución de la planificación de medidas preventivas acordadas y evaluar la eficacia de las medidas preventivas implementadas y en su caso, corregir su ineficacia para la mejora continua.

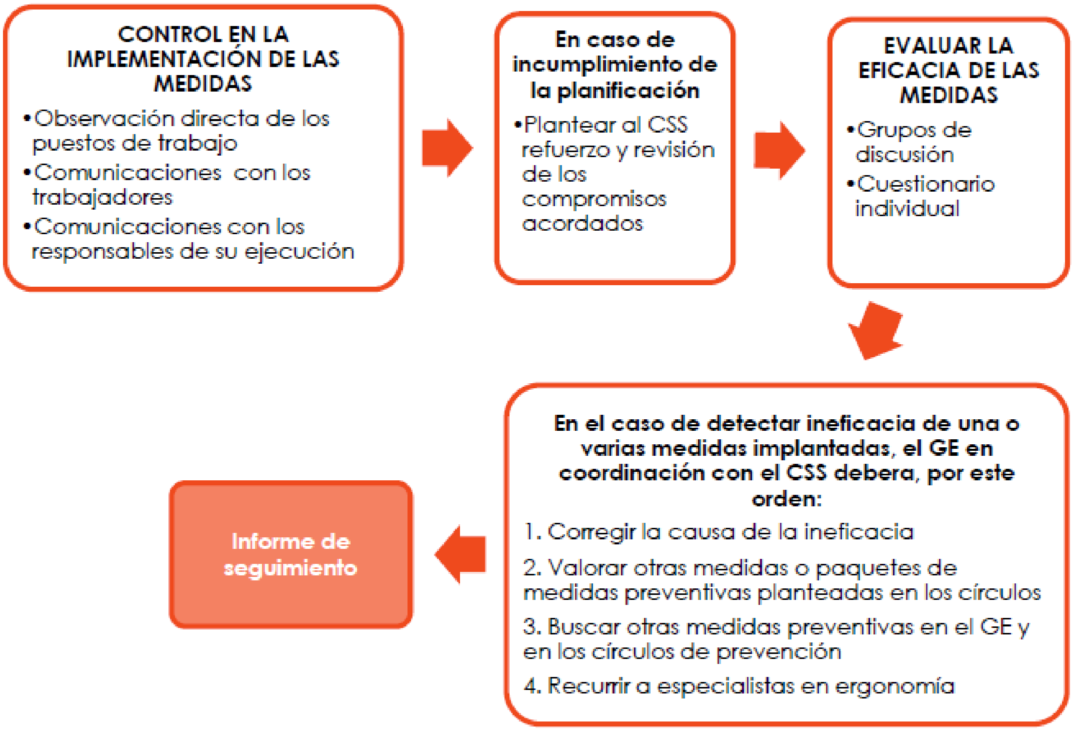
CONTROL EN LA IMPLEMENTACIÓN DE LAS MEDIDAS
•Observación directa de los puestos de trabajo
•Comunicaciones con los trabajadores
•Comunicaciones con los responsables de su ejecución
En caso de incumplimiento de la planificación
•Plantear al CSS refuerzo y revisión de los compromisos acordados
EVALUAR LA EFICACIA DE LAS MEDIDAS
•Grupos de discusión
•Cuestionario individual
En el caso de detectar ineficacia de una o varias medidas implantadas, el GE en coordinación con el CSS debera, por este orden:
1. Corregir la causa de la ineficacia
2. Valorar otras medidas o paquetes de medidas preventivas planteadas en los círculos
3. Buscar otras medidas preventivas en el GE y en los círculos de prevención
4. Recurrir a especialistas en ergonomía
Informe de seguimiento

**LOGO HOSPITAL**

- **HOJA DE REGISTRO DE PROBLEMAS**

**UNIDAD:**

| | | **FACTORES DE RIESGO ERGONÓMICO** | | | | | | | |
|---|---|---|---|---|---|---|---|---|---|
| | | **Manipulación de cargas/pacientes** | | | | | **Postura Forzada** | | |
| Tarea/Actividad | Descripción de la actividad/tarea | Características de la carga | Esfuerzo físico necesario | Características del medio de trabajo | Exigencias de la actividad | Factores individuales de riesgo | Frecuencia de movimientos | Duración de la postura | Postura |
| | | | | | | | | | |
| | | | | | | | | | |

- **ROL Y PARTICIPACIÓN DE LOS/LAS REFERENTES DE LAS UNIDADES/CLÚSTERS**

## PROYECTO INTEVAL_Spain: ROL Y PARTICIPACIÓN DE LOS/LAS REFERENTES DE LAS UNIDADES/*CLÚSTERS*

La intervención del proyecto INTEVAL_Spain consta de tres componentes: ergonomía participativa (EP), gestión de casos y Promoción de la saud. La EP precisa del grupo ERGO, que se compone del/la coordinadora, expertos/as en ergonomía, jefes/as y supervisores/as de las unidades participantes y un grupo de 4-6 profesionales de cada unidad que representen los diferentes turnos. Estos/as profesionales son los/as REFERENTES.

Las funciones de los/as referentes van más allá del grupo ERGO. Tienen, además, un rol dinamizador dentro de su grupo (turno) en la unidad a lo largo del proyecto y de puente de comunicación entre el resto de trabajadores/as de las unidades/*clústers* participantes y la coordinación del proyecto.

**Estas funciones son:**

1. Entender las situaciones de riesgo ergonómico en el contexto laboral y sus causas, y proponer mejoras de las condiciones de trabajo.
2. Promover la intervención de EP involucrando al resto de profesionales de su unidad/*clúster* y turno en la detección de situaciones de riesgo ergonómico y propuesta de medidas de mejora.
3. Mantener la comunicación con la coordinación del proyecto INTEVAL_Spain, a través del Whatsapp y/o el correo electrónico, y con el equipo de profesionales de su unut¡dad/turno.
4. Consultar y comentar los materiales formativos que serán puestos a su disposición por el Equipo Técnico INTEVAL_Spain durante las sesiones del grupo ERGO.
5. Acudir a las reuniones previstas y participar de manera activa, transmitiendo la información recopilada en la unidad/*clúster* y aportando las observaciones que crean oportunas.
6. Recopilar información detallada sobre las condiciones de trabajo de la unidad/*clúster*.
7. Promover el intercambio de ideas y la innovación entre los/las profesionales de la unidad/*clúster* para la obtención de propuestas de medidas preventivas.
8. Promover y facilitar la comunicación y el consenso entre los profesionales de la unidad/*clúster* durante toda la intervención.
9. Mantener un circuito de comunicación activa y fluida con los/las jefes/as y supervisores/as de la unidad/*clúster*, transmitiéndoles a la mayor brevedad posible cualquier sugerencia, duda o problema que pueda surgir durante el transcurso de la intervención.
10. Colaborar con la coordinación del proyecto INTEVAL_Spain en la organización de actividades que tengan relación con la unidad/*clúster* que representan a lo largo del proyecto.

- **HORAS DE FORMACIÓN PROFESIONAL POR EL CURSO DE ERGONOMIA PARTICIPATIVA**

**HORAS DE FORMACIÓN PROFESIONAL POR EL CURSO: "ERGONOMÍA EN EL ÁMBITO SANITARIO Y MÉTODO ERGOPAR"**

**Servicio de Salud Laboral, Parc de Salut Mar**

**Barcelona, abril de 2019**

**OBJETIVOS**

3. Introducir a un grupo de trabajadores del Parc de Salut Mar (PSMAR) expuestos a riesgos ergonómicos en los conceptos básicos de la ergonomía en el ámbito sanitario.
4. Capacitarlos para la identificación de riesgos ergonómicos en sus propios puestos de trabajo y la gestión y propuesta de medidas preventivas, utilizando la metodología ERGOPAR.

**METODOLOGÍA DOCENTE**

La formación desarrollará los conceptos básicos de ergonomía y la implementación del Método ERGOPAR, adaptado específicamente a las características propias del sector público-sanitario. La formación combinará sesiones teóricas en el aula, con seminarios prácticos.

Los asistentes adquirirán conocimientos sobre Ergonomía Participativa y obtendrán las pautas para la detección de riesgos ergonómicos y la propuesta y aplicación de medidas preventivas, así como los roles y las acciones que deberán llevar a cabo cada uno de los participantes.

Simultáneamente, adquirirán competencias para el desarrollo de dinámicas grupales: coordinación de los participantes, gestión de la información recibida a través de los diversos documentos que se les facilitarán y recopilación de información en cuanto a riesgos ergonómicos existentes dentro de las unidades hospitalarias

Número total de participantes: 16-20 personas. Cada acción formativa contará con el siguiente número de participantes:

- Sesiones teóricas: 10-20 participantes/sesión
- Seminarios prácticos: 5-7 participantes/sesión
- Trabajo de preparación antes y después de cada sesión

**DOCENTES**

- Chelo Sancho, Técnic/a de Prevención de Riesgos Laborales, especialista en Ergonomía. Servicio de Salud Laboral, Parc de Salut Mar
- Olga Martínez, Champion proyecto; Centro de Investigación en Salud Laboral (CISAL), IMIM-UPF.

**PROGRAMA**

Los temas que se tratará cada grupo, así como el tiempo de desarrollo, se detallan en la siguiente tabla:

| Sesión | Tipo de sesión | Contenido | Duración | Núm. participantes |
|---|---|---|---|---|
| 1 | Sesión teórica | Conceptos básicos en Ergonomía en el trabajo Y Ergonomía participativa. | 1h | 10-20 |
| 2 | Seminario | Factores de riesgo ergonómicos y efectos sobre la salud en unidades de hospitalización de pacientes con semi y alta dependencia, y área quirúrgica. | 1 h | 5-7 |
| 3 | Seminario | Pautas y medias preventivas para reducir los riesgos ergonómicos y efectos sobre la salud en unidades de hospitalización de pacientes con semi y alta dependencia, y área quirúrgica. Elaboración de un plan de trabajo para eliminar o reducir los riesgos ergonómicos. | 1 h | 5-7 |
| 4 | Trabajo de preparación antes y después de cada sesión | El trabajo de preparación consiste en la revisión de la documentación, los comentarios a la documentación antes y después de cada sesión, y para los seminarios además el trabajo de discusión con sus equipos antes y después también de cada sesión. | • 2h. sesión teórica<br>• 5h para cada seminario | 5-7 |

Cada alumno participará en un total de 20 h:

- 1h de teoría
- 2h de seminarios prácticos
- 17h de preparación antes y después de cada sesión

**CALENDARIO**

Se organizarán tres grupos:

- A: equipo de enfermería (enfermeros/as y auxiliares): **UH30, Hospital de l'Esperança.**
- B: equipo de enfermería (enfermeros/as y auxiliares): **UH07, Hospital del Mar.**

El calendario y horarios de cada grupo se muestra en la siguiente tabla.

| Sesión | Grupo | Fecha | Horario | Aula |
|---|---|---|---|---|
| Teórica | A | 08/05/2019 | 15:30-17:00 | Sala docent 7ªpl. H. de la Esperança |
| Teórica | B | 08/05/2019 | 14:00-15:30 | Sala2, 10ªPl. H.del Mar |
| Seminario 1 | A | 22/05/2019 | 15:30-17:00 | Sala polivalente 4ªpl. H. de la Esperança |
| Seminario 1 | B | 22/05/2019 | 14:00-15:30 | Sala 2, 10ªPl. H.del Mar |
| Seminario 2 | A | 29/05/2019 | 15:30-17:00 | Sala polivalente 4ªpl. H. de la Esperança |
| Seminario 2 | B | 29/05/2019 | 14:00-15:30 | Sala 2, 10ªPl. H.del Mar |

## EVALUACIÓN

Cada participante realizará una prueba escrita tipo test sobre los conceptos impartidos durante la formación.

Mediante esta prueba se evaluarán los conocimientos y las competencias en Ergonomía y en prevención de riesgos ergonómicos dentro del ámbito sanitario, adquiridas por los participantes.

## RECURSOS DIDÁCTICOS

Los materiales didácticos que se utilizarán, así como la documentación necesaria para el desarrollo de las diferentes sesiones formativas serán puestos a disposición de los participantes, para su estudio previo, antes de cada sesión.

- **CONSENTIMIENTO INFORMADO**

**DECLARACIÓN DE PARTICIPACIÓN VOLUNTARIA COMO PROFESIONAL REFERENTE DEL PROYECTO INTEVAL_Spain:**

Declaro que he sido informado/a de la naturaleza y el propósito de la fase de EP, de los datos que se me pide que proporcione y de las acciones que llevaré a cabo mediante mi participación como referente de la unidad/*clúster* a la que represento. He recibido una explicación satisfactoria sobre los procedimientos de la fase de EP y su finalidad.

Comprendo que mi decisión de participar es voluntaria y doy mi consentimiento para la participación en las acciones que se realizarán durante el desarrollo de la fase de EP. Conozco mi derecho a retirar este consentimiento cuando lo desee, con la única obligación de informar mi decisión, a la mayor brevedad posible, al Equipo Investigador INTEVAL_ plus.

Por favor, si ACEPTA PARTICIPAR voluntariamente en la fase de EP del Proyecto INTEVAL_Spain, firme a continuación e indíquenos su nombre y apellidos, así como la fecha actual.

Firma trabajador/a:

Nombre trabajador/a:

Fecha:

Firma de la persona que obtiene el consentimiento:

(Nombre)

Fecha:

LOGO HOSPITAL

- **FULL DE REGISTRE POSSIBLES SOLUCIONS**

**UNIDAD:**

| FICHA DE PROPUESTA DE MEDIDAS PREVENTIVAS PARTICIPANTES DEL "CERCLES DE PREVENCIÓ" | | | |
|---|---|---|---|
| **PUESTO DE TRABAJO:** | | | |
| **TAREA** | **CAUSAS** | **ESCALA** | **MEDIDAS PREVENTIVAS** |
| | | | |
| | | | |
| | | | |

| LOGO HOSPITAL |
|---|

- **INFORME PROPUESTAS MEDIDAS DE MEJORA**

# INFORME PROPUESTAS MEDIDAS DE MEJORA

**INFORMACIÓN DIRIGIDA A LOS "CERCLES DE PREVENCIÓ"**

| **FICHA INFORMACIÓN DE IDENTIFICACIÓN DE CAUSAS FACTORES RIESGOS ERGONOMICOS** | |
|---|---|
| Unidad: | Puesto: |
| Tareas/situaciones seleccionadas (orden de prioridad de 1 a 5)<br><br>1-<br>2-<br>3-<br>4-<br>5- | |
| **CLASIFICACIÓN** | **CAUSAS DE EXPOSICIÓN A LOS FACTORES DE RIESGOS** |
| Organización de trabajo | |
| Equipos y mobiliario | |
| Materiales y productos | |
| Entorno de trabajo y condiciones ambientales | |
| Tipología paciente | |
| Ropa de trabajo | |

- **TAULA DE PLANIFICACIÓ (FORMATO EXCEL)**

**LOGO HOSPITAL**

**ERGONOMIA PARTICIPATIVA, GRUP HOSPITAL . TAULA PLANIFICACIÓ - GRUP OPERATIU**

**Data reunió** XXX

**Participen:** XXXX

| ID | Mesura general | Mesura general, descripció | Tipus proposta | Proposta | Proposta descripció | Factibilitat | Responsables | Previsió | Cost afegit | Estat actual | Seguiment |
|---|---|---|---|---|---|---|---|---|---|---|---|
| | | | | | | | | | | | |
| | | | | | | | | | | | |
| | | | | | | | | | | | |

## ANEXO 4: CUESTIONARIO GESTIÓN DE CASOS

Buenos días / Buenas tardes:

A continuación procedo a realizarle unas preguntas, con la finalidad de obtener información útil para poder realizar su seguimiento y orientar los servicios de salud que pueda necesitar.

Todo el tratamiento de los datos obtenidos lo realizará, de manera confidencial, el equipo investigador.

Es muy importante que responda de manera concreta y con total sinceridad. Si no entiende alguna de las preguntas o de las respuestas, por favor, no dude en preguntar.

**START BACK SCREENING TOOL: DOLOR DE ESPALDA (9 preguntas)**

Piense en las últimas **2** semanas y marque su respuesta a las siguientes preguntas:

| | | Desacuerdo | De acuerdo |
|---|---|---|---|
| | | 0 | 1 |
| **1** | Mi dolor de espalda **se ha extendido a lo largo de mi pierna (s)** en alguna ocasión en las últimas dos semanas | ☐ | ☐ |
| **2** | Me ha dolido el **hombro** o **cuello** en alguna ocasión en las últimas dos semanas | ☐ | ☐ |
| **3** | En las últimas dos semanas, sólo he **caminado distancias cortas** por mi dolor de espalda | ☐ | ☐ |
| **4** | En las últimas dos semanas, me he **vestido más lentamente** de lo normal por mi dolor de espalda | ☐ | ☐ |
| **5** | No es seguro ser **físicamente activo** con mi dolor de espalda | ☐ | ☐ |
| **6** | Me he **preocupado** mucho por mi dolor de espalda en las últimas dos semanas | ☐ | ☐ |
| **7** | Noto que **mi dolor de espalda es terrible** y que **nunca irá a mejor** | ☐ | ☐ |
| **8** | En general en las últimas dos semanas, no he **disfrutado** de las cosas lo que habitualmente disfruto | ☐ | ☐ |

**9** En general, cómo le ha **molestado su espalda** en las últimas dos semanas:

| Nada | Un poco | Moderadamente | Mucho | Extremadamente |
|---|---|---|---|---|
| ☐ | ☐ | ☐ | ☐ | ☐ |
| 0 | 0 | 0 | 1 | 1 |

**SOMATIZACIÓN – CUPID (5 preguntas)**

A continuación, le recito una lista de cinco problemas que las personas normalmente tienen. Por favor, atienda cuidadosamente a cada uno y dígame **CUÁNTO LE HAN MOLESTADO O PREOCUPADO ESOS PROBLEMAS** durante los **ÚLTIMOS 7 DÍAS, INCLUYENDO EL DÍA DE HOY.**

| | | Nada | Muy poco | Algo | Bastante | Mucho |
|---|---|---|---|---|---|---|
| **10** | Desmayos o mareos | 0 | 1 | 2 | 3 | 4 |
| **11** | Dolores en el corazón o tórax | 0 | 1 | 2 | 3 | 4 |
| **12** | Náusea o molestia en el estómago | 0 | 1 | 2 | 3 | 4 |
| **13** | Problemas para respirar | 0 | 1 | 2 | 3 | 4 |
| **14** | Periodos de frío o calor | 0 | 1 | 2 | 3 | 4 |

**GHQ-12** (12 preguntas)

Ahora nos gustaría saber cómo se ha sentido, en general, **durante las últimas semanas.** Por favor, conteste a todas las preguntas indicando la respuesta que, a su juicio, mejor puede aplicarse a usted. Recuerde que sólo debe responder sobre los problemas recientes y los que tiene ahora, no sobre los que tuvo en el pasado.

| **15** | **¿Ha podido concentrarse bien en lo que hacía?** | | | | |
|---|---|---|---|---|---|
| Mejor que lo habitual | Igual que lo habitual | Menos que lo habitual | Mucho menos que lo habitual | No sabe | No contesta |
| 0 | 1 | 2 | 3 | 8 | 9 |

| **16** | **¿Sus preocupaciones le han hecho perder mucho sueño?** | | | | |
|---|---|---|---|---|---|
| No, en absoluto | No más que lo habitual | Algo más que lo habitual | Mucho más que lo habitual | No sabe | No contesta |
| 0 | 1 | 2 | 3 | 8 | 9 |

| **17** | **¿Ha sentido que está desempeñando un papel útil en la vida?** | | | | |
|---|---|---|---|---|---|
| Más útil que lo habitual | Igual que lo habitual | Menos útil que lo habitual | Mucho menos útil que lo habitual | No sabe | No contesta |
| 0 | 1 | 2 | 3 | 8 | 9 |

| **18** | **¿Se ha sentido capaz de tomar decisiones?** | | | | |
|---|---|---|---|---|---|
| Más que lo habitual | Igual que lo habitual | Menos que lo habitual | Mucho menos que lo habitual | No sabe | No contesta |
| 0 | 1 | 2 | 3 | 8 | 9 |

| **19** | **¿Se ha notado constantemente agobiado y en tensión?** | | | | |
|---|---|---|---|---|---|
| No, en absoluto | No más que lo habitual | Algo más que lo habitual | Mucho más que lo habitual | No sabe | No contesta |
| 0 | 1 | 2 | 3 | 8 | 9 |

| **20** | **¿Ha tenido la sensación de que no puede superar sus dificultades?** | | | | |
|---|---|---|---|---|---|
| No, en absoluto | No más que lo habitual | Algo más que lo habitual | Mucho más que lo habitual | No sabe | No contesta |
| 0 | 1 | 2 | 3 | 8 | 9 |

| **21** | **¿Ha sido capaz de disfrutar de sus actividades normales de cada día?** | | | | |
|---|---|---|---|---|---|
| Más que lo habitual | Igual que lo habitual | Menos que lo habitual | Mucho menos que lo habitual | No sabe | No contesta |
| 0 | 1 | 2 | 3 | 8 | 9 |

| **22** | **¿Ha sido capaz de hacer frente adecuadamente a sus problemas?** | | | | |
|---|---|---|---|---|---|
| Más capaz que lo habitual | Igual que lo habitual | Menos capaz que lo habitual | Mucho menos capaz que lo habitual | No sabe | No contesta |
| 0 | 1 | 2 | 3 | 8 | 9 |

| **23** | **¿Se ha sentido poco feliz o deprimido?** |
|---|---|

| No, en absoluto | No más que lo habitual | Algo más que lo habitual | Mucho más que lo habitual | No sabe | No contesta |
|---|---|---|---|---|---|
| 0 | 1 | 2 | 3 | 8 | 9 |

| 24 | **¿Ha perdido confianza en sí mismo?** | | | | |
|---|---|---|---|---|---|
| No, en absoluto | No más que lo habitual | Algo más que lo habitual | Mucho más que lo habitual | No sabe | No contesta |
| 0 | 1 | 2 | 3 | 8 | 9 |

| 25 | **¿Ha pensado que usted es una persona que no vale para nada?** | | | | |
|---|---|---|---|---|---|
| No, en absoluto | No más que lo habitual | Algo más que lo habitual | Mucho más que lo habitual | No sabe | No contesta |
| 0 | 1 | 2 | 3 | 8 | 9 |

| 26 | **¿Se siente razonablemente feliz considerando todas las circunstancias?** | | | | |
|---|---|---|---|---|---|
| Más que lo habitual | Igual que lo habitual | Menos que lo habitual | Mucho menos que lo habitual | No sabe | No contesta |
| 0 | 1 | 2 | 3 | 8 | 9 |

**EQ - 5D** (6 preguntas)

Seleccione la afirmación en cada sección que describa mejor su estado de salud en el día de hoy:

| 27 | **Movilidad** | | | | |
|---|---|---|---|---|---|
| No tengo problemas para caminar | ☐ | Tengo algunos problemas para caminar | ☐ | Tengo que estar en la cama | ☐ |

| 28 | **Cuidado personal** | | | | |
|---|---|---|---|---|---|
| No tengo problemas con el cuidado personal | ☐ | Tengo algunos problemas para lavarme o vestirme solo | ☐ | Soy incapaz de lavarme o vestirme solo | ☐ |

| 29 | **Actividades de <u>Todos los Días</u>** (ej, trabajar, estudiar, hacer tareas domésticas, actividades familiares o realizadas durante el tiempo libre) | | | | |
|---|---|---|---|---|---|
| No tengo problemas para realizar mis actividades de todos los días | ☐ | Tengo algunos problemas para realizar mis actividades de todos los días | ☐ | Son incapaz de realizar mis actividades de todos los días | ☐ |

| 30 | **Dolor/Malestar** | | | | |
|---|---|---|---|---|---|
| No tengo dolor ni malestar | ☐ | Tengo moderado dolor o malestar | ☐ | Tengo mucho dolor o malestar | ☐ |

| 31 | **Ansiedad/Depresión** | | | | |
|---|---|---|---|---|---|
| No estoy ansioso/a ni deprimido/a | ☐ | Estoy moderadamente ansioso/a o deprimido/a | ☐ | Estoy muy ansioso/a o deprimido/a | ☐ |

Para ayudar a la gente a describir lo bueno o lo malo que es su estado de salud, hemos dispuesto una escala parecida a un termómetro en el cual se marca con un 100 el mejor estado de salud que pueda imaginarse, y con un 0 el peor estado de salud que pueda imaginarse.

**32** Por favor, de 0 a 100, ¿cómo puntuaría **su estado de salud hoy?**

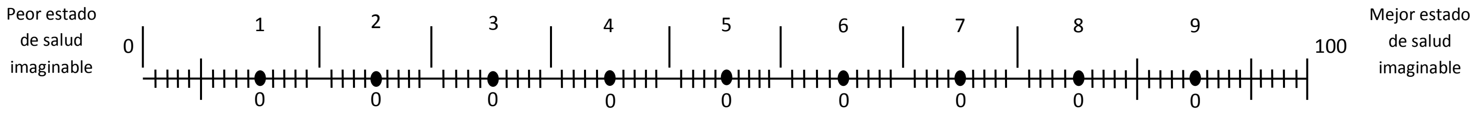


**AUTOEFICACIA** (8 preguntas)

| 33 | Puedo encontrar la forma de obtener lo que quiero aunque alguien se me oponga | | | | |
|---|---|---|---|---|---|
| Nunca o casi nunca | Solo una vez | Algunas veces | Muchas veces | Siempre | NS/NC |
| ☐ | ☐ | ☐ | ☐ | ☐ | ☐ |

| 34 | Puedo resolver problemas difíciles si me esfuerzo lo suficiente | | | | |
|---|---|---|---|---|---|
| Nunca o casi nunca | Solo una vez | Algunas veces | Muchas veces | Siempre | NS/NC |
| ☐ | ☐ | ☐ | ☐ | ☐ | ☐ |

| 35 | Me es fácil persistir en lo que me he propuesto hasta llegar a alcanzar mis metas | | | | |
|---|---|---|---|---|---|
| Nunca o casi nunca | Solo una vez | Algunas veces | Muchas veces | Siempre | NS/NC |
| ☐ | ☐ | ☐ | ☐ | ☐ | ☐ |

| 36 | Tengo confianza en que podría manejar eficazmente acontecimientos inesperados | | | | |
|---|---|---|---|---|---|
| Nunca o casi nunca | Solo una vez | Algunas veces | Muchas veces | Siempre | NS/NC |
| ☐ | ☐ | ☐ | ☐ | ☐ | ☐ |

| 37 | Gracias a mis cualidades y recursos puedo superar situaciones imprevistas | | | | |
|---|---|---|---|---|---|
| Nunca o casi nunca | Solo una vez | Algunas veces | Muchas veces | Siempre | NS/NC |
| ☐ | ☐ | ☐ | ☐ | ☐ | ☐ |

| 38 | Puedo resolver la mayoría de los problemas si me esfuerzo lo necesario | | | | |
|---|---|---|---|---|---|
| Nunca o casi nunca | Solo una vez | Algunas veces | Muchas veces | Siempre | NS/NC |
| ☐ | ☐ | ☐ | ☐ | ☐ | ☐ |

| 39 | Si me encuentro en una situación difícil, generalmente se me ocurre qué debo hacer | | | | |
|---|---|---|---|---|---|
| Nunca o casi nunca | Solo una vez | Algunas veces | Muchas veces | Siempre | NS/NC |

| ☐ | ☐ | ☐ | ☐ | ☐ | ☐ |
|---|---|---|---|---|---|

| **40** | **Al tener que hacer frente a un problema, generalmente se me ocurren varias alternativas de cómo resolverlo** | | | | |
|---|---|---|---|---|---|
| Nunca o casi nunca | Solo una vez | Algunas veces | Muchas veces | Siempre | NS/NC |
| ☐ | ☐ | ☐ | ☐ | ☐ | ☐ |

**HÁBITOS SALUDABLES** (9 preguntas)

| **41** | **¿Cuántas horas a la semana realiza ejercicio físico (hacer gimnasia, natación, dar paseos…) en su tiempo libre?** | | | |
|---|---|---|---|---|
| No realiza ejercicio físico (Salto a la pregunta 43) | Menos de dos horas a la semana | Entre 2 y 4 horas a la semana | Más de 4 horas a la semana | NS/NC |
| ☐ | ☐ | ☐ | ☐ | ☐ |

| **42** | **¿Qué tipo de ejercicio físico realiza en su tiempo libre?** | | |
|---|---|---|---|
| De intensidad leve (ej. Andar) | De intensidad moderada (ej. natación, tenis) | De intensidad alta (ej. gimnasia de competición) | NS/NC |
| ☐ | ☐ | ☐ | ☐ |

| **43** | **¿Ha habido alguna vez un periodo de su vida, en que usted haya fumado tabaco todos los días?** | | |
|---|---|---|---|
| Sí, alguna vez he fumado todos los días | No he fumado en ninguna ocasión todos los días | Nunca he fumado un cigarrillo u otro tipo de tabaco (Salto a la pregunta 46) | NS/NC |
| ☐ | ☐ | ☐ | ☐ |

| **44** | **¿Cuánto tiempo ha pasado desde la última vez que usted fumó un cigarrillo u otro tipo de tabaco?** | | |
|---|---|---|---|
| Menos de 30 días | Más de 1 mes pero menos de un año | Más de 12 meses (Salto a la pregunta 46) | NS/NC |
| ☐ | ☐ | ☐ | ☐ |

| **45** | **Durante los últimos 30 días, por término medio, ¿qué número de cigarrillos, puros o pipas solía fumar cada día que fumaba? Dígame el número medio o seleccione la opción correspondiente.** | |
|---|---|---|
| No he fumado un cigarrillo u otro tipo de tabaco en los últimos 30 días | | NS/NC |
| ☐ | | ☐ |
| Nº cigarrillos: | Nº puros: | Nº pipas: |

| **46** | **¿Cuántas horas calcula que habrá dormido verdaderamente cada noche durante el último mes?** |
|---|---|
| Horas: ………………… | NS/NC ☐ |

| **47** | **Durante el último mes, cuántas veces ha tenido usted problemas para dormir a causa de: No poder conciliar el sueño en la primera media hora** | | | |
|---|---|---|---|---|
| Ninguna en el último mes | Menos de una vez a la semana | Una o dos veces a la semana | Tres o más veces a la semana | NS/NC |

| | | | | |
|---|---|---|---|---|
| ☐ | ☐ | ☐ | ☐ | ☐ |

| 48 | **Durante el último mes, cuántas veces ha tenido usted problemas para dormir a causa de: Despertarse durante la noche o madrugada** | | | |
|---|---|---|---|---|
| Ninguna en el último mes | Menos de una vez a la semana | Una o dos veces a la semana | Tres o más veces a la semana | NS/NC |
| ☐ | ☐ | ☐ | ☐ | ☐ |

| 49 | **Durante el último mes, ¿cómo valoraría, en conjunto, la calidad de su sueño?** | | | |
|---|---|---|---|---|
| Muy buena | Bastante buena | Bastante mala | Muy mala | NS/NC |
| ☐ | ☐ | ☐ | ☐ | ☐ |

**COMORBILIDAD** (1 pregunta)

| 50 | **¿Padece o ha padecido alguna enfermedad o problema de salud de larga duración, es decir, que haya durado o que se espere que dure 6 meses o más? En caso afirmativo, ¿cuánto le interfiere en el desarrollo de sus actividades diarias? en una escala de 1 (no me limita) a 5 (me limita mucho)** | | | | | |
|---|---|---|---|---|---|---|
| | | 1 | 2 | 3 | 4 | 5 |
| ☐ | Alergia | ☐ | ☐ | ☐ | ☐ | ☐ |
| ☐ | Angina de pecho | ☐ | ☐ | ☐ | ☐ | ☐ |
| ☐ | Ansiedad relacionada con el estrés | ☐ | ☐ | ☐ | ☐ | ☐ |
| ☐ | Artritis/osteoartritis | ☐ | ☐ | ☐ | ☐ | ☐ |
| ☐ | Asma | ☐ | ☐ | ☐ | ☐ | ☐ |
| ☐ | Cáncer | ☐ | ☐ | ☐ | ☐ | ☐ |
| ☐ | Patología cardíaca isquémica | ☐ | ☐ | ☐ | ☐ | ☐ |
| ☐ | Patología hepática | ☐ | ☐ | ☐ | ☐ | ☐ |
| ☐ | Patología renal | ☐ | ☐ | ☐ | ☐ | ☐ |
| ☐ | EPOC (enfermedad pulmonar obstructiva crónica) | ☐ | ☐ | ☐ | ☐ | ☐ |
| ☐ | Depresión | ☐ | ☐ | ☐ | ☐ | ☐ |
| ☐ | Ansiedad | ☐ | ☐ | ☐ | ☐ | ☐ |
| ☐ | Diabetes | ☐ | ☐ | ☐ | ☐ | ☐ |
| ☐ | Epilepsia | ☐ | ☐ | ☐ | ☐ | ☐ |
| ☐ | Arritmia | ☐ | ☐ | ☐ | ☐ | ☐ |
| ☐ | Insuficiencia cardíaca | ☐ | ☐ | ☐ | ☐ | ☐ |
| ☐ | Colesterol alto | ☐ | ☐ | ☐ | ☐ | ☐ |
| ☐ | Hipertensión | ☐ | ☐ | ☐ | ☐ | ☐ |
| ☐ | Dolor lumbar (lumbago/ciática) | ☐ | ☐ | ☐ | ☐ | ☐ |

| | | | | | | |
|---|---|---|---|---|---|---|
| ☐ | Dolor cervical | ☐ | ☐ | ☐ | ☐ | ☐ |
| ☐ | Infarto de miocardio | ☐ | ☐ | ☐ | ☐ | ☐ |
| ☐ | Osteoporosis | ☐ | ☐ | ☐ | ☐ | ☐ |
| ☐ | Íctus (accidente cerebrovascular) | ☐ | ☐ | ☐ | ☐ | ☐ |
| ☐ | Trastorno tiroideo | ☐ | ☐ | ☐ | ☐ | ☐ |
| ☐ | NS/NC | ☐ | ☐ | ☐ | ☐ | ☐ |

## Annexe ANEXO 5: PAUTA PARA EL CIERRE DE CASOS Y PERIODICIDAD DEL SEGUIMIENTO MOTIVACIONAL

**Pauta cierre de casos y periodicidad del seguimiento motivacional**

1. **Cierre de casos**

Trabajador de bajo riesgo sin IT se estima que el cierre se realizará aproximadamente un mes después del primer contacto, y en el caso de trabajador de bajo riesgo con IT, si éste recibe el alta antes de un mes se actuará como el caso anterior, y en el caso de que un trabajador de bajo riesgo siga en IT al cabo de un mes, se valorará el caso en la sesión de casos.

Trabajador de riesgo medio o alto, se cerrará el caso cuando se cumpla al menos uno de los siguientes supositos: los profesionales de los servicios consideren que el trabajador ya no necesita más sesiones; en la sesión de casos semanal se valore que su limitación funcional ha desaparecido/disminuido y que no necesita más sesiones; o bien, cuando él mismo decida dejar de participar (independientemente de si es por mejora o no).

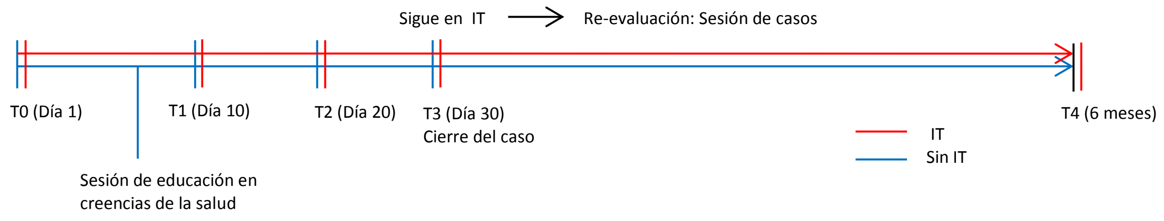


2. **Seguimiento motivacional.**

En trabajadores de bajo riesgo se estiman unos 5 contactos telefónicos: T0 (día 1), 1r contacto (entrevista y cuestionario); T1 (día 10), 2ndo contacto (posterior a la sesión de educación en creencias de la salud); T2 (día 20), 3r contacto (seguimiento motivacional, recordatorio creencias); T3 (día 30), 4to contacto (cierre del caso), aproximadamente 1 mes después del primer contacto); T4, 5to contacto (cuestionario 6 meses posterior al cierre del caso).

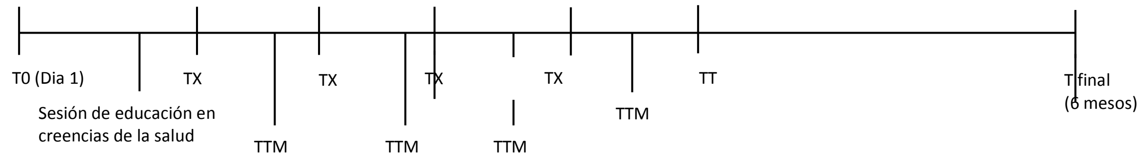


En trabajadores de riesgo medio o alto el número de contactos telefónicos variará según la duración de la pauta de tratamiento y según valoración: T0 (día 1), 1r contacto (entrevista y cuestionario); T1 (día 10)- TX, contactos periódicos según pauta de tratamiento; TT (día), cierre del caso, cuestionario; Tfinal, cuestionario 6 meses posterior al cierre del caso.

## ANEXO 6: INFORME DEL COMITÉ ÉTICO DE INVESTIGACIÓN CLÍNICA

Parc de Salut MAR Barcelona

Dra. ANA MARÍA GARCÍA GARCÍA
Universitat Pompeu Fabra
Centre d'Investigació en Salut Laboral (CiSAL)
Doctor Aiguader, 88
08003 Barcelona

Benvolguda Dra. GARCÍA,

El CEIC - Parc de Salut Mar una vegada avaluats el projecte de recerca núm. 2014/5714/I titulat *"Evaluación de una intervención multifactorial en el lugar de trabajo para la prevención de trastornos musculoesqueléticos en los trabajadores (INTEVAL_Spain)"*, li comunica que ha obtingut l'aprovació. Li adjuntem el certificat corresponent.

Cordialment,

COMITÈ ÈTIC D'INVESTIGACIÓ CLÍNICA
CEIC - PARC DE SALUT MAR

Mª Teresa Navarra Alcrudo
Secretaria CEIC - Parc de Salut Mar

Barcelona, a 10 de juliol de 2014

**CEIC – Parc de Salut MAR**
Dr. Aiguader, 88 I 08003 Barcelona I Telèfon 93 316 06 77 I Fax 93 316 06 36
ceic-psmar@imim.es | www.parcdesalutmar.cat

Parc de Salut Barcelona MAR

## Informe del Comité Ético de Investigación Clínica

Doña Mª Teresa Navarra Alcrudo Secretaria del Comité Ético de Investigación Clínica del Parc de Salut Mar

### CERTIFICA

Que éste Comité ha evaluado el proyecto de investigación clínica nº 2014/5714/I titulado *"Evaluación de una intervención multifactorial en el lugar de trabajo para la prevención de trastornos musculoesqueléticos en los trabajadores (INTEVAL_Spain)"*, propuesto por la Dra. ANA MARÍA GARCÍA GARCÍA, de la Universitat Pompeu Fabra de Barcelona.

Y que considera que:

Se cumplen los requisitos necesarios de idoneidad del protocolo en relación con los objetivos del estudio y están justificados los riesgos y molestias previsibles para el sujeto.

La capacidad del investigador y los medios disponibles son apropiados para llevar a cabo el estudio.

El alcance de las compensaciones económicas que se solicitan están plenamente justificadas.

Y que éste Comité acepta que dicho proyecto de investigación sea realizado en la Universitat Pompeu Fabra por la Dra. ANA MARÍA GARCÍA GARCÍA, como investigador principal tal como recoge el ACTA de la reunión del día 8 de Julio de 2014.

Lo que firmo en Barcelona, a 10 de Julio de 2014

COMITÈ ÈTIC D'INVESTIGACIÓ CLÍNICA
CEIC - PARC DE SALUT MAR

Firmado: ....................................
Doña Mª Teresa Navarra Alcrudo

CEIC – Parc de Salut MAR
Dr. Aiguader, 88 I 08003 Barcelona I Telèfon 93 316 06 77 I Fax 93 316 06 36
ceic-psmar@imim.es | www.parcdesalutmar.cat
